# Supplementary material for: Genome-Wide Identification of Gramineae Brassinosteroid-Related Genes and Their Roles in Plant Architecture and Salt Stress Adaptation
Source: Int J Mol Sci. 2022 May 16;23(10):5551. doi: 10.3390/ijms23105551 (PMC9146025; doi:10.3390/ijms23105551)

Supplemental Figure S1 Chromosomal location of BR-related plant architecture genes in *T. aestivum*, *H. vulgare*, *Z. mays* and *S. bicolor*.

Supplemental Figure S1-1 Chromosomal location of BR-related plant architecture genes in *T. aestivum*.

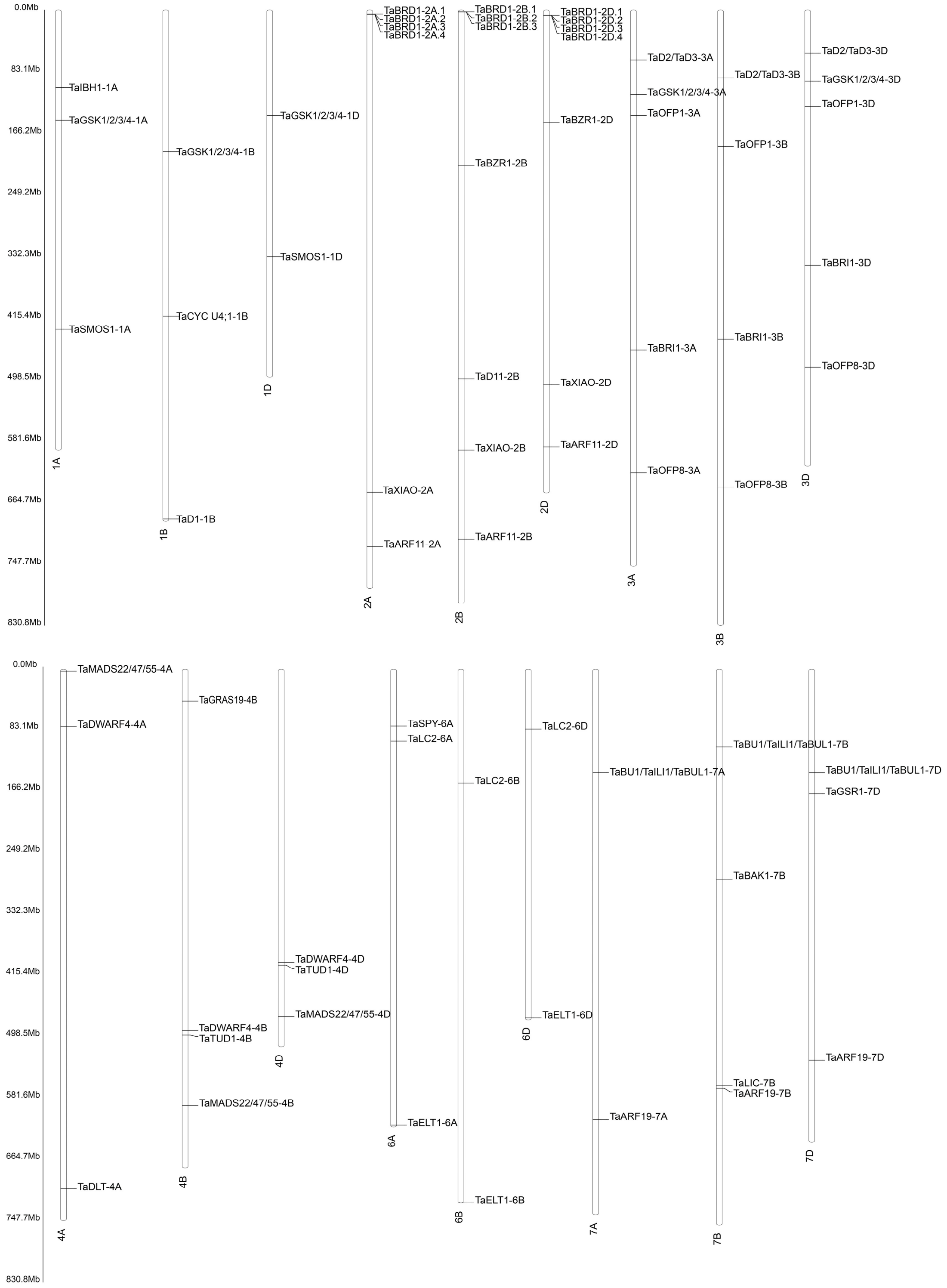

Supplemental Figure S1-2 Chromosomal location of BR-related plant architecture genes in *Z. mays*.

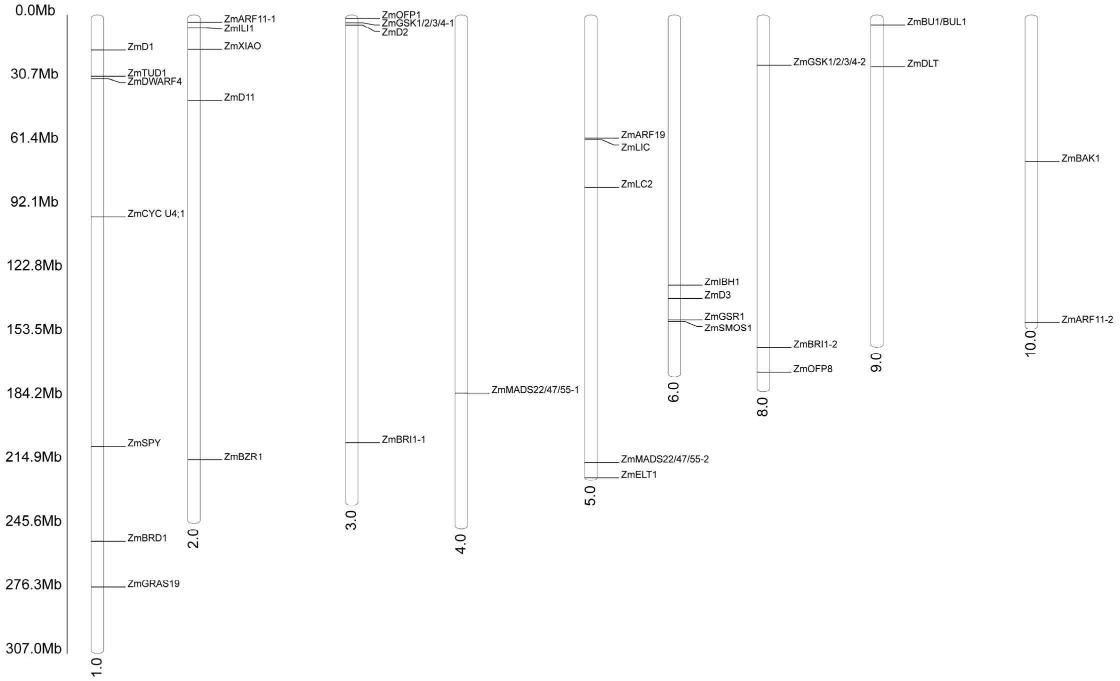

Supplemental Figure S1-3 Chromosomal location of BR-related plant architecture genes in *H. vulgare*.

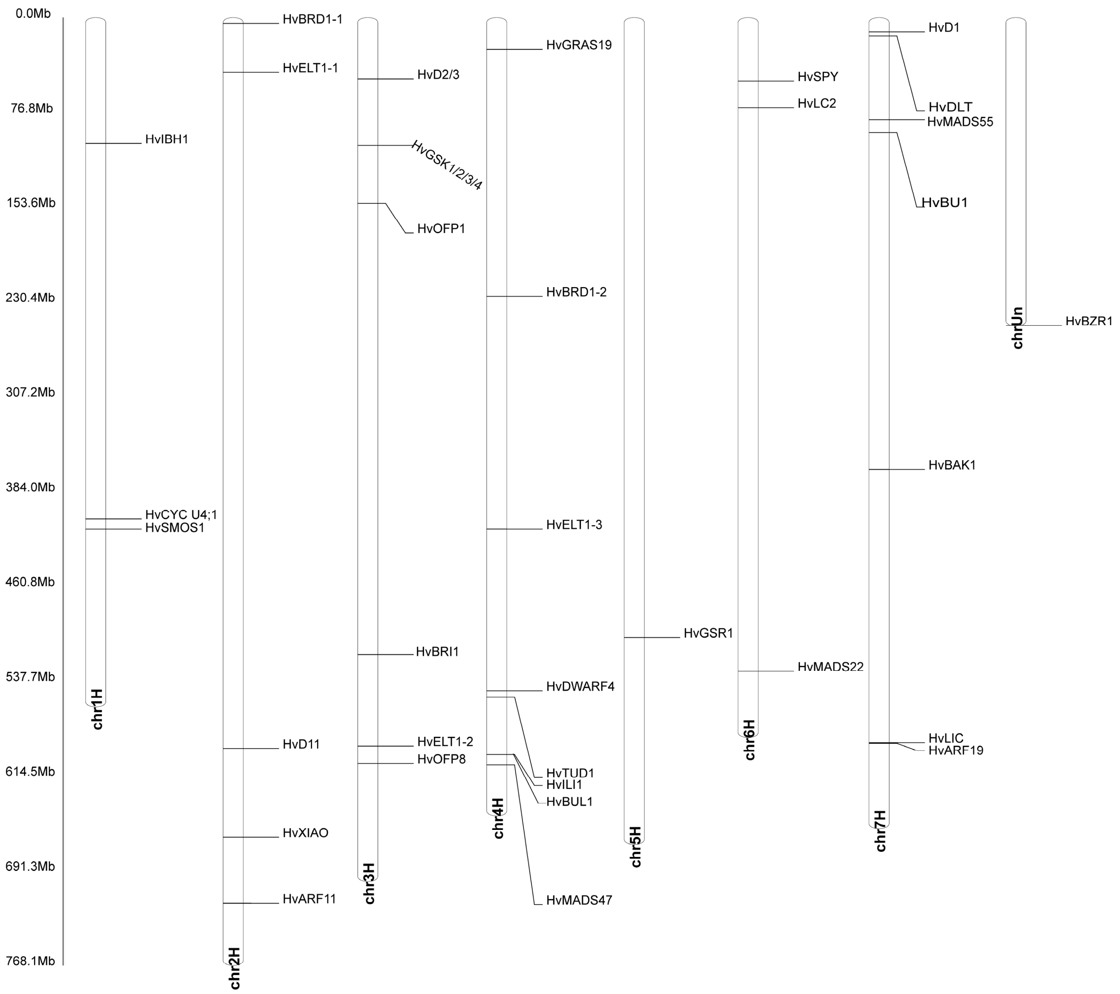

Supplemental Figure S1-4 Chromosomal location of BR-related plant architecture genes in *S. bicolor*.

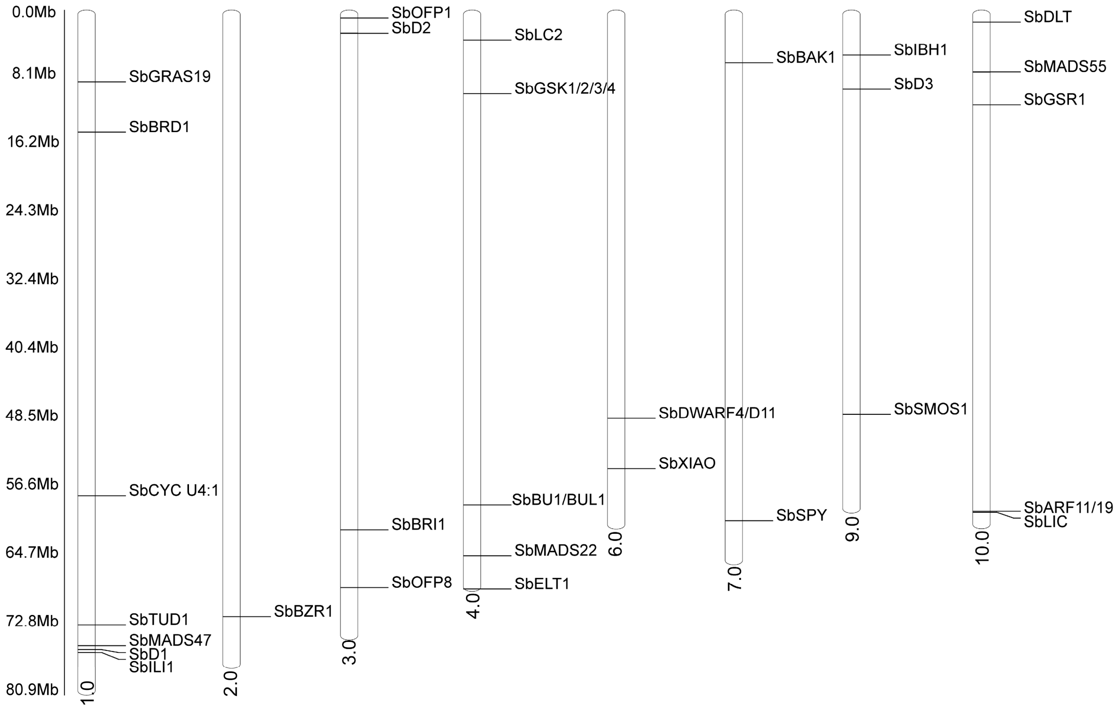

Supplement: Supplementary file 1 [file ijms-23-05551-s001.zip › Figure S1.pdf]
