# Supplementary material for: Genome-Wide Identification of Gramineae Brassinosteroid-Related Genes and Their Roles in Plant Architecture and Salt Stress Adaptation
Source: Int J Mol Sci. 2022 May 16;23(10):5551. doi: 10.3390/ijms23105551 (PMC9146025; doi:10.3390/ijms23105551)

Supplemental Figure S2 Alignment of multiple BR-related plant architecture proteins in rice, *T. aestivum*, *H. vulgare*, *Z. mays* and *S. bicolor*.

Supplemental Figure S2-1 Alignment of multiple DWARF4 proteins in rice, *T. aestivum*, *H. vulgare*, *Z. mays* and *S. bicolor*.

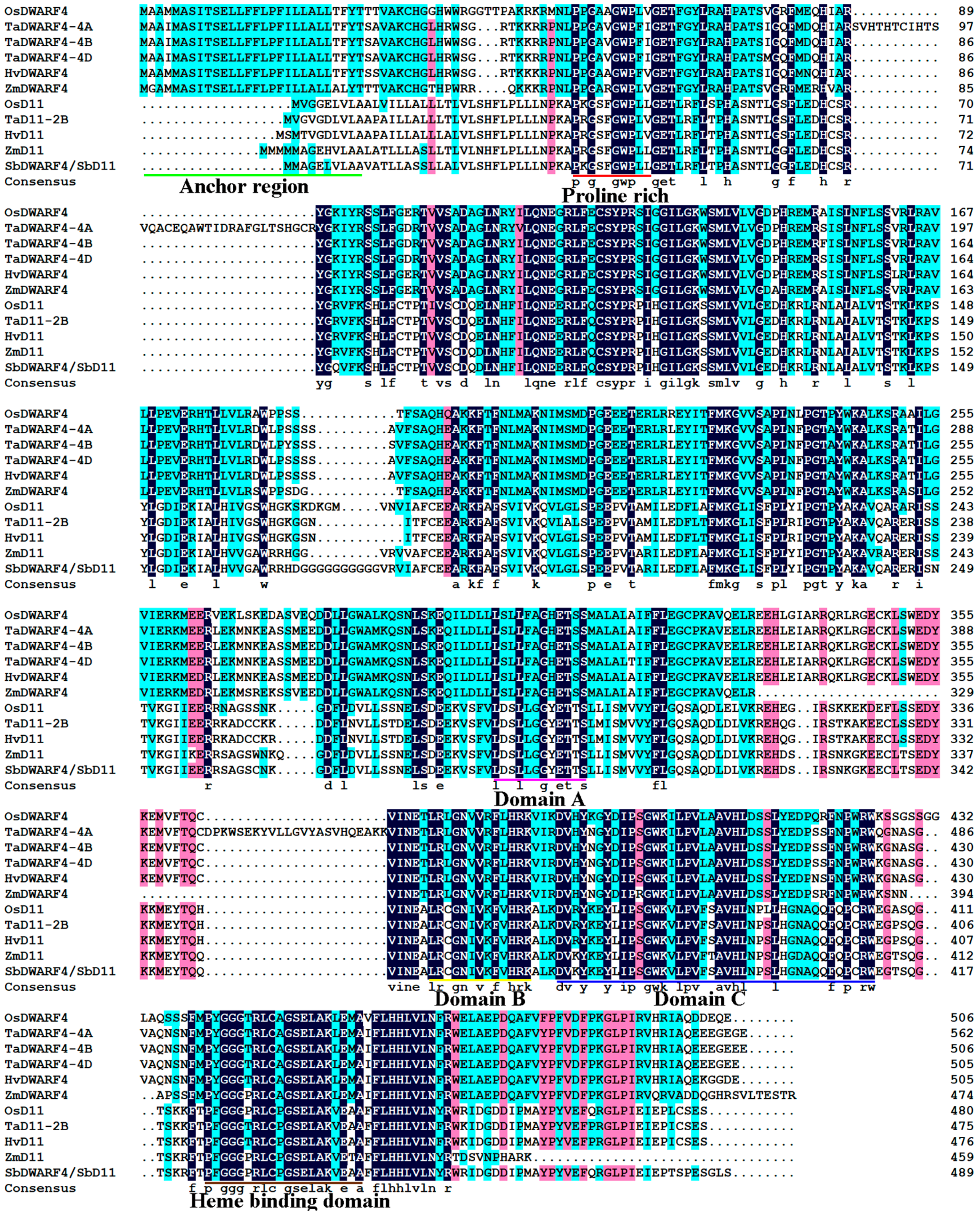

Supplemental Figure S2-2 Alignment of multiple D2 and D3 proteins in rice, *T. aestivum*, *H. vulgare*, *Z. mays* and *S. bicolor*.

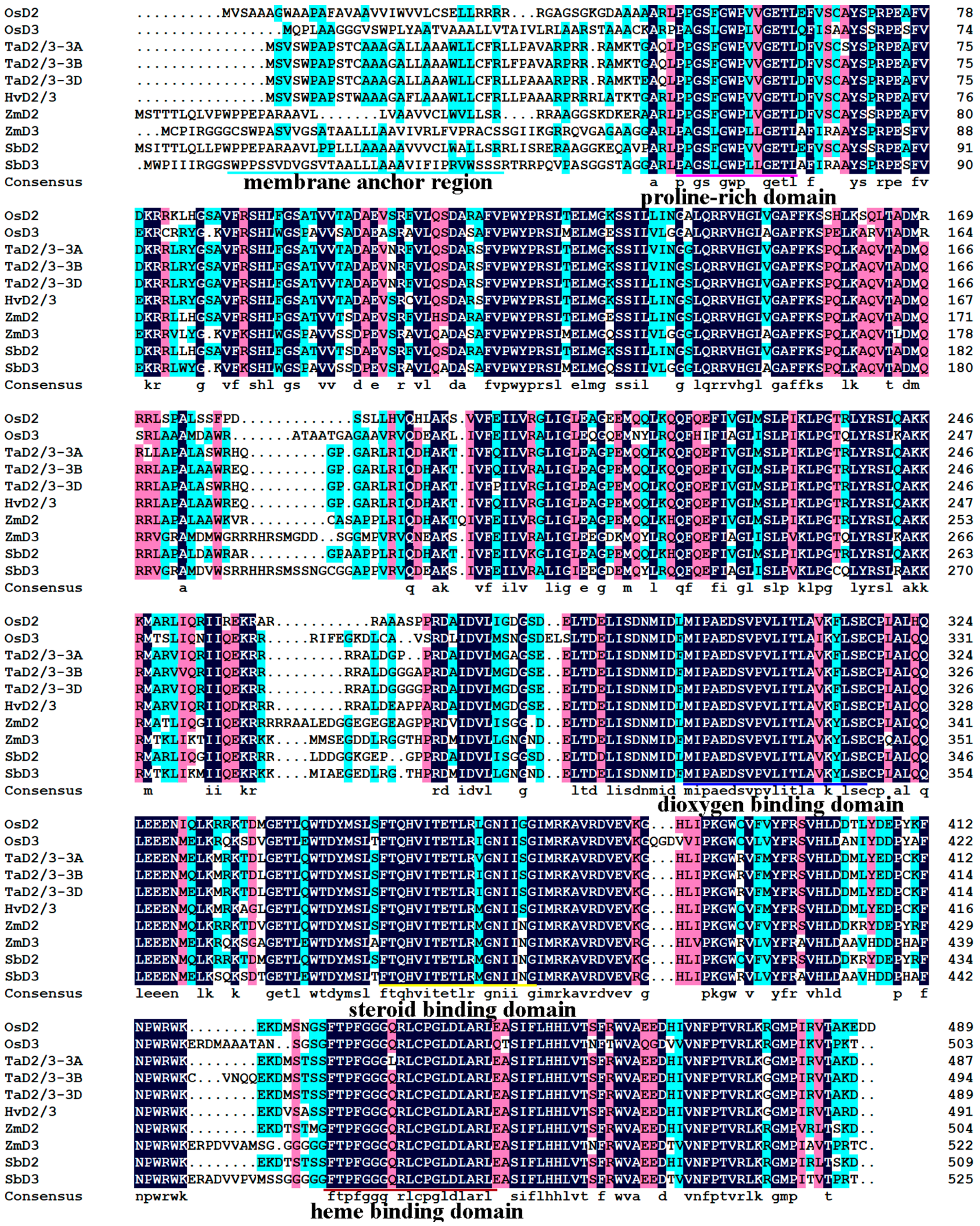

Supplemental Figure S2-3 Alignment of multiple BRD1 proteins in rice, *T. aestivum*, *H. vulgare*, *Z. mays* and *S. bicolor*.

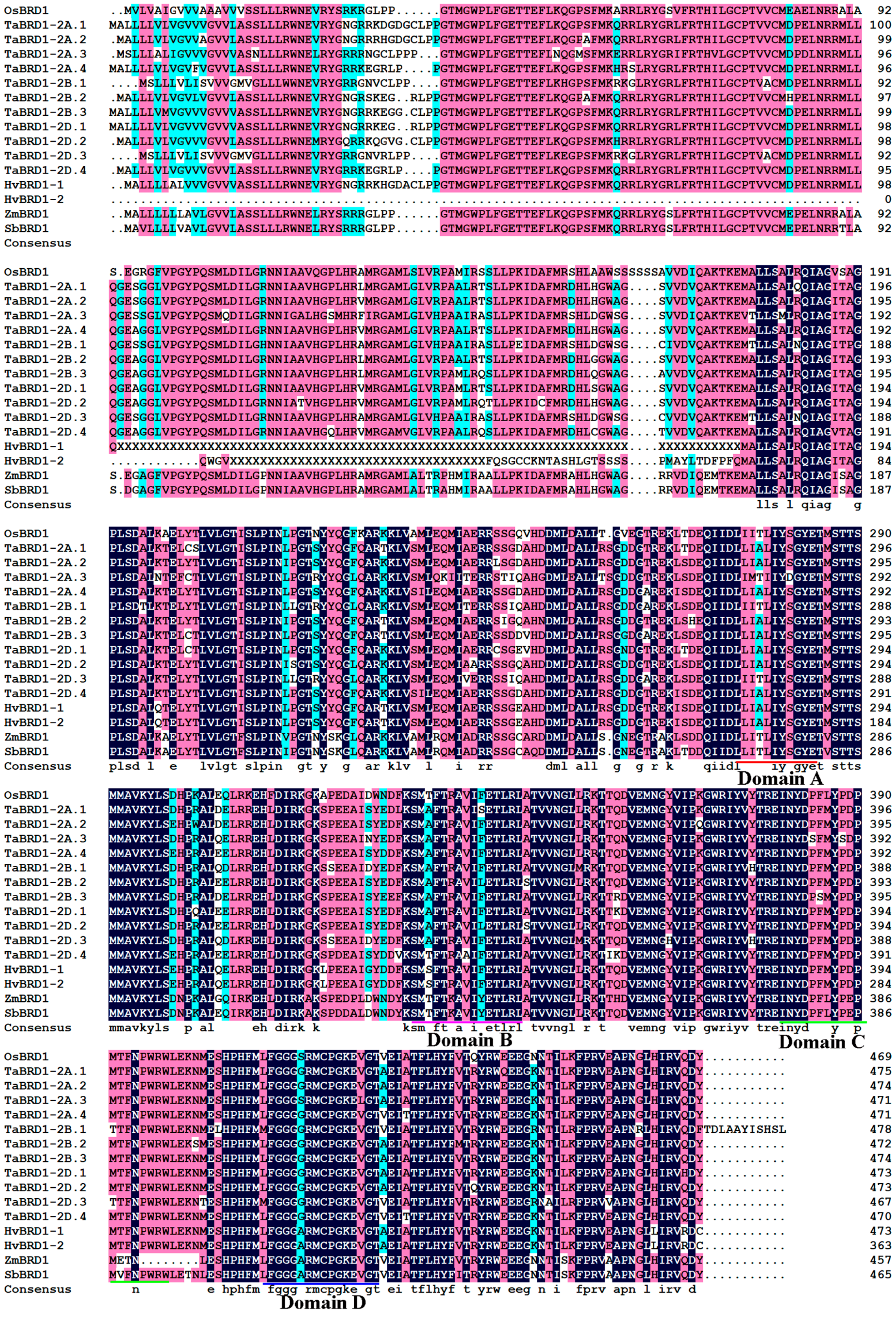

Supplemental Figure S2-4 Alignment of multiple BRI1 proteins in rice, *T. aestivum*, *H. vulgare*, *Z. mays* and *S. bicolor*.

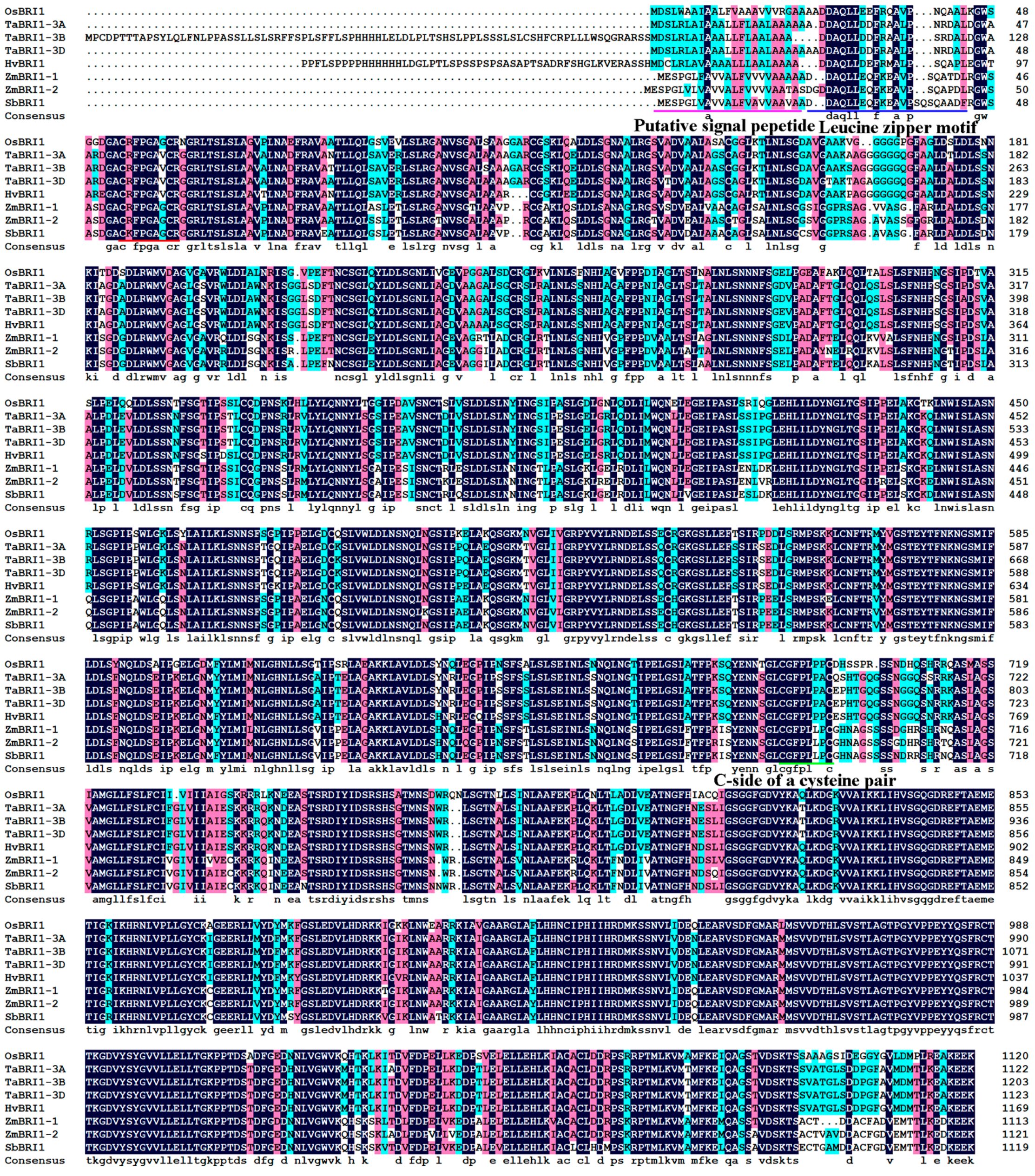

Supplemental Figure S2-5 Alignment of multiple BAK1 proteins in rice, *T. aestivum*, *H. vulgare*, *Z. mays* and *S. bicolor*.

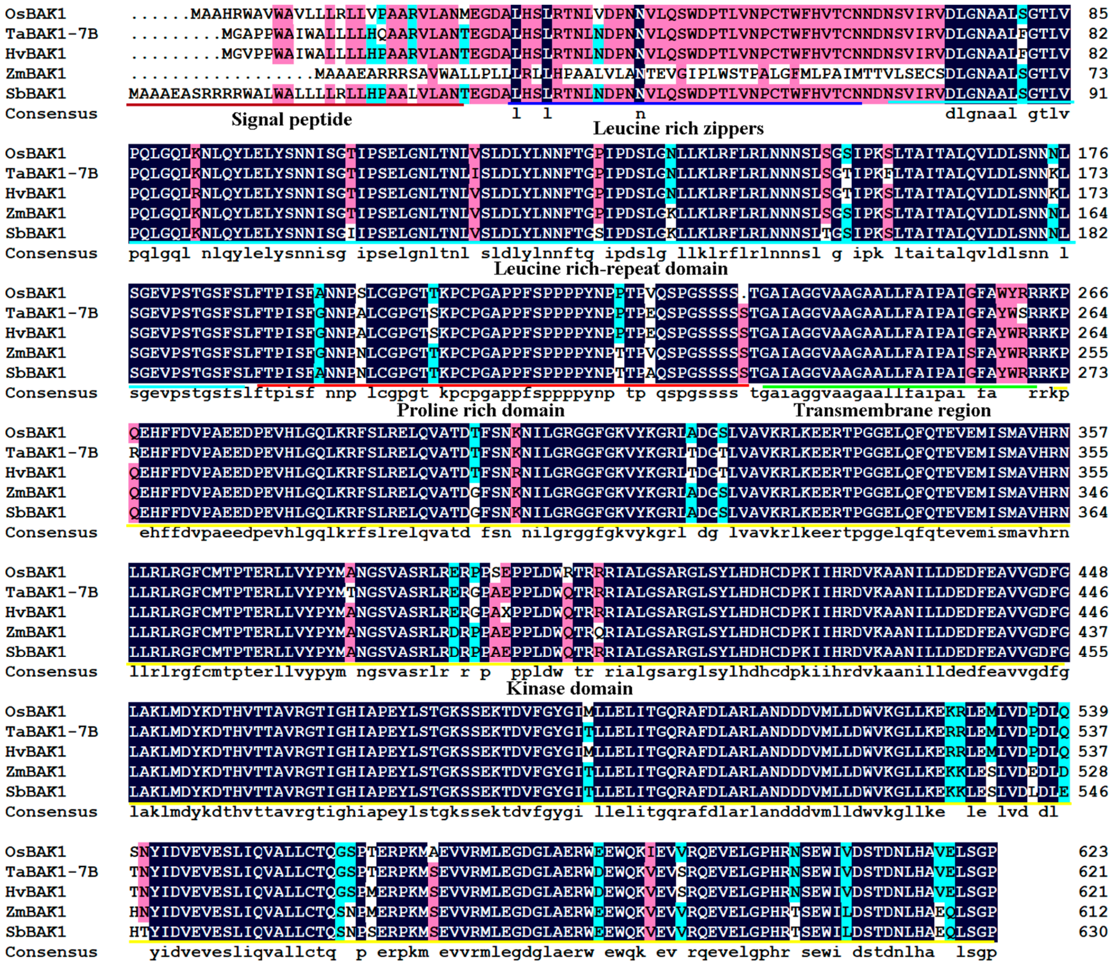

Supplemental Figure S2-6 Alignment of multiple GSK1 proteins in rice, *T. aestivum*, *H. vulgare*, *Z. mays* and *S. bicolor*.

```
OsGSK1              ...........................................................................   0
OsGSK2              .........MDQPAPAPEPMLLDAQPP.....AAVACDKKQQEGEAPYAEGNDAVTGHIISTTIGGKNGEPKRTI  61
OsGSK3              .........MDQPAPAPEPMLLDAQPP.....AAVACDKKQQEGEAPYAEGNDAVTGHIISTTIGGKNGEPKRTI  61
OsGSK4              ......MAAMPGGPDLAGAGGAVAVAVDAMQVDDPPRASAEEKHGPTIMGGNDPVTGHIISTTIGGKNDEPKRTI  69
TaGSK1/2/3/4-1A     .........MEHPAPAPEPMLLDEQPP.....TAVACEKKQQDGEAPYAEGNDAMTGHIISTTIGGKNGEPKQTI  61
TaGSK1/2/3/4-1B     .........MEHSAPAPEPMLLDEQPP.....TAVACEKKQQDGEAPYAEGNDAMTGHIISTTIGGKNGEPKQTI  61
TaGSK1/2/3/4-1D     .........MEHPAPAPEPMLLDEQPP.....TAVACEKKQQDGEAPYAEGNDAMTGHIISTTIGGKNGEPKQTI  61
TaGSK1/2/3/4-3A     ......................................MLRALVLQARTEGGDPVTGHIISTTIGGKNGEPKRTI  37
TaGSK1/2/3/4-3D     ..........MEAPPGPEPMVLDAPPPX.....XXXXXXXXXXXXXXXXXXXXXXXXXXXXXXXXXXNGEPKRTI  60
HvGSK1/2/3/4        ..........MDAPPGPEPMVLDAPPP......LAAAVPAHVVAEKARTEGGDPVTGHIISTTIGGKNGEPKRTI  59
ZmGSK1/2/3/4-1      ..........MEAPPGPGPMELDAPPP.....AAAAAIPPAGSDKHKDGEGGDAVTGHIISTTIGGKNGEPKRTI  61
ZmGSK1/2/3/4-2      ..........MEAPPVPELMDLDAPPPAAADAAAAAPVPPAVSDKKKEGEGGDTVTGHIISTTIGGKNGEPKRTI  65
SbGSK1/2/3/4        MAAPPGGAHAGAGAADPMQVDQPLPGAAAAAAAAAAHGPADAKHAGSMIEGSDPVTGHIISTTIGGKNGEPKRTI  75
Consensus

OsGSK1              ...........................................................................   0
OsGSK2              SYMAERVVGTGSFGIVFQAKCLETGETVAIKKVLQDRRYKNRELQLMRAMDHPNVISLKHCFFSTTSRDELFLNL 136
OsGSK3              SYMAERVVGTGSFGIVFQAKCLETGETVAIKKVLQDRRYKNRELQLMRAMDHPNVISLKHCFFSTTSRDELFLNL 136
OsGSK4              SYMAERVVGTGSFGVVFQAKCLETGETVAIKKVLQDKRYKNRELQIMRSMDHCNVISLKHCFFSTTSRDELFLNL 144
TaGSK1/2/3/4-1A     SYMAERVVGTGSFGIVFQAKCLETGETVAIKKVLQDRRYKNRELQLMRSMIHSNVVSLKHCFFSTTSRDELFLNL 136
TaGSK1/2/3/4-1B     SYMAERVVGTGSFGIVFQAKCLETGETVAIKKVLQDRRYKNRELQLMRSMIHSNVVSLKHCFFSTTSRDELFLNL 136
TaGSK1/2/3/4-1D     SYMAERVVGTGSFGIVFQAKCLETGEMVAIKKVLQDRRYKNRELQLMRSMIHSNVVSLKHCFFSTTSRDELFLNL 136
TaGSK1/2/3/4-3A     SYMAERVVGTGSFGIVFQAKCLETGETVAIKKVLQDRRYKNRELQLMRSMDHPNVVSLKHCFFSTTSRDELFLNL 112
TaGSK1/2/3/4-3D     SYMAERVVGTGSFGIVFQAKCLETGETVAIKKVLQDRRYKNRELQLMRSMDHPNVVSLKHCFFSTTSRDELFLNL 135
HvGSK1/2/3/4        SYMAERVVGTGSFGIVFQAKCLETGETVAIKKVLQDRRYKNRELQLMRSMDHPNVVSLKHCFFSTTSRDELFLNL 134
ZmGSK1/2/3/4-1      SYMAERVVGTGSFGIVFQAKCLETGETFAIKKVLQDRRYKNRELQLMRAMEHPNVICLKHCFFSTTSRDELFLNL 136
ZmGSK1/2/3/4-2      SYMAERVVGTGSFGIVFQAKCLETGETFAIKKVLQDRRYKNRELQLMRAMEHPNVICLKHCFFSTTSRDELFLNL 140
SbGSK1/2/3/4        SYMAERVVGTGSFGIVFQAKCLETGETVAIKKVLQDKRYKNRELQIMRSMDHCNVVSLKHCFFSTTSRDELFLNL 150
Consensus

OsGSK1              ...........................................................................   0
OsGSK2              VMEYVPETLYRVLKHYSNANHRMPLIYVKLYMYQLFRGLAYIHTVPGVCHRDVKPQ................... 192
OsGSK3              VMEYVPETLYRVLKHYSNANHRMPLIYVKLYMYQLFRGLAYIHTVPGVCHRDVKPQ................... 192
OsGSK4              VMEFVPESLYRVLKHYKDMKQRMPLIYVKLYMYQIFRGLAYIHTVPGVCHRDIKPQ................... 200
TaGSK1/2/3/4-1A     VMEYVPETLYRVLKHYSNAKQGMPLIYVKLYTYQLFRGLAYIHTVPGVCHRDVKPQ................... 192
TaGSK1/2/3/4-1B     VMEYVPETLYRVLKHYSNAKQGMPLIYVKLYTYQLFRGLAYIHTVPGVCHRDVKPQ................... 192
TaGSK1/2/3/4-1D     VMEYVPETLYRVLKHYSNAKQGMPLIYVKLYTYQLFRGLAYIHTVPGVCHRDVKPQ................... 192
TaGSK1/2/3/4-3A     VMEYVPETLYRVLKHYSNANQRMPLIYVKLYMYQLFRGLAYVHTVPGVCHRDVKPQ................... 168
TaGSK1/2/3/4-3D     VMEYVPETLYRVLKHYSNANQRMPLIYVKLYMYQLFRGLAYVHTVPGVCHRDVKPQ................... 191
HvGSK1/2/3/4        VMEYVPETLYRVLKHYSNANQRMPLIYVKLYMYQLFRGLAYVHTVPGVCHRDVKPQ................... 190
ZmGSK1/2/3/4-1      VMEFVPETLYRVLKHYSNANQRMPLIYVKLYMYQLFRGLAYIHNVPGVCHRDVKPQNVLVCVMWTRICSSFDLVI 211
ZmGSK1/2/3/4-2      VMEFVPETLYRVLKHYSNANQRMPLIYVKLYMYQLFRGLAYIHNVPGVCHRDVKPQ................... 196
SbGSK1/2/3/4        VMEFVPESLYRVLKHYSNMNQRMPLIYVKLYTYQIFRGLAYIHTVPGVCHRDVKPQ................... 206
Consensus
```

Serine/threonine kinase domains

```
OsGSK1              ............MVDPLTHQVKLCDFGSAKVLVPGEPNISYICSRYYRAPELIFGATEYTTSIDIWSAGCVLAEL  63
OsGSK2              ..........NVLVDPLTHQVKLCDFGSAKTLVPGEPNISYICSRYYRAPELIFGATEYTTSIDIWSAGCVLAEL 257
OsGSK3              ..........NVLVDPLTHQVKLCDFGSAKTLVPGEPNISYICSRYYRAPELIFGATEYTTSIDIWSAGCVLAEL 257
OsGSK4              ..........NILVDPLTHQVKVCDFGSAKMLIKGEANISYICSRYYRAPELIFGATEYTTSIDIWSAGCVLAEL 265
TaGSK1/2/3/4-1A     ..........NVLVDPLTHQVKICDFGSAKVLVAGEPNISYICSRYYRAPELIFGATEYTTSIDIWSAGCVLAEL 257
TaGSK1/2/3/4-1B     ..........NVLVDPLTHQVKICDFGSAKVLVAGEPNISYICSRYYRAPELIFGATEYTISIDIWSAGCVLAEL 257
TaGSK1/2/3/4-1D     ..........NVLVDPLTHQVKICDFGSAKVLVAGEPNISYICSRYYRAPELIFGATEYTTSIDIWSAGCVLAEL 257
TaGSK1/2/3/4-3A     ..........NVLVDPLTHQVKICDFGSAKVLVPGEPNIAYICSRYYRAPELIFGATEYTTSIDIWSAGCVLAEL 233
TaGSK1/2/3/4-3D     ..........NVLVDPLTHQVKICDFGSAKVLVPGEPNIAYICSRYYRAPELIFGATEYTTSIDIWSAGCVLAEL 256
HvGSK1/2/3/4        ..........NVLVDPLTHQVKICDFGSAKVLVPGEPNIAYICSRYYRAPELIFGATEYTTSIDIWSAGCVLAEL 255
ZmGSK1/2/3/4-1      SSELVSSSLFDVQVDPLTHQVKLCDFGSAKVLIPGEPNISYICSRYYRAPELIFGATEYTSSIDIWSAGCVLAEL 286
ZmGSK1/2/3/4-2      ..........NVLVDPLTHQVKLCDFGSAKVLIPGEPNISYICSRYYRAPELIFGATEYTTSIDIWSAGCVLAEL 261
SbGSK1/2/3/4        ..........NLLVDPLTHQVKICDFGSAKMLVKGEANISYICSRYYRAPELIFGATEYTTSIDIWSAGCVLAEL 271
Consensus                        vdplthqvk cdfgsak l  ge ni yicsryyrapelifgateyt sidiwsagcvlael

OsGSK1              LLGQPLFPGESAVDQLVEIIKVLGTPTREEIRCMNPNYTEFKFPQIKAHPWHKIFHKRMPPEAIDLASRLLQYSP 138
OsGSK2              LLGQPLFPGESAVDQLVR......................................................... 275
OsGSK3              LLGQPLFPGESAVDQLVEIIKVLGTPTREEIRCMNPNYTEFRFPQIKAHPWHKVFHKRMPPEAIDLASRLLQYSP 332
OsGSK4              LLGQPLFPGESAVDQLVEIIKVLGTPTREEIRCMNPNYTEFKFPQIKACPWHKIFHKRMPPEAIDLVSRLLQYSP 340
TaGSK1/2/3/4-1A     LLGQPLFPGESAVDQLVEIIKVLGTPTREEIRCMNPNYTEFRFPQIKAHPWHKVFHKKMPPEAIDLASRLLQYSP 332
TaGSK1/2/3/4-1B     LLGQPLFPGESAVDQLVEIIKVLGTPTREEIRCMNPNYTEFRFPQIKAHPWHKVFHKKMPPEAIDLASRLLQYSP 332
TaGSK1/2/3/4-1D     LLGQPLFPGESAVDQLVEIIKVLGTPTREEIRCMNPNYTEFRFPQIKAHPWHKVFHKKMPPEAIDLASRLLQYSP 332
TaGSK1/2/3/4-3A     LLGQPLFPGETAVDQLVEIIKVLGTPTREEIRCMNPNYTEFRFPQIKAHPWHKIFHKRMPAEAIDLASRLLQYSP 308
TaGSK1/2/3/4-3D     LLGQPLFPGETAVDQLVEIIKVLGTPTREEIRCMNPNYTEFRFPQIKAHPWHKIFHKRMPAEAIDLASRLLQYSP 331
HvGSK1/2/3/4        LLGQPLFPGETAVDQLVEIIKVLGTPTREEIRCMNPNYTEFRFPQIKAHPWHKIFHKRMPAEAIDLASRLLQYSP 330
ZmGSK1/2/3/4-1      LLGQPLFPGESAVDQLVEIIKVLGTPTREEIRCMNPNYTEFRFPQIKAHPWHKIFHKRMPPEAIDLASRLLQYSP 361
ZmGSK1/2/3/4-2      LLGQPLFPGESAVDQLVEIIKVLGTPTREEIRCMNPNYTEFRFPQIKAHPWHKIFHKRMPPEAIDLASRLLQYSP 336
SbGSK1/2/3/4        LLGQPLFPGESAVDQLVEIIKVLGTPTREEIRCMNPNYTEFRFPQIKAHPWHKIFHKRMPPEAIDLASRLLQYSP 346
Consensus           llgqplfpge avdqlv

OsGSK1              SLRCTALDACAHSFFDELREPNARLPNGRPFPPLFNFKHELASASPELIHRLIPDHIRRQHGLNFAHAGS      208
OsGSK2              ......................................................................      275
OsGSK3              SLRCTALDACAHPFFDELREPNARLPNGRPFPPLFNFKHELANSSQELISRLIPEHVRRQATHNFFNTGS      402
OsGSK4              NLRCTALEACAHSFFDELREPHAKLPNGRPFPPLFNFKQELANTHPELVSRLLPEHAQRHSGF.......      403
TaGSK1/2/3/4-1A     SLRCTALDACAHPFFDELWEPNARLPNGRPFPPLFNFKHELANASQDLINRLVPEHVRRQAGLAFVHAGS      402
TaGSK1/2/3/4-1B     SLRCTALDACAHPFFDELREPNARLPNGRPFPPLFNFKHELANASQDLINRLVPEHVRRQAGLAFVHAGS      402
TaGSK1/2/3/4-1D     SLRCTALDACAHPFFDELREPNARLPNGRPFPPLFNFKHELANASQDLINRLVPEHVRRQAGLAFVHAGS      402
TaGSK1/2/3/4-3A     NLRCTALDACAHSFFDELREPNARLPNGRPFPPLFNFKPELANASPELINRLVPEHVRRQNGLNFAHAGS      378
TaGSK1/2/3/4-3D     NLRCTALDACAHSFFDELREPNARLPNGRPFPPLFNFKPELANASPELINRLVPEHVRRQNGPNFAHAGS      401
HvGSK1/2/3/4        NLRCTALDACAHSFFDELREPNARLPNGRPFPPLFNFKPELANASPELINRLVPEHVRRQNGLNFAHAGS      400
ZmGSK1/2/3/4-1      SLRCSALDACAHPFFDELRTPNARLPNGRPFPPLFNFKHELANASPDLVSRLVPEHIRRQIGVNFGSS..      429
ZmGSK1/2/3/4-2      SLRCSALDACAHPFFDELRAPNARLPNGRPFPPLFNFKHELANASPDLINRLVPEQIRRQNGVNFGHTGS      406
SbGSK1/2/3/4        NLRCTALEACAHPFFDELREPHARLPNGRPFPPLFNFKQELANASPELINRLIPDHARRHLGLTLLPTTG      416
Consensus
```

Supplemental Figure S2-7 Alignment of multiple BZR1 proteins in rice, *T. aestivum*, *H. vulgare*, *Z. mays* and *S. bicolor*.

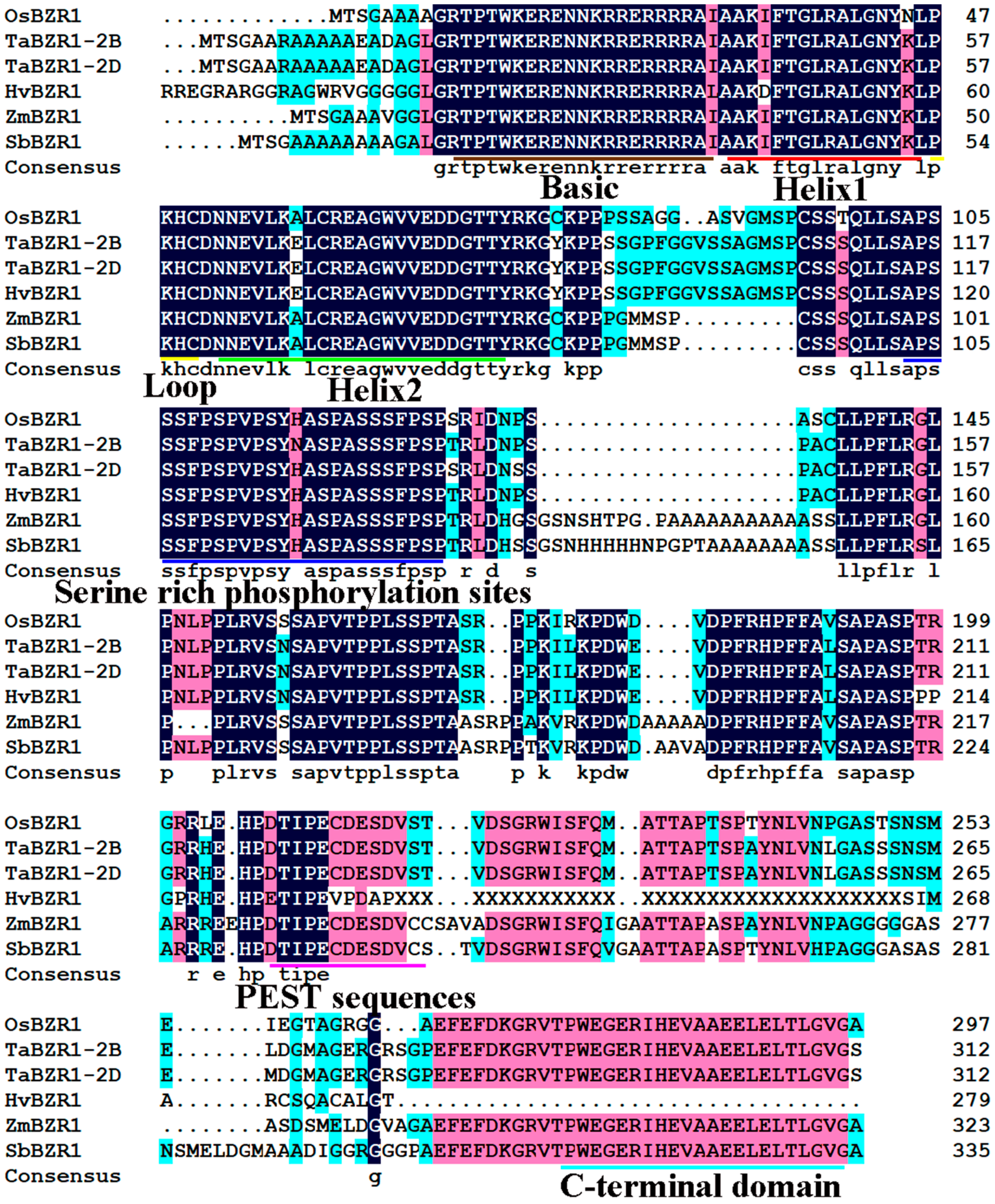

Supplemental Figure S2-8 Alignment of multiple SPY proteins in rice, *T. aestivum*, *H. vulgare*, *Z. mays* and *S. bicolor*.

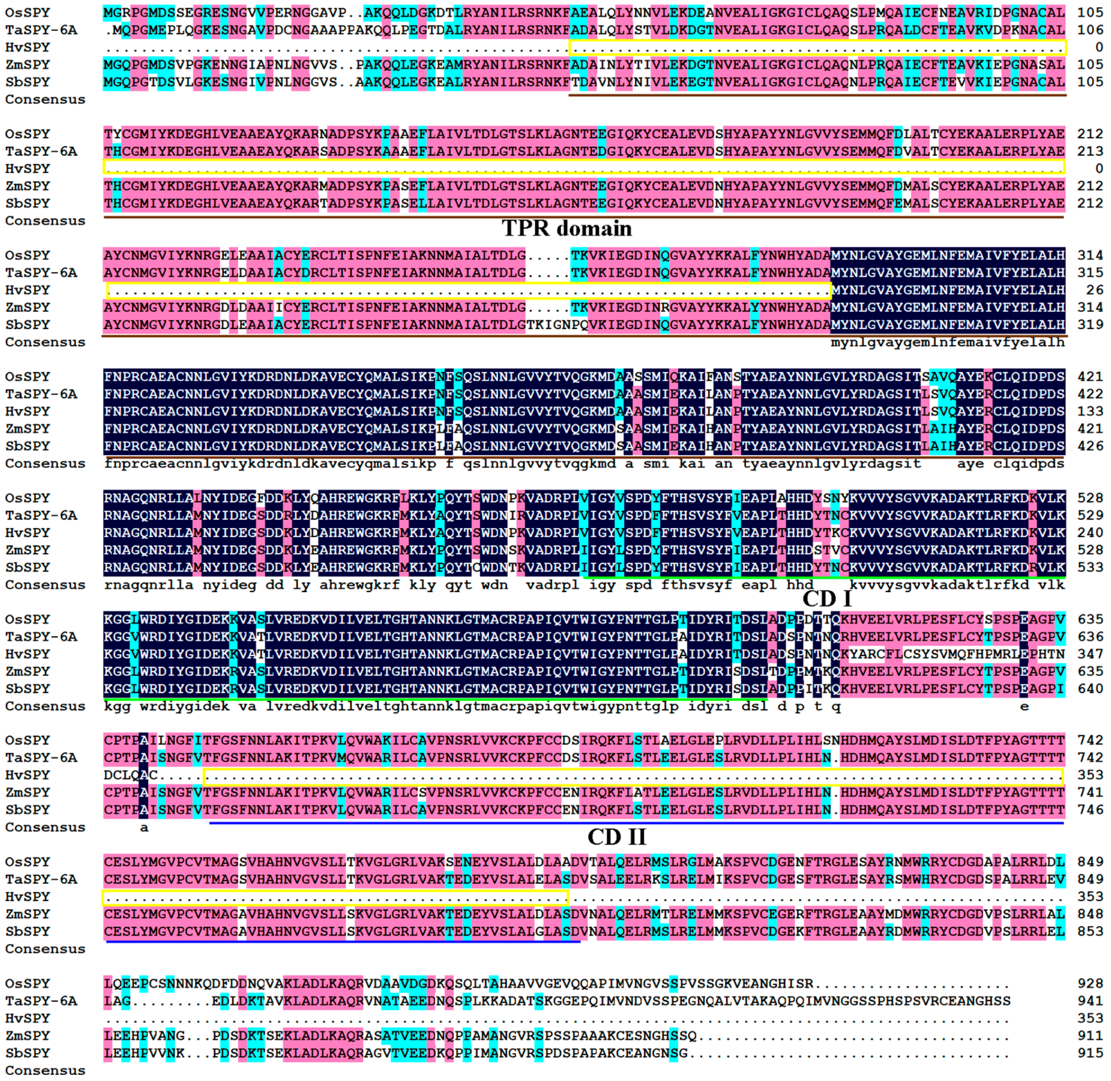

Supplemental Figure S2-9 Alignment of multiple GSR1 proteins in rice, *T. aestivum*, *H. vulgare*, *Z. mays* and *S. bicolor*.

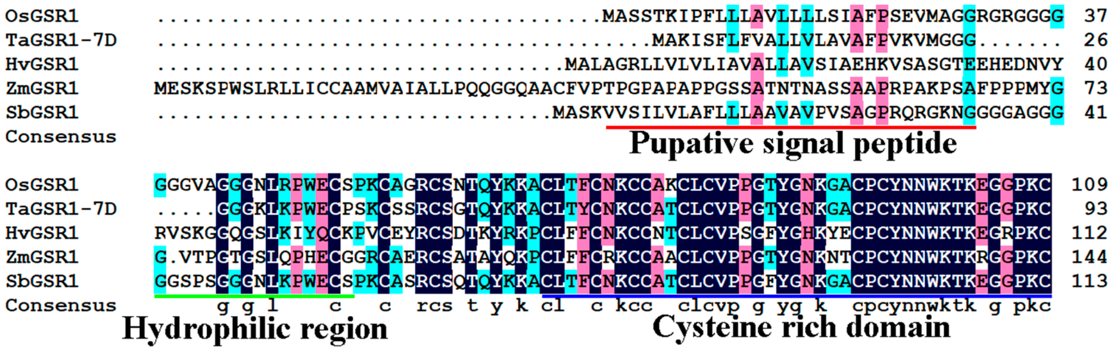

Supplemental Figure S2-10 Alignment of multiple ELT1 proteins in rice, *T. aestivum*, *H. vulgare*, *Z. mays* and *S. bicolor*.

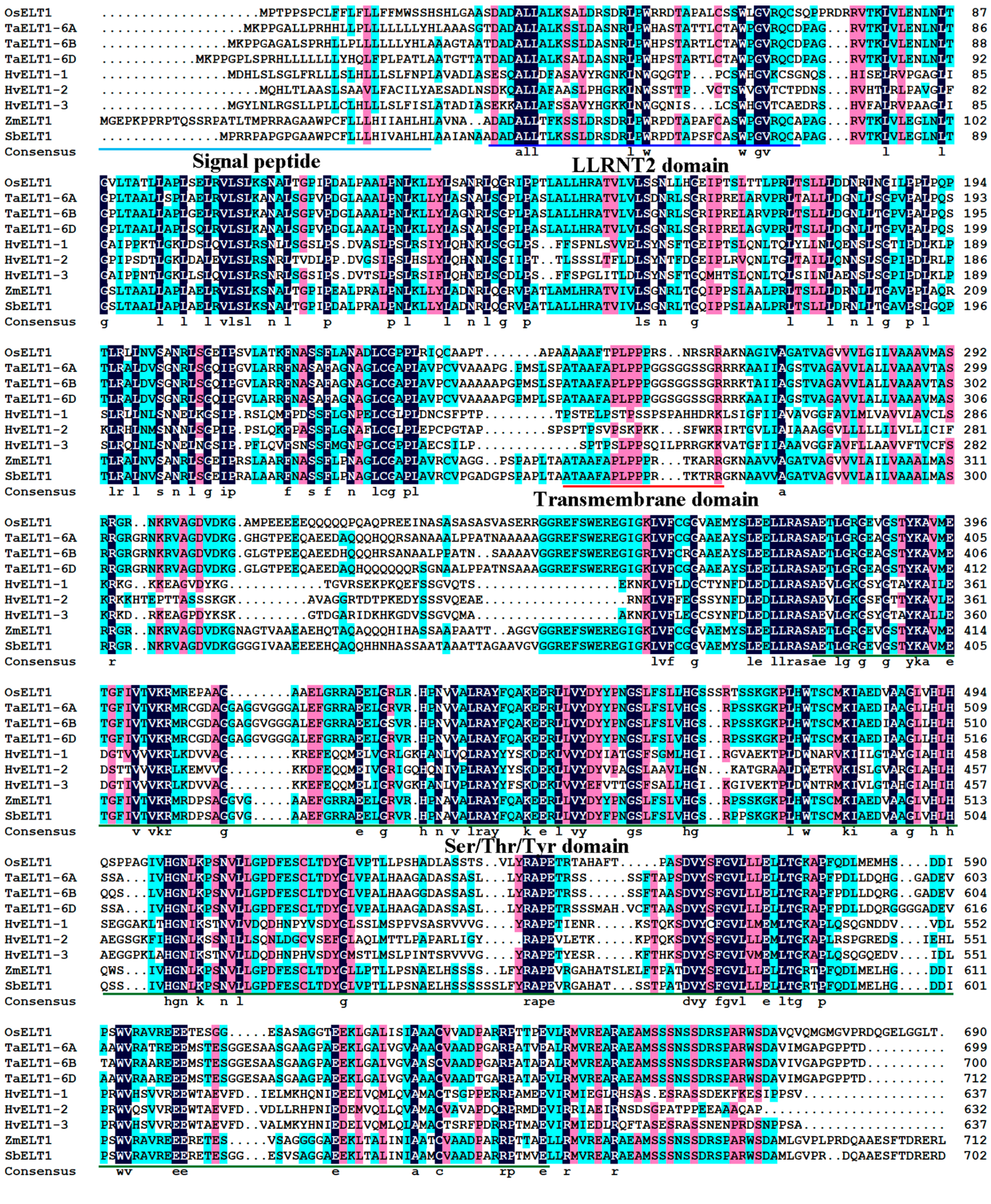

Supplemental Figure S2-11 Alignment of multiple SMOS1 proteins in rice, *T. aestivum*, *H. vulgare*, *Z. mays* and *S. bicolor*.

```
OsSMOS1     ............................................................................  0
TaSMOS1-1A  ............................................................................  0
TaSMOS1-1D  ............................................................................  0
HvSMOS1     ............................................................................  0
ZmSMOS1     MPRPRIPLRPRPPVSPVPAHWAPRVPIAYRPDPAAPPPPCFFHTISNPTTTHAAPKRSPVLRLAWLRCAAHTEP  74
SbSMOS1     ............................................................................  0
Consensus

OsSMOS1     ........MASPGPAAGMQQKLEAAAAAAGGGDGAEWGRGMQKMEAVGAGGEGVGAGAEQVAPPPRRPVAARKE  66
TaSMOS1-1A  ..................MASPNPAAADAAGLQKKMEAAAGGSEGPPPAY....GAAVAEQAPPRRLAPARKE  51
TaSMOS1-1D  ..................MASPNPAAADAAGLQKKMEAAAGGSEGPPPAYGAVVGVVVAEQAPPRRLAPARKE  55
HvSMOS1     ............................................................................  0
ZmSMOS1     LAVAARLGSAPTDRSIRPAVGAMASPNPEAAGLQAVAVAGAGEGGSSSSLSAVAGAAALSGELVPRRALALRKE 148
SbSMOS1     .................MASPNPEAAAGLQTVAVAAGGGEGGSSSSLGAVAGAAAVSSSGELVPRRSLAVRKE  56
Consensus

OsSMOS1     RVCTAKERISRMPPCAAGKRSSIYRGVTRHRWTGRYEAHLWDKSTWNQNQNKKGKQVYLGAYDDEEAAARAYDL 140
TaSMOS1-1A  RVCTAKDRISRMTPCAAGKRSSIYRGVTRHRWTGRYEAHLWDKSTWNQNQNKKGKQVYLGAYDDEEAAARAYDL 125
TaSMOS1-1D  RVCTAKDRISRMTPCAAGKRSSIYRGVTRHRWTGRYEAHLWDKSTWNQNQNKKGKQVYLGAYDDEEAAARAYDL 129
HvSMOS1     ............................................................................  0
ZmSMOS1     RVCTAKERISRMPPCAAGKRSSIYRGVTRTDFG..............................PSIGIGGQVDMRLTFG 197
SbSMOS1     RVCTAKERISRMPPCAAGKRSSIYRGVTRHRWTGRYEAHLWDKSTWNQNQNKKGKQVYLGAYDDEEAAARAYDL 130
Consensus
              AKER domain                      AP2-R1 domain

OsSMOS1     AALKYWGAGTQINFPVSDYARDLEEMQMISKEDYLVSLRRKSSAFSRGLPKYRGLPRQLHNSRWDASLGHLLGN 214
TaSMOS1-1A  AALKYWGAGTQINFPVSDYTRDVEEMQMISKEDYLVSLRRKSSAFSRGLPKYRGLPRQLHNSRWDASLGQLLGN 199
TaSMOS1-1D  AALKYWGAGTQINFPVSDYTRDVEEMQMISKEDYLVSLRRKSSAFSRGLPKYRGLPRQLHNSRWDASLGQLLGN 203
HvSMOS1     .........................MQMISKEDYLVSLRRKSSAFSRGLPKYRGLPRQLHNSRWDASLGQLLGN  49
ZmSMOS1     TKARGIRIRTKRANRVSDYARDLEEMQMISKEDYLVSLRRKSSAFYRGLPKYRGLLRQLHNSRWDTSLG..LGN 269
SbSMOS1     AALKYWGAGTQINFPVSDYARDLEEMQMISKEDYLVSLRRKSSAFYRGLPKYRGLLRQLHNSRWDTSLG..LGN 202
Consensus                            mqmiskedylvslrrkssaf rglpkyrgl rqlhnsrwd slg  lgn
                                          Linker region

OsSMOS1     DYMSLG..KDITLDGKFAGTFGLERKIDLTNYIRWWLPKKTRQSDTSKMEEVTDEIRAIESSMQRTEPYKFPSL 286
TaSMOS1-1A  DYMNLSCGKGIALDGKFAGSFGLERKIDLTNYIRWWVPKKTRQSDTSKAEEVADEIRAIEGSVQLTEAYKLPTL 273
TaSMOS1-1D  DYMNLSCGKGIALDGKFAGSFGLERKIDLTNYIRWWVPKKTRQSDISKAEEVADEIRAIEGSVQLTEPYKLPSL 277
HvSMOS1     DYMNLSCGKGIALDGKFAGSFGLERKIDLTNYIRWWVPKKARQSDTSKAEEVADEIRAIEGSVQLTEPYKLPSL 123
ZmSMOS1     DYMSLSCGKDIMLDGKFAGSFGLERKIDLTNYIRWWLPKKTRQSDTSKTEEIADEIRAIESSMQQTEPYKLPSL 343
SbSMOS1     DYMSLSCGKDIMLDGKFAGSFGLERKIDLTNYIRWWLPKKTRQSDTSKTEEIADEIRAIESSMQQTEPYKLPSL 276
Consensus   dym l   k i ldgkfag fglerkidltnyirww pkk rqsd sk ee  deiraie s q te yk p l
                     AP2-R2 domain                                    EPY domain

OsSMOS1     GLHSNSKPSSVVLSACDILSQSDAFKSFSEKSTKLSEECTFSKEMDEGKTVTPVPATGHDTTAVNMNVNGLLVQ 360
TaSMOS1-1A  GLGSHSNPSSAGLSACSILSQSGAFKSFLEKSTKLSEECTFSKEIDEGKVVVSVPTTGHHTSPVDINMNGLLVQ 347
TaSMOS1-1D  GLGSHSNPSSAGLSACSILSQSGAFKSFLEKSTKLSEECTFSKEIDEGKGVVSVPTTGHHTSPVDINMNGLLVQ 351
HvSMOS1     GLGSHSKPSSAGLSACGILSQSGAFKSFLEKSTKLSEECTFSKEIDEGKVAVSEPTTGHHTSAVDINMNGLLVQ 197
ZmSMOS1     GFSSPSKPSSMGLSACSILSQSDAFKSFLEKSTKLSEECSLSKEIVEGKTVASVPATGYDTGAININMNELLVQ 417
SbSMOS1     GLGSPSKPSSVGLSACSILSQSDAFKSFLEKSTKLSEECTLSKEIVEGKTVASVPATGYDTGAININMNELLVQ 350
Consensus   g  s s pss  lsac ilsqs afksf ekstklseec  ske  egk     p tg  t     n n llvq
                         LSFE domain

OsSMOS1     RAPYTLPSVTAQ.MKNTWNPADPSADPLFWTNFILPASQPVTMATIATTTFAKNEVSSSDPFHGQE....    425
TaSMOS1-1A  RAPYTLAPVMPTPMKSTWSPADPSADHLFWSNFILPSSQPVTMATITTTTFAKNEVSSSDPFKSQEYEHT    417
TaSMOS1-1D  RAPYTLAPVMPTPMKSTWSPADPSADHLFWSNFILPSSQPVTMATITTTTFAKNEVSSSDPFKNQEYEHT    421
HvSMOS1     RAPYTLAPVMPT.MKSTWSPSDPSADHLFWSNFILPSSQPVTMATITTTTFAKNEVSSSDPFKNQEYEHT    266
ZmSMOS1     RSTYSMAPVMPTPMKSTWSPADPSVDPLFWSNFVLPSSQPVTMATITTTTFAKNEVSSSDPFQSQE....    483
SbSMOS1     RSTYSMAPVMPTPMKTTWSPADPSVDPLFWSNFVLPSSQPVTMATITTTTFTKNEVSSSDPFQSQE....    416
Consensus   r  y    v    mk tw p dps d lfw nf lp sqpvtmati tttf knevsssdpf  qe
                             WTF domain
```

Supplemental Figure S2-12 Alignment of multiple DLT proteins in rice, *T. aestivum*, *H. vulgare*, *Z. mays* and *S. bicolor*.

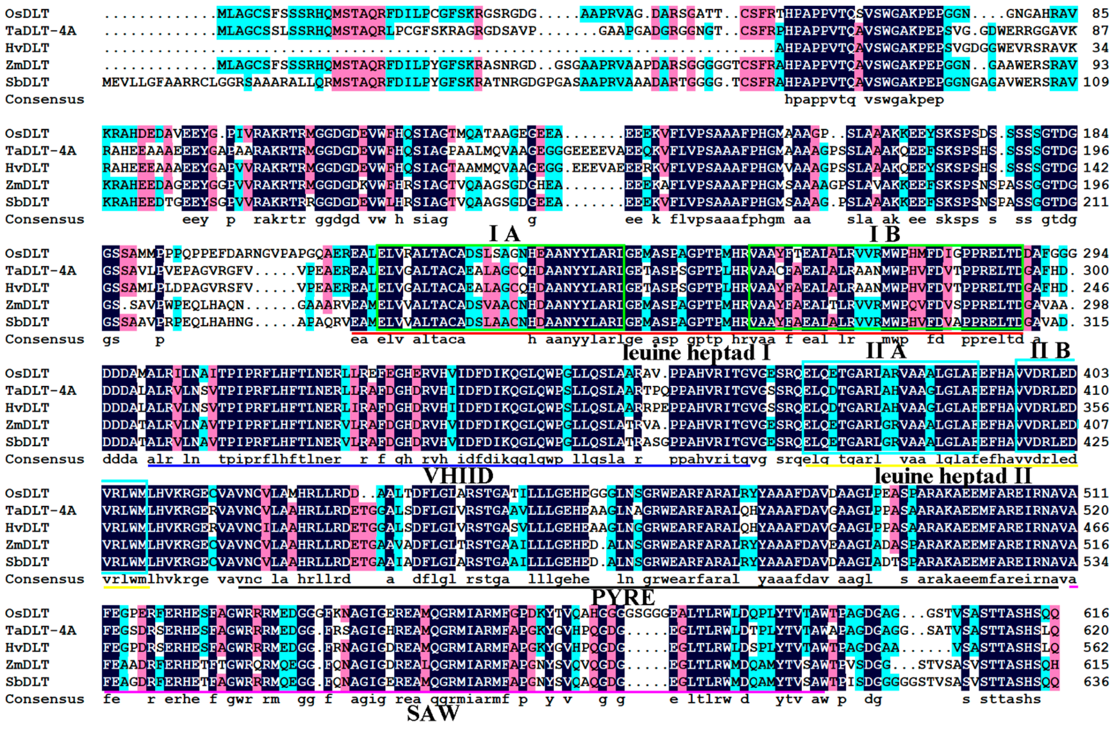

Supplemental Figure S2-13 Alignment of multiple OFP1 proteins in rice, *T. aestivum*, *H. vulgare*, *Z. mays* and *S. bicolor*.

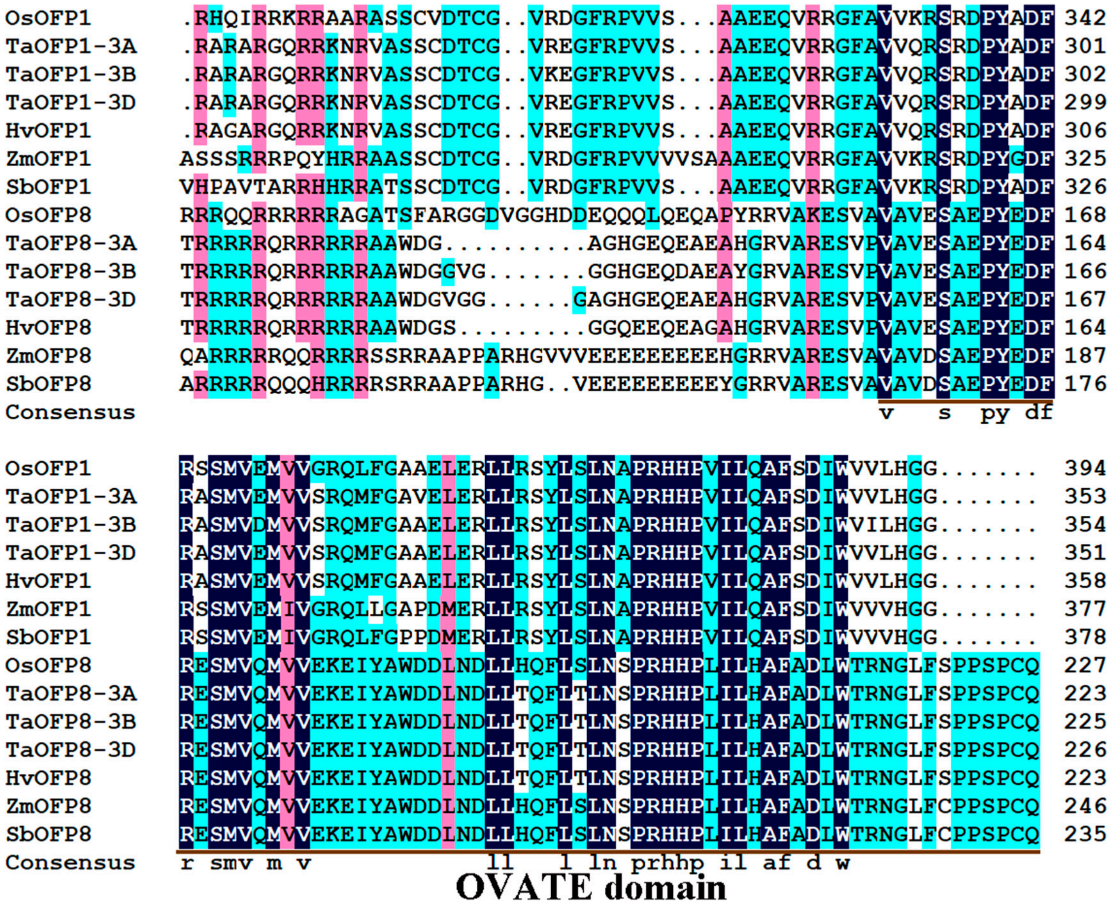

Supplemental Figure S2-14 Alignment of multiple LIC proteins in rice, *T. aestivum*, *H. vulgare*, *Z. mays* and *S. bicolor*.

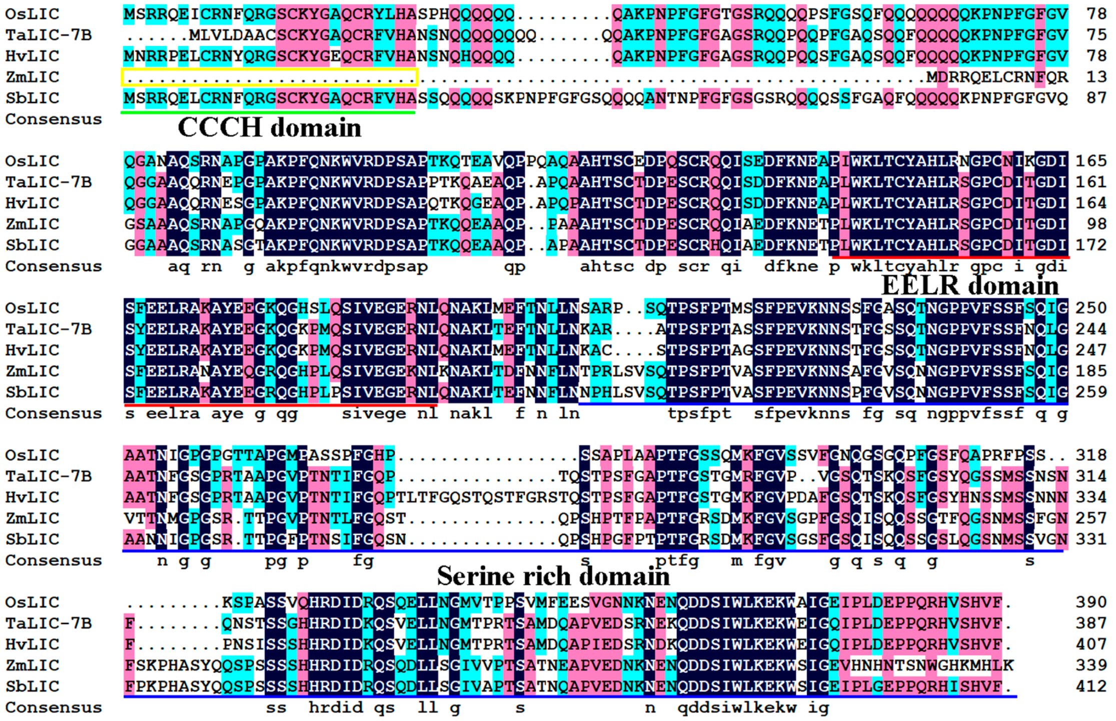

Supplemental Figure S2-15 Alignment of multiple CYC U4:1 proteins in rice, *T. aestivum*, *H. vulgare*, *Z. mays* and *S. bicolor*.

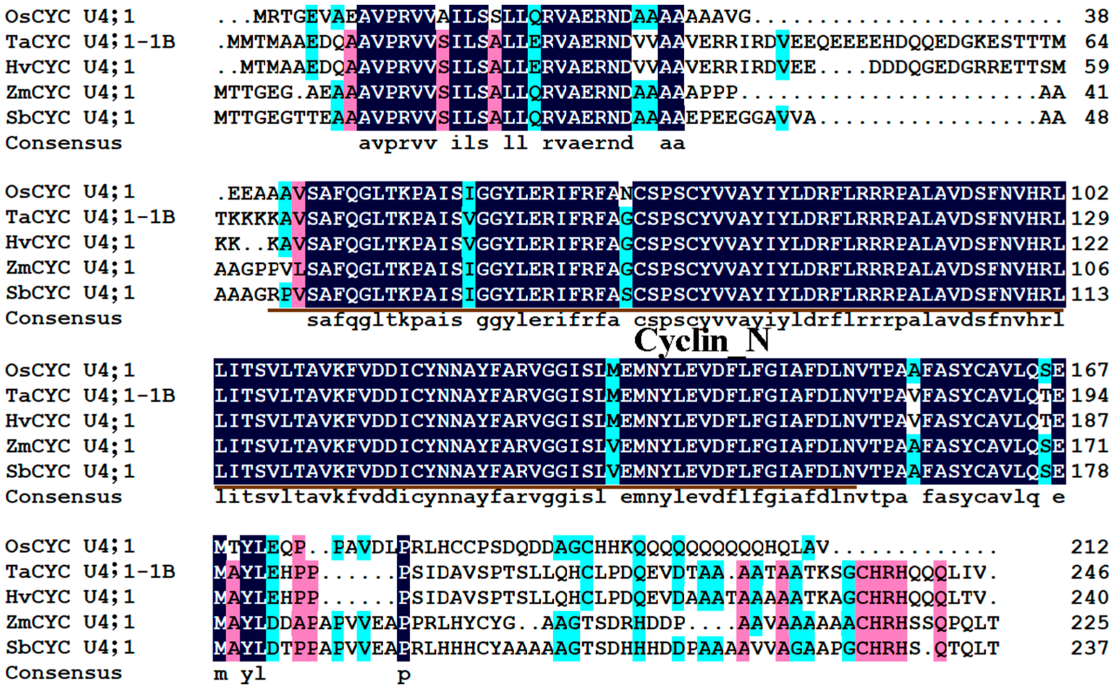

Supplemental Figure S2-16 Alignment of multiple TUD1 proteins in rice, *T. aestivum*, *H. vulgare*, *Z. mays* and *S. bicolor*.

```
OsTUD1      MPQYQELPCGGQVLDIDTALKDGILGGGPELGDAAAGDGGKQPVELRKMMDELDAAGDGGG.DEAV  65
TaTUD1-4B   MPQYQELPCGGQVLDIDAALKDGILGCGPEPGDGALGDGGKQPVELRKMMDELDAAGDGGG.DEAV  65
TaTUD1-4D   MPQYQELPCGGQVLDIDAALKDGILGCGPEPGDGALGDGGKQPVELRKMMDELDAAGDGGG.DEVV  65
HvTUD1      MPQYQELPCGGQVLDIDTALKDGILGCGPEPGDGALGDGGKQPVELRKMMDELDAAGDAGGGDEVV  66
ZmTUD1      MPQYQELPCGGQVLDIDTALKDGILGGALEPEDAAAGDAGKQPVELRKMMDELDAAGDGGG.DEAV  65
SbTUD1      MPQYQELPCGGQVLDIDTALKDGILGGALEPVDAAAGDAGKQPLELRKMMDELDAAGDGGG.DEAV  65
Consensus   mpqyqelpcggqvldid alkdgilg   e  d a gd gkqp elrkmmdeldaagd gg de v

OsTUD1      PAVFICPISLEPMVDPVTLCTGQTYESANISRWLALGHRTCPTTMQELWDVTPIPNTTLRQLIAAW 131
TaTUD1-4B   PAVFICPISLEPMVDPVTLCTGQTYERANISRWLALGHRTCPTTMQELWDDALTPNATLRQLIAAW 131
TaTUD1-4D   PAVFICPISLEPMVDPVTLCTGQTYERANISRWLALGHRTCPTTMQELWDDALTPNATLRQLIAAW 131
HvTUD1      PAVFICPISLEPMVDPVTLCTGQTYERANISRWLALGHRTCPTTMQELWDDALTPNATLRQLIAAW 132
ZmTUD1      PAVFICPISLEPMVDPVTLCTGQTYERANISRWLALGHRTCPTTMQELWDDALTPNATLRQLIAAW 131
SbTUD1      PAVFICPISLEPMVDPVTLCTGQTYERANISRWLALGHRTCPTTMQELWDDALTPNATLRQLIAAW 131
Consensus   pavficpislepmvdpvtlctgqtye anisrwlalghrtcpttmqelwd    pn tlrqliaaw
                                 U-box domain

OsTUD1      FSRRYTRFKKRSADFHGRAAELVHALRGTAVPKRQPLKGQARVAALRELRSLAAAHQSVTKAIAEA 197
TaTUD1-4B   FSRRYTRFKKRSADYHGRAADLVHGLRGTAVPRRQPLKGQARVAALRELRSLASNHQSVTKAIAEA 197
TaTUD1-4D   FSRRYTRFKKRSADYHGRAADLVHGLRGTAVPRRQPLKGQARVAALRELRSLASNHQSVTKAIAEA 197
HvTUD1      FSRRYTRFKKRSADYHGRAADLVHGLRGTAVPRRHPLKGQARVAALRELRSLASAHQSVTKAIAEA 198
ZmTUD1      FSRRYTRFKKRSADFHGRAADLVHGLRGTAMPRRQALKGQARVAALRELRALATAHQSVTKAIAEA 197
SbTUD1      FSRRYTRFKKRSADFHGRAADLVHSLRGTAVPRRQPLKGQARVAALRELRTLATTHQSVTKAIAEA 197
Consensus   fsrrytrfkkrsad hgraa lvh lrgta p r  lkgqarvaalrelr la  hqsvtkaiaea

OsTUD1      GGVGLLTSLLGPFTSHAVGSEAVAILVSGVPLDADAKAALMQPAKVSLLVDMLNEGAVDTKINCVR 263
TaTUD1-4B   GGVSLLTSLLGPFTSHSVGSEAVAILVSGVPLDADAKAALMQPAKVSLVVDMLNEGAVDTKINCVR 263
TaTUD1-4D   GGVSLLTSLLGPFTSHSVGSEAVAILVSGVPLDADAKAALMQPAKVSLVVDMLNEGAVDTKINCVR 263
HvTUD1      GGVSLLTSLLGPFTSHSVGSEAVAILVSGVPLDGDAKAALMQPAKVSLVVDMLNEGAVDTKINCVR 264
ZmTUD1      GGVVLLTSLLGPFTSHAVGTEAVAILVSGVPLDADTKAALMQPAKVSLVVDMLNEGAADTKINCVR 263
SbTUD1      GGVALLTSLLGPFTSHAVGTEAVAILVSGVPLDVDAKAALMQPAKVSLVVDMLNEGAAETKINCVR 263
Consensus   ggv lltsllgpftsh vg eavailvsgvpld d kaalmqpakvsl vdmlnega  tkincvr

OsTUD1      LIRILMEEKGFRPDTVASLSLIVGVMRLVRDKRHPDGVAAGLELLNSICAVHKPARSLIVSIGAVP 329
TaTUD1-4B   LIRILMEEKGFRPETVASLSLIAGSMRLVRDKRHQDGVAAGLELLNSICAVHRPARSMIVSIGAVQ 329
TaTUD1-4D   LIRILMEEKGFRPETVASLSLIAGSMRLVRDKRHQDGVAAGLELLNSICAVHRPARSMIVSIGAVQ 329
HvTUD1      LIRILMEEKGFRPETVASLSLIVGAMRLVRDKRHPDGVAAGLELLNSICAVHRPARSMIVSIGAVQ 330
ZmTUD1      LIRILMDERGFRPETVASLSLIVGVMRLIRDKRHPDGVVAGLELLNSICAVHRPARSMVVSIGAVP 329
SbTUD1      LIRILMDERGFRPETVASLSLIVGVMRLIRDKRHPDGVVAGLELLNSICAVHRPARSMIVSIGAVP 329
Consensus   lirilm e gfrp tvaslsll g mrl rdkrh dgv aglellnsicavh pars  vsigav

OsTUD1      QLVELLPELPTECVEPALDILDALAAVPEGRIALKDCPRTITNAVRLLMRVSEACTRRALSMLWVV 395
TaTUD1-4B   QLVELLPELATECVEPALDILDALASVPEGMTALKDCPRTIPNAVRLLMRVSEACTQRALSMLWVV 395
TaTUD1-4D   QLVELLPELATECVEPALDILDALASVPEGMTALKDCPRTIPNAVRLLMRVSEACTQRALSMLWVV 395
HvTUD1      QLVELLPELATECVEPALDILDALASVPEGRTALKDCPRTIPNAVRLLMRVSEACTQRALSMLWVV 396
ZmTUD1      QLVELLPELATECVEPALDILDALSAVPEGRTALKDCPRTIPNAVRVLMRVSEACTRRALSMLWTV 395
SbTUD1      QLVELLPELATECVEPALDILDALSAVPEGRVALKDCPRTIPNAVRLLMRVSEACTRRALSMLWTV 395
Consensus   qlvellpel tecvepaldildal  vpeg  alkdcprti navr lmrvseact ralsmlw v

OsTUD1      CRMAPEECAPAALDAGLGAKLLLVIQSGCGPELKQQASELLKLCTMNCTSTVFISKCKLTKTI    458
TaTUD1-4B   CRMVPDESAPAALEVGLAAKLLLVIQSGCGPELKQQASELLKLCTMHCTSTVFLAKCKLTKTI    458
TaTUD1-4D   CRMVPEESAPAALEVGLAAKLLLVIQSGCGPELKQQASELLKLCTMHCTSTVFLAKCKLTKTI    458
HvTUD1      CRMVPEESAPAALEVGLAAKLLLVIQSGCGPELKQQASELLKLCTVNCTSTVFLAKCKLTKTI    459
ZmTUD1      CRMAPEECAPAAVEAGLAAKLLLVIQSGCAPELKQKASELLKLCRLNCTDTLFISKCKLTRTI    458
SbTUD1      CRMAPEECAPAAVEAGLAAKLLLVIQSGCAPELKQKASELLKLCRLNYTDTLFISKCKLTRTI    458
Consensus   crm p e apaa   gl aklllviqsgc pelkq asellklc    t t f  kcklt ti
```

Supplemental Figure S2-17 Alignment of multiple D1 proteins in rice, *T. aestivum*, *H. vulgare*, *Z. mays* and *S. bicolor*.

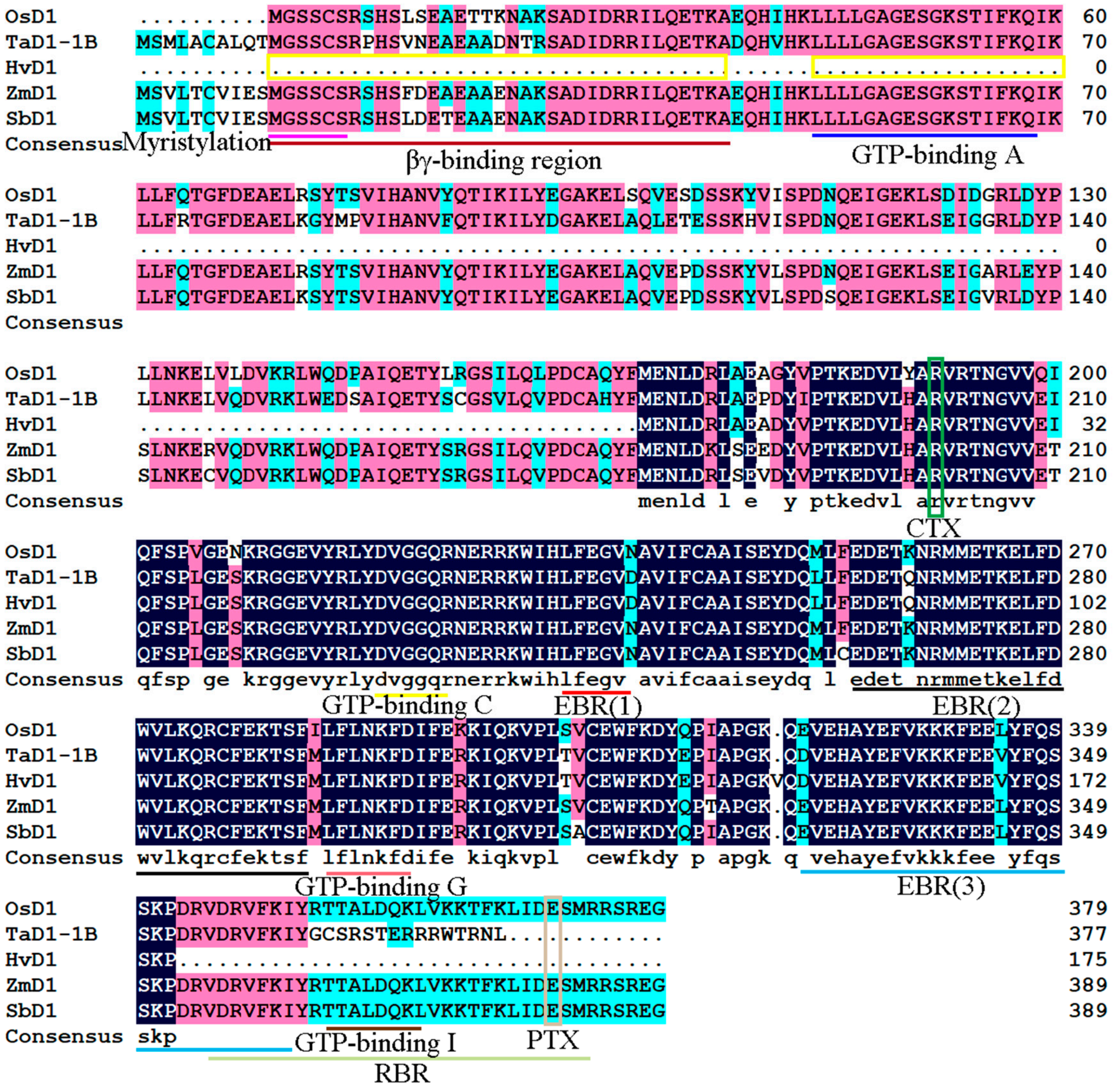

Supplemental Figure S2-18 Alignment of multiple BU1, ILI1 and BUL1 proteins in rice, *T. aestivum*, *H. vulgare*, *Z. mays* and *S. bicolor*.

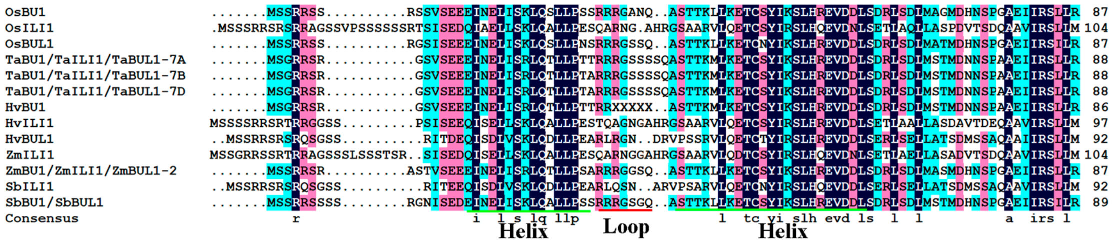

Supplemental Figure S2-19 Alignment of multiple IBH1 proteins in rice, *T. aestivum*, *H. vulgare*, *Z. mays* and *S. bicolor*.

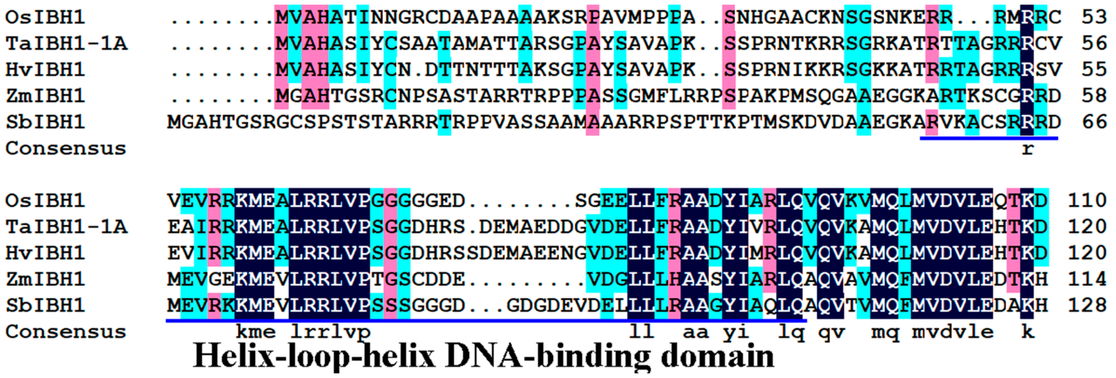

Supplemental Figure S2-20 Alignment of multiple GRAS19 proteins in rice, *T. aestivum*, *H. vulgare*, *Z. mays* and *S. bicolor*.

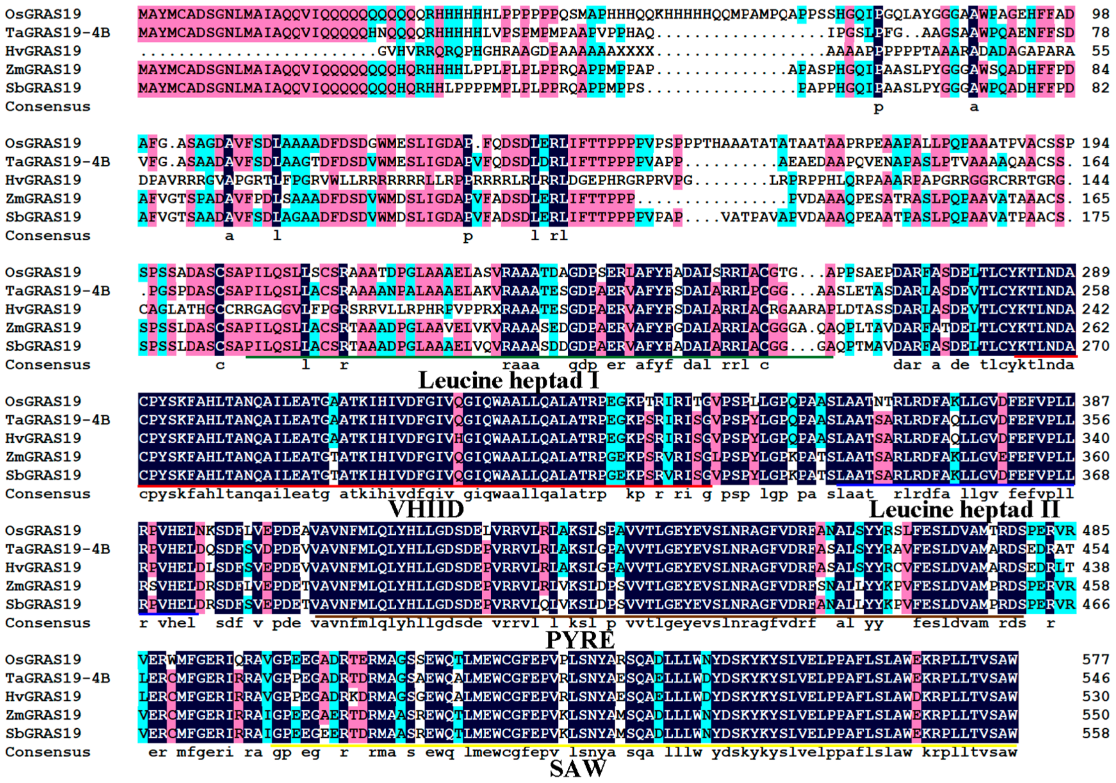

Supplemental Figure S2-21 Alignment of multiple MADS22, MADS47 and MADS55 proteins in rice, *T. aestivum*, *H. vulgare*, *Z. mays* and *S. bicolor*.

```
OsMADS22              ...................MARERREIKRIESAAARQVTFSKRRRGLFKK   31
OsMADS47              MAGGGGGGGRGEGEGRAATGKRERIAIRRIDNLAARQVTFSKRRRGLFKK   50
OsMADS55              ...................MARERREIRRIESAAARQVTFSKRRRGLFKK   31
TaMADS22/47/55-4A     ..................................................    0
TaMADS22/47/55-4B     .................MAGKRERIAIRRIENLAARQVTFSKRRRGLFKK   33
TaMADS22/47/55-4D     .................MAGKRERIAIRRIENLAARQVTFSKRRRGLFKK   33
HvMADS22              ...................MARERREIKRIESAAARQVTFSKRRRGLFKK   31
HvMADS47              .................MAGKRERIAIRRIENLAARQVTFSKRRRGLFKK   33
HvMADS55              ..................................................    0
ZmMADS22/47/55-1      ...................MARERREIKRIESAAARQVTFSKRRRGLFKK   31
ZmMADS22/47/55-2      ...................MARERREIKRIESAAARQVTFSKRRRGLFKK   31
SbMADS22              ...................MARERREIKRIESAAARQVTFSKRRRGLFKK   31
SbMADS47              .................MAGKRERIAIRRIDNLAARQVTFSKRRRGLFKK   33
SbMADS55              ...................MARERREIRRIENAAARQVTYSKRRRGLFKK   31
Consensus
                         Amino-terminal (N)

OsMADS22              AEELSVLCDADVALIVFSSTGKLSHFASSSMNEIIDKYNTHSNNLGKAE.   80
OsMADS47              AEELSILCDAEVGLVVFSATGKLFQFASTSMEQIIDRYNSHSKTLQRAEP  100
OsMADS55              AEELAVLCDADVALVVFSSTGKLSQFASSNMNEIIDKYTTHSKNLGKTDK   81
TaMADS22/47/55-4A     ..............................MNQIIDRYNSHSKILKKADE   20
TaMADS22/47/55-4B     AGELSILCDAEVGLAVFSATGKLFQFASSSMNQIIDRYNSHSKILKKADE   83
TaMADS22/47/55-4D     AEELSILCDAEVGLAVFSATGKLFQFASSSMNQIIDRYNSHSKILKKADE   83
HvMADS22              AEELSVLCDADVALIVFSSTGKLSQFASSSMNEIIDKYSTHSKNLGKTD.   80
HvMADS47              AEELSILCDAEVGLAVFSATGKLFQFASSSMNQIIDRYNSHSKILKKVDE   83
HvMADS55              ..............................MNEIIDKYSTHSKNLGKSDQ   20
ZmMADS22/47/55-1      AEELSVLCDADVALIVFSSTGKLSQFASSSMNEIIDKYSTHSKNLGKAE.   80
ZmMADS22/47/55-2      AQELSVLCDADVALIVFSSTGKLSQFASSSMNEIIDKYNTHSKNLGKTE.   80
SbMADS22              AEELSVLCDADVALIVFSSTGKLSQFASSSMNEIIDKYNTHSKNLGKAE.   80
SbMADS47              AEELSILCDAEVGLVVFSATGKLFHFASTSMKQVIDRYDSHSKNLQKSEA   83
SbMADS55              AEELAVLCDADVALVVFSATGKLSQFASTSMNHIIDKYSTHSKNLGKSHQ   81
Consensus                                           m   id y  hs  l
                                MADS domain

OsMADS22              QPSLDINLEHSKYAHLNEQLAEASLRLRQMRGEELEGLSIDELQQLEKNL  130
OsMADS47              SQLDLQGEDSSTCARLKEELAETSLRLRQMRGEELHRLNVEQLQELEKSL  150
OsMADS55              QPSIDINLEHSKCSSLNEQLAEASLQLRQMRGEELEGLSVEELQQMEKNL  131
TaMADS22/47/55-4A     PSQLDLHEDS.NCARLRDELAEASLWLQQMRGEELQSLNVQQLQALEKSL   69
TaMADS22/47/55-4B     PSQLDLHEDS.NCARLRDELAEASLWLQQMRGEELQSLNVQQLQALEKSL  132
TaMADS22/47/55-4D     PSQLDLHEDS.NCARLSDELAEASLWLQQMRGEELQSLNVQQLQALEKSL  132
HvMADS22              QPTLDINLEHSKYANLNDQLAEASLRLRQMRGEELEGLSVDELQQLEKNL  130
HvMADS47              PSQLDLHEDS.NCARLRDELAEASLWLQQMRGEELQSLNVQQLQALEKSL  132
HvMADS55              QPAIDINLEHCKYDSLNEQLAEASLRLRHMRGEELDGLSVGELQQMEKNL   70
ZmMADS22/47/55-1      QPSLDINLEHSKYANLNEQLVEASLRLRQMRGEELEGLSVEELQQLEKNL  130
ZmMADS22/47/55-2      QPSLDINLEHSKYANLNEQLAEASLRLRQMRGEELEGLNVEELQQLEKNL  130
SbMADS22              EPSLDINLEHSKYANLNEQLVEASLRLRQMRGEELEGLSVEELQQLEKNL  130
SbMADS47              PSQLQSHIDDGTCARLKQELAETSLKLRQMRGEELQRLSVQQLQELEKTL  133
SbMADS55              QSPIDINIEQSKYTGLNEQLAEATHGLRQMRGENLEGLSVEELHQMERKL  131
Consensus                            l   l e    l  mrge l  l    l   e  l
                                         K domain

OsMADS22              EAGLHRVMLTKDQQFMEQISELQRKSSQLAEENMQLRNQVSQISPAEKQV  180
OsMADS47              ESGLGSVLKTKSKKILDEIDGLERKRMQLIEENLRLKEQVSRMS.RMEEM  199
OsMADS55              EAGLQRVLCTKDQQFMQEISELQRKGIQLAEENMRLRDQMPQVPTAGLAV  181
TaMADS22/47/55-4A     ESGLGSVLKTKSQKIMDQISELERKRVQLIEENARLKEQASK.....MEM  114
TaMADS22/47/55-4B     ESGLGSVLKTKSQKIMDQISELERKRVQLIEENARLKEQLQAS...KMEM  179
TaMADS22/47/55-4D     ESGLGSVLKTKSQKIMDQISELENKRVQLIEENARLKEQASK.....MEM  177
HvMADS22              ETGLHKVLQTKDQQFLEQINELHRKSSQLAEENKKLRNQVAQVPTAGKLV  180
HvMADS47              ESGLSSVLKTKSQKIMDQISELEKKRVQLIEENARLKEQASK....MEMQ  178
HvMADS55              ETGLQRVLCTKDRQFMQQISDLQQKGTQLAEENMRLKNQMHEVPTASMVA  120
ZmMADS22/47/55-1      ESGLHRVLQTKDQQFLEQISDLEQKSTQLAEENRQLRNQVSHIPPVGKQS  180
ZmMADS22/47/55-2      ESGLHRVLQTKDQQFLEQINDLERKSTQLAEENMQLRNQVSQIPPAGKQA  180
SbMADS22              ETGLHRVLQTKDQQFLEQISDLERKSTQLAEENMQLRNQVTSLL......  174
SbMADS47              ESGLGSVLKTKSQKILDEISGLERKRMELIEENSRLKEQVTRMA.RMETQ  182
SbMADS55              EAGLHRVLSTKDQLFTQQISELQQKGTQLEDENRRLKEQMPQVLTAGTMV  181
Consensus             e gl  v  tk       i  l  k   l  en  l  q

OsMADS22              VDTENF.VTEGQSSESVMTALHSGSSQSQDNDDGSDVSLKLGLPCGAW    227
OsMADS47              QPGPDSEIVYEEGQSSESVTNASYPRPPPDNDYSSDTSLKLGLHS...    244
OsMADS55              PDTEN..VLTEDGQSSESVMTALNSGSSQDNDDGSDISLKLGLP....    223
TaMADS22/47/55-4A     QVAADSPVVYEEGQSSESVTNTSYPRPPLDTEDSSDTSLRLGLPLYNS    162
TaMADS22/47/55-4B     QVAADSPAVYEEGQSSESVTNTSYPRPPLDTEDSSDTSLRLGLPLYNS    227
TaMADS22/47/55-4D     QVAADSPVVYEEGQSSESVTNTSYPRPPLDTEDSSDTSLRLGLPLFNS    225
HvMADS22              VVDTEN.VIAEDGQSSESVMTALHSGSSQDNDDGSDVSLKLALPWK..    225
HvMADS47              VAADPLVVVYDEGQSSESVTNTSYPRPPLDTEDSSDTSLRLGLSLFNS    226
HvMADS55              VAD....VVPEDVHSSDSVMTAVHSASSQDNDDGSDISLKLALPWK..    162
ZmMADS22/47/55-1      VADAEN.VIAEDGQSSESVMTALHSGSSQDNDDGSDVSLKLG......    221
ZmMADS22/47/55-2      VADTEN.VIAEEGQSSESVMTALHSGSSQDNDDGSDVSLKLG......    221
SbMADS22              ................................................    174
SbMADS47              LG.VDSEIVYEEGQSSESVTNTSYPRPSTDTDDCSDTSLRLGLPFFSS    229
SbMADS55              VGAGAENILTEDGQSSESVMTALHSGSSLDNDDGSDICLKLSLP....    225
Consensus
                                 Carbon terminal
```

Supplemental Figure S2-22 Alignment of multiple XIAO proteins in rice, *T. aestivum*, *H. vulgare*, *Z. mays* and *S. bicolor*.

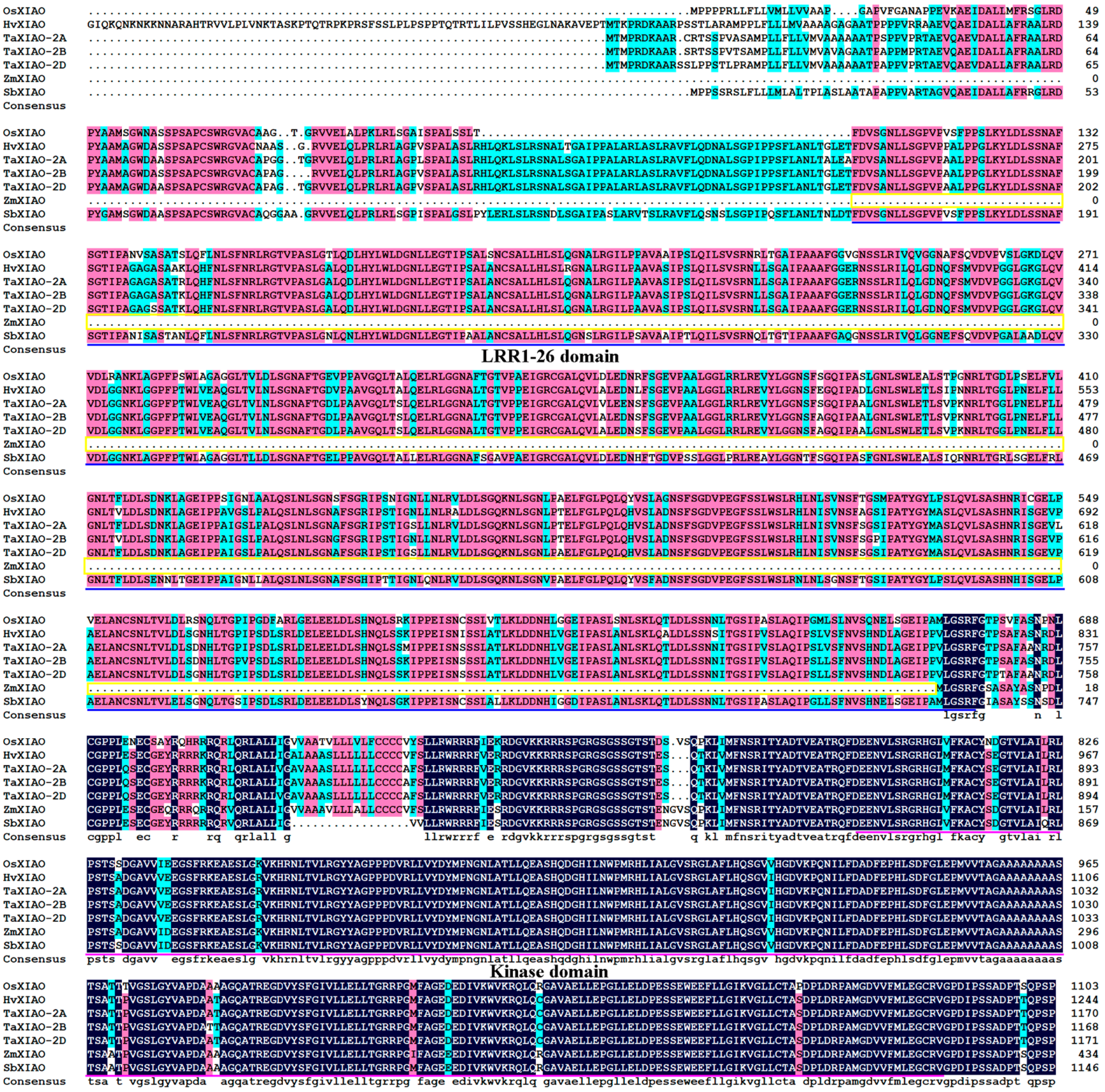

Supplemental Figure S2-23 Alignment of multiple LC2 proteins in rice, *T. aestivum*, *H. vulgare*, *Z. mays* and *S. bicolor*.

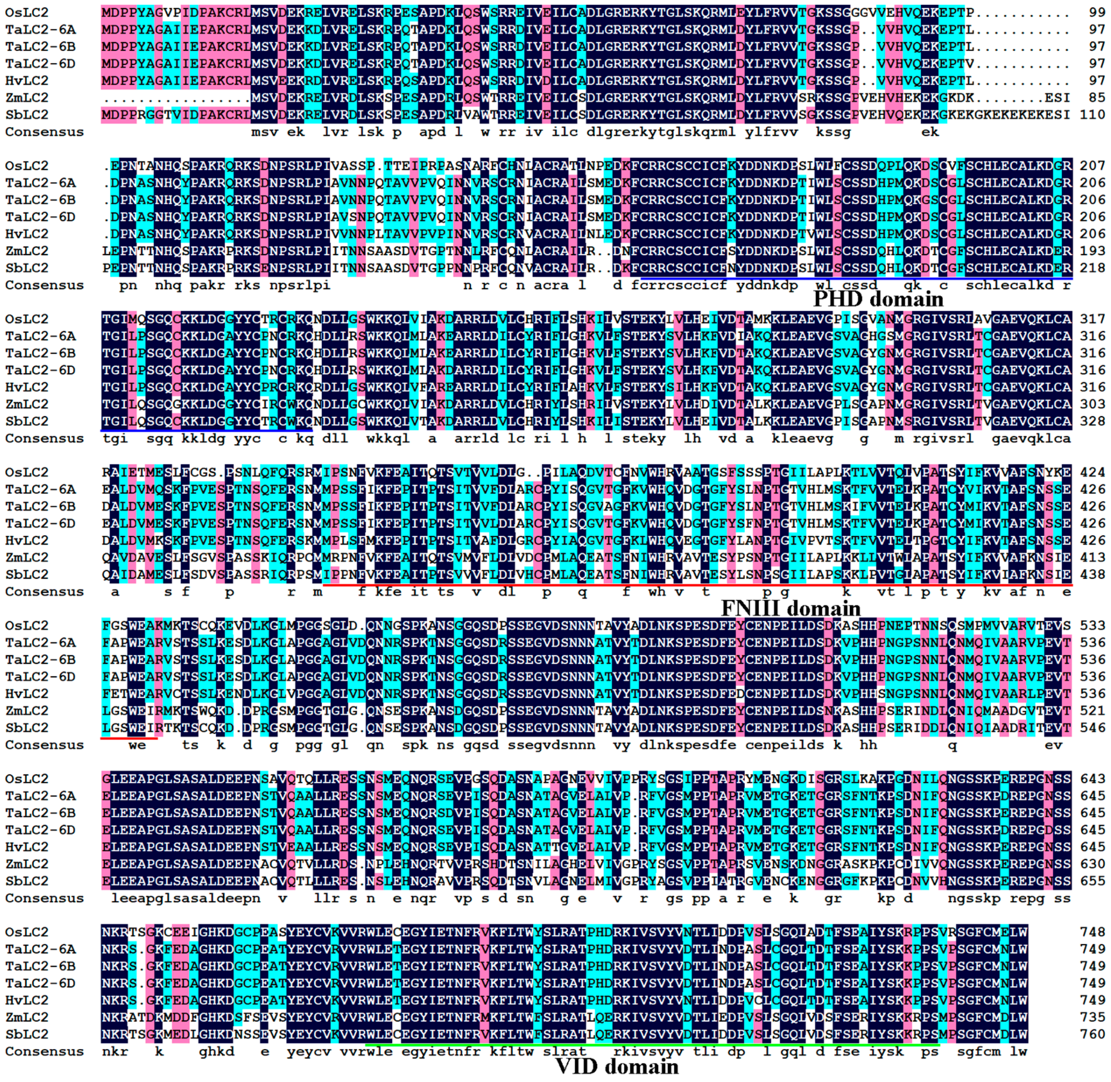

Supplemental Figure S2-24 Alignment of multiple ARF11 and ARF19 proteins in rice, *T. aestivum*, *H. vulgare*, *Z. mays* and *S. bicolor*.

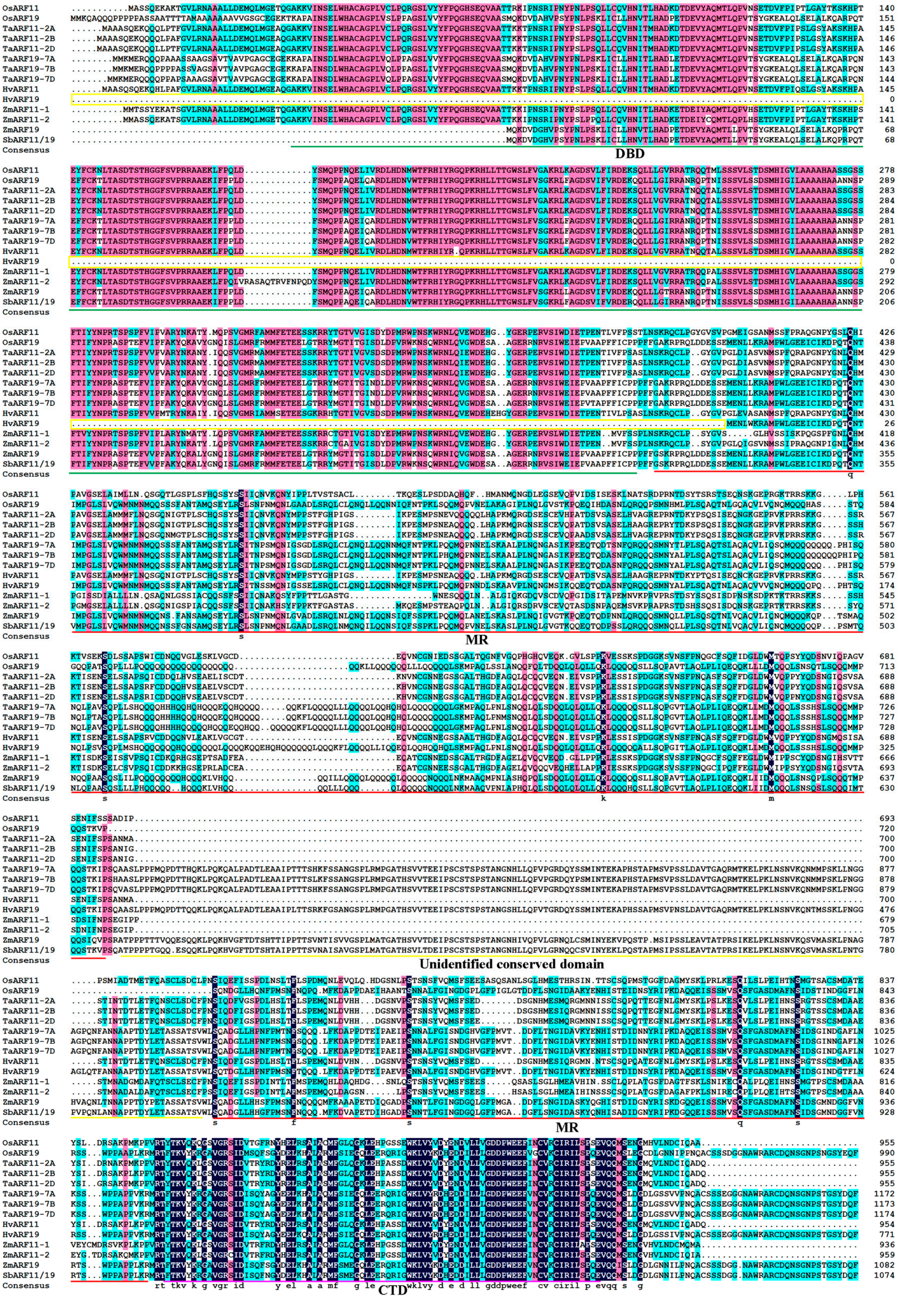

Supplement: Supplementary file 1 [file ijms-23-05551-s001.zip › Figure S2.pdf]
