# Supplementary material for: Genome-Wide Identification of Gramineae Brassinosteroid-Related Genes and Their Roles in Plant Architecture and Salt Stress Adaptation
Source: Int J Mol Sci. 2022 May 16;23(10):5551. doi: 10.3390/ijms23105551 (PMC9146025; doi:10.3390/ijms23105551)

Supplemental Figure SS3 Predicted secondary structures of BR-related plant architecture proteins in *T. aestivum*, *H. vulgare*, *Z. mays* and *S. bicolor*.

Supplemental Figure SS3-1 Predicted secondary structures of DWARF4 and D11 proteins in *T. aestivum*, *H. vulgare*, *Z. mays* and *S. bicolor*.

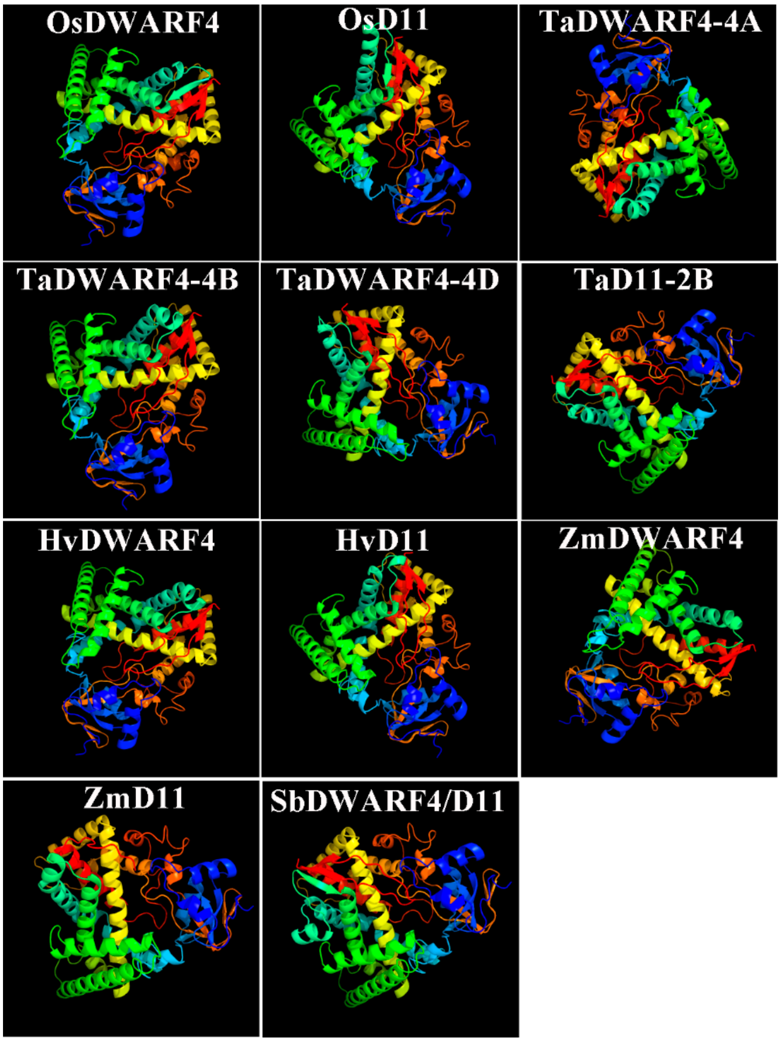

Supplemental Figure SS3-2 Predicted secondary structures of D2 and D3 proteins in *T. aestivum*, *H. vulgare*, *Z. mays* and *S. bicolor*.

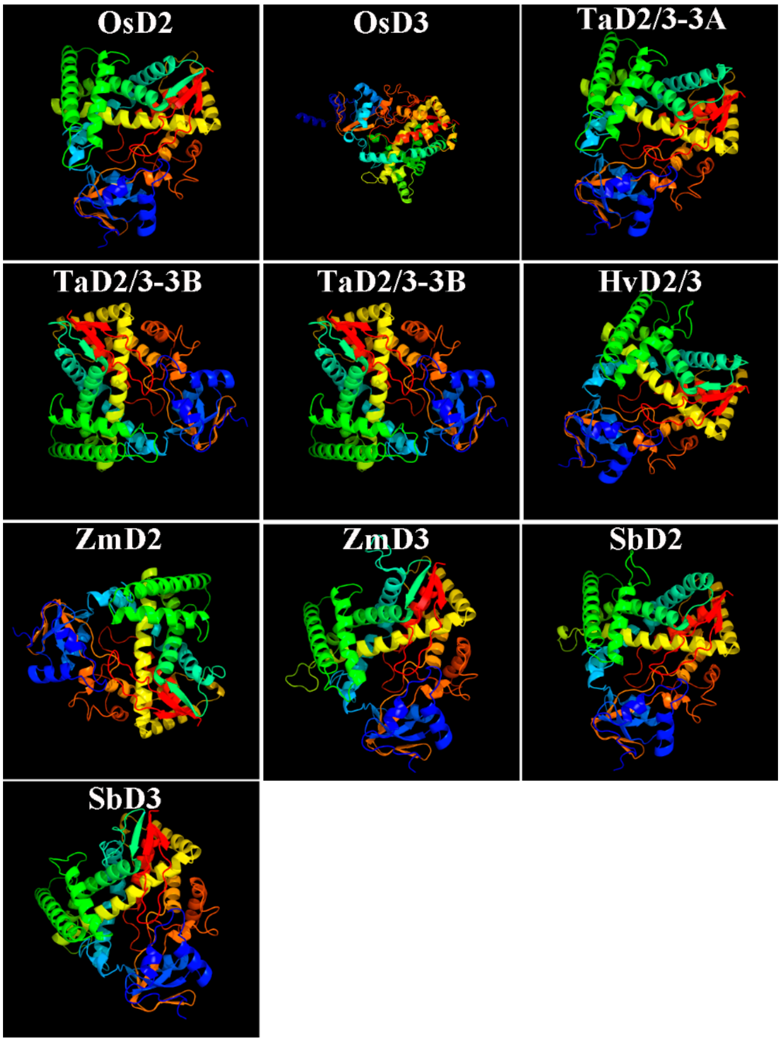

Supplemental Figure SS3-3 Predicted secondary structures of BRD1 proteins in *T. aestivum*, *H. vulgare*, *Z. mays* and *S. bicolor*.

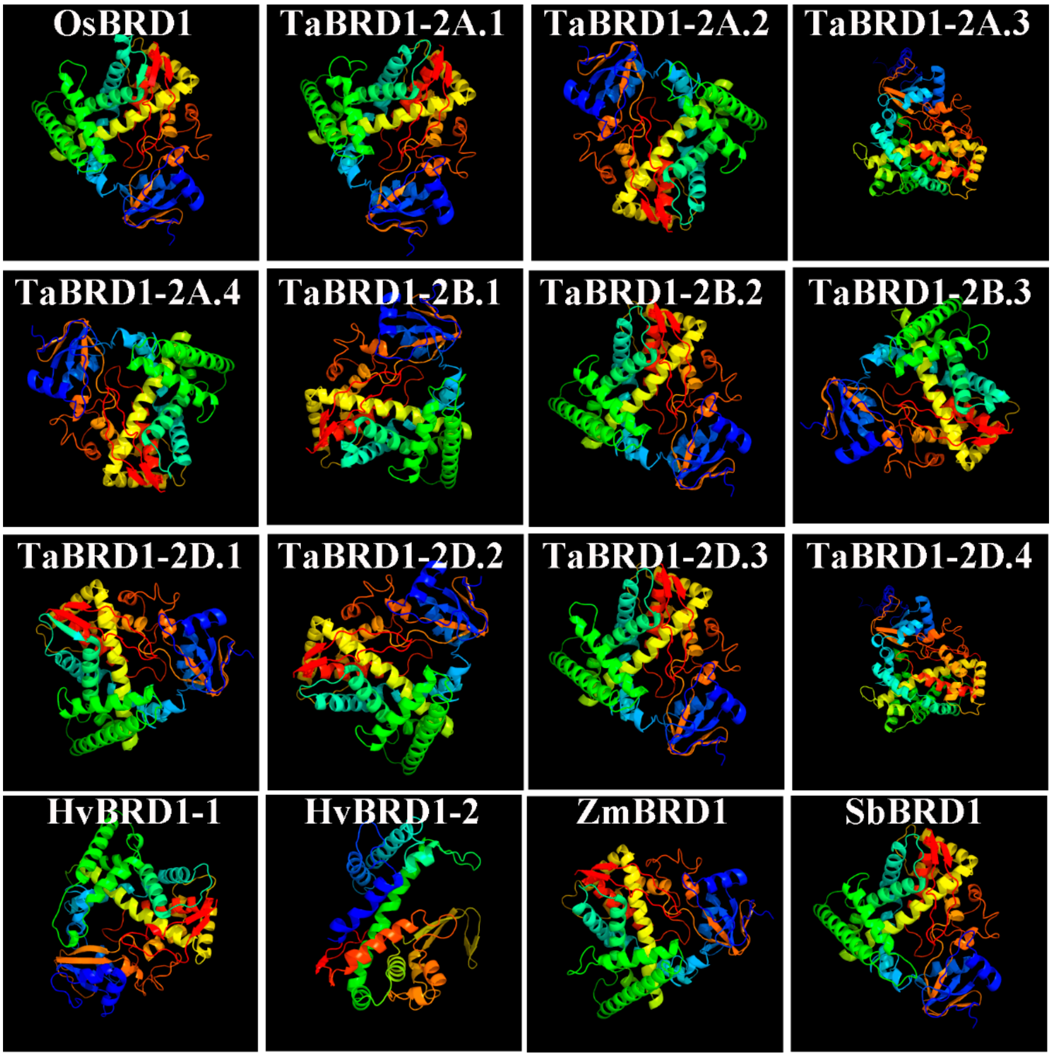

Supplemental Figure S3-4 Predicted secondary structures of BRI1 proteins in *T. aestivum*, *H. vulgare*, *Z. mays* and *S. bicolor*.

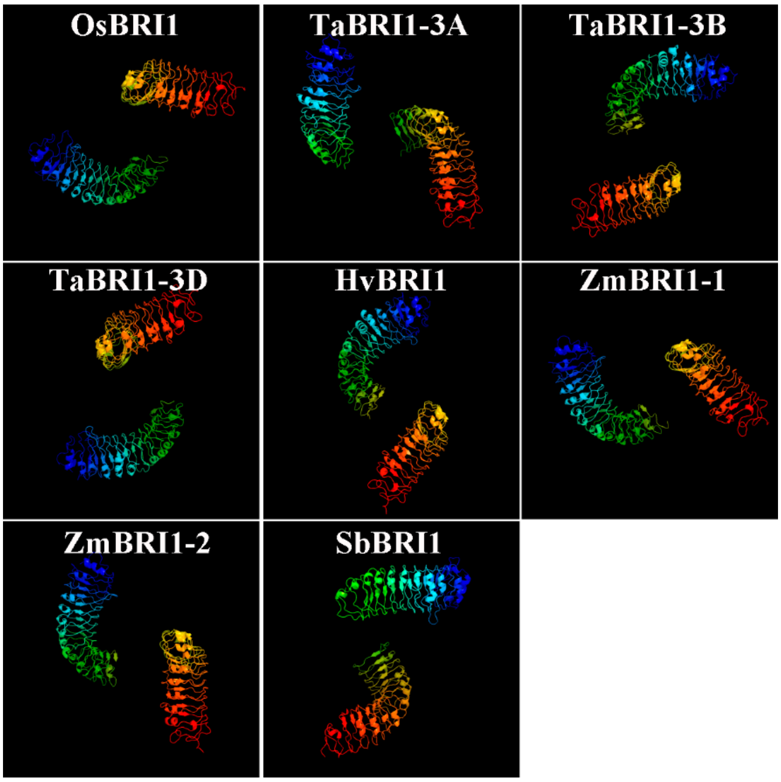

Supplemental Figure S3-5 Predicted secondary structures of BAK1 proteins in *T. aestivum*, *H. vulgare*, *Z. mays* and *S. bicolor*.

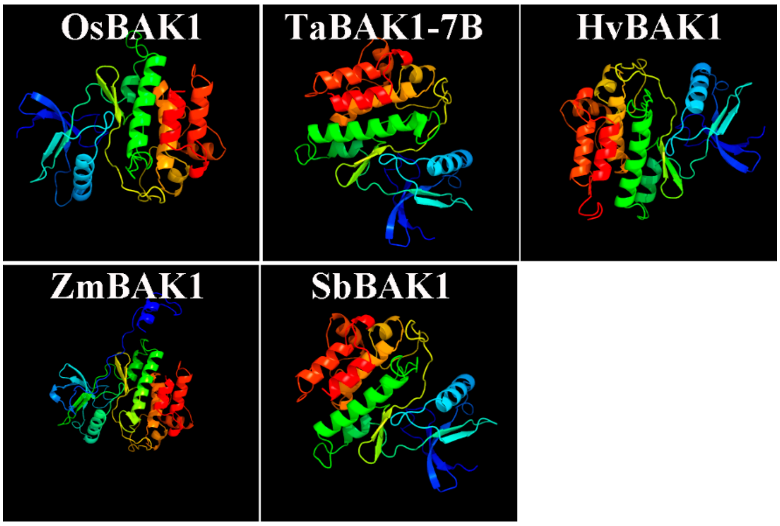

Supplemental Figure S3-6 Predicted secondary structures of GSK1, GSK2, GSK3 and GSK4 proteins in *T. aestivum*, *H. vulgare*, *Z. mays* and *S. bicolor*.

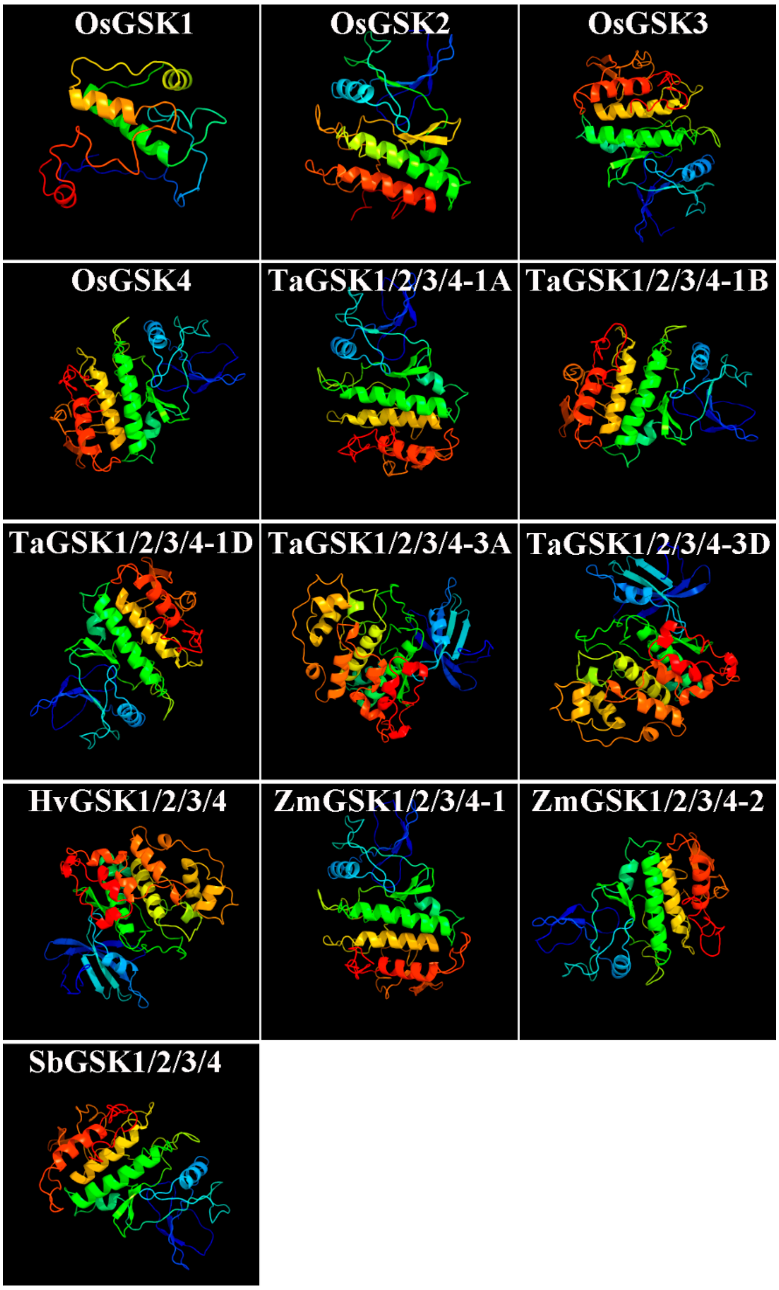

Supplemental Figure S3-7 Predicted secondary structures of BZR1 proteins in *T. aestivum*, *H. vulgare*, *Z. mays* and *S. bicolor*.

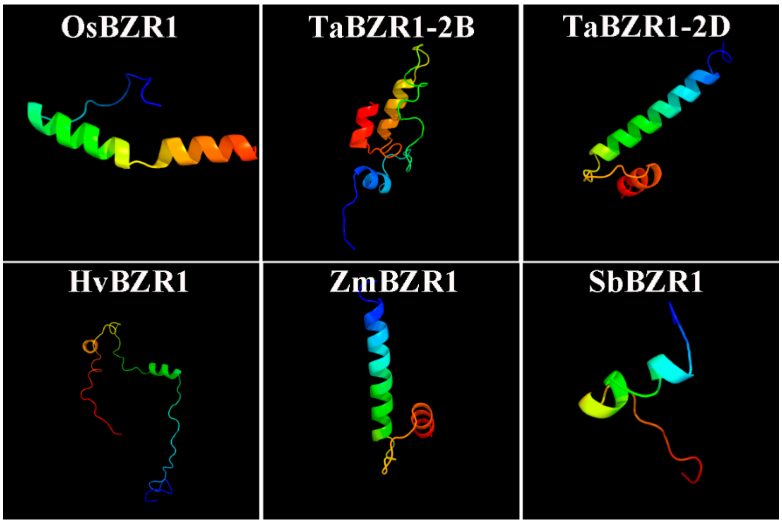

Supplemental Figure S3-8 Predicted secondary structures of SPY proteins in *T. aestivum*, *H. vulgare*, *Z. mays* and *S. bicolor*.

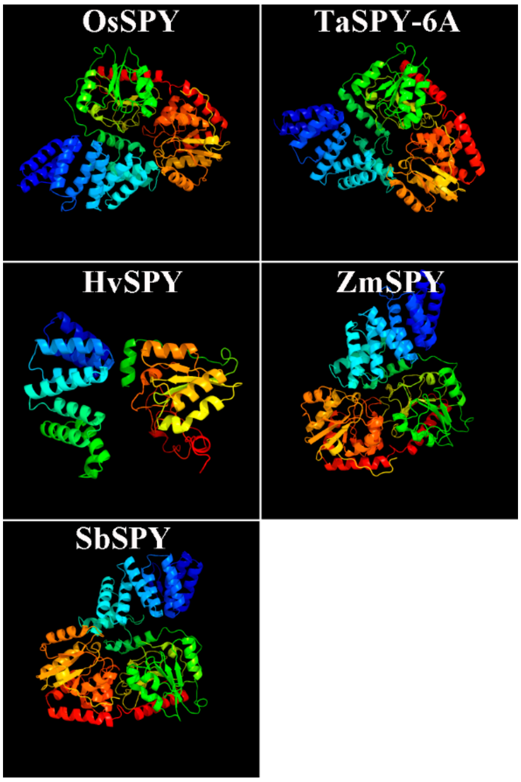

Supplemental Figure S3-9 Predicted secondary structures of GSR1 proteins in *T. aestivum*, *H. vulgare*, *Z. mays* and *S. bicolor*.

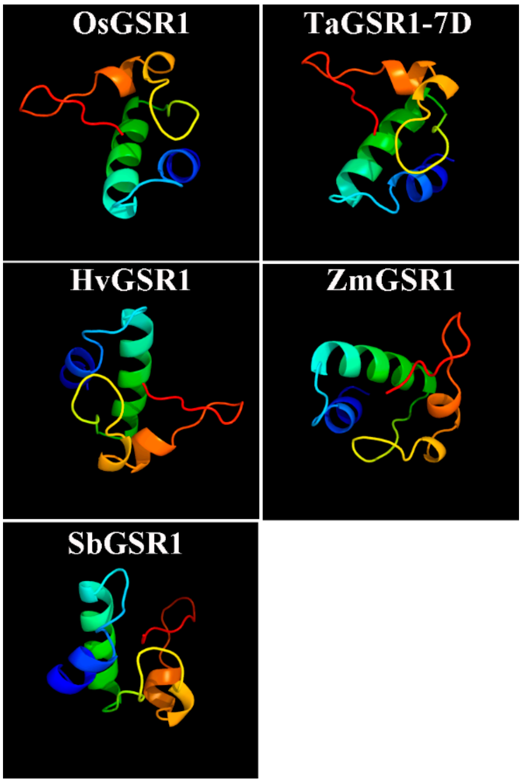

Supplemental Figure S3-10 Predicted secondary structures of ELT1 proteins in *T. aestivum*, *H. vulgare*, *Z. mays* and *S. bicolor*.

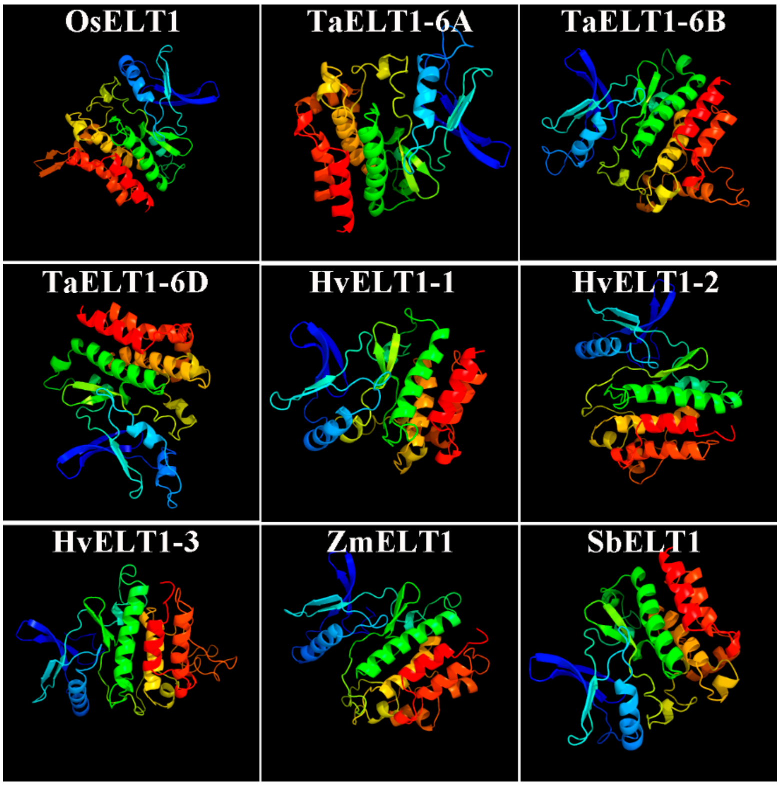

Supplemental Figure S3-11 Predicted secondary structures of SMOS1 proteins in *T. aestivum*, *H. vulgare*, *Z. mays* and *S. bicolor*.

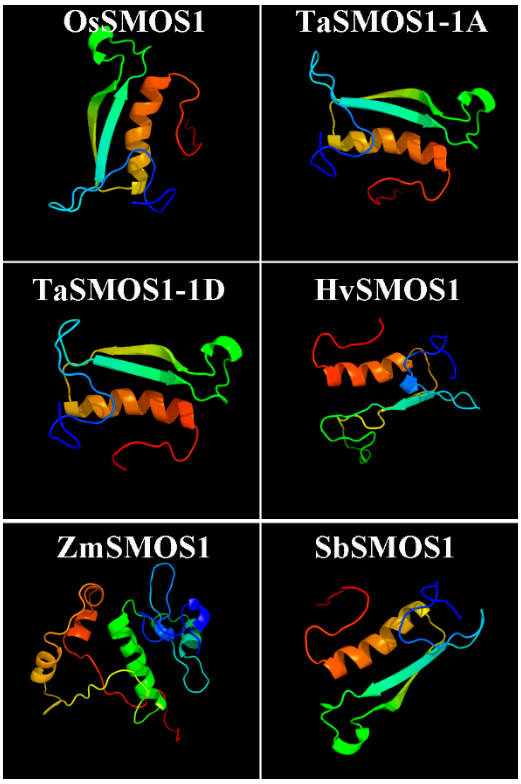

Supplemental Figure S3-12 Predicted secondary structures of DLT proteins in *T. aestivum*, *H. vulgare*, *Z. mays* and *S. bicolor*.

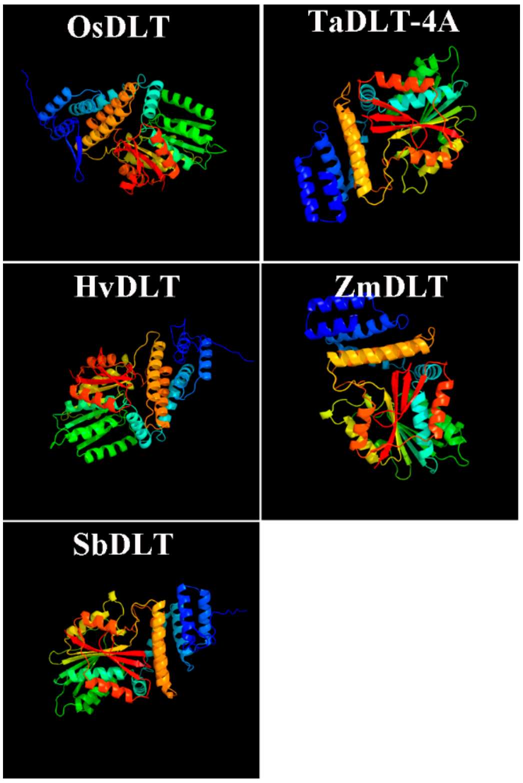

Supplemental Figure S3-13 Predicted secondary structures of OFP1 and OFP8 proteins in *T. aestivum*, *H. vulgare*, *Z. mays* and *S. bicolor*.

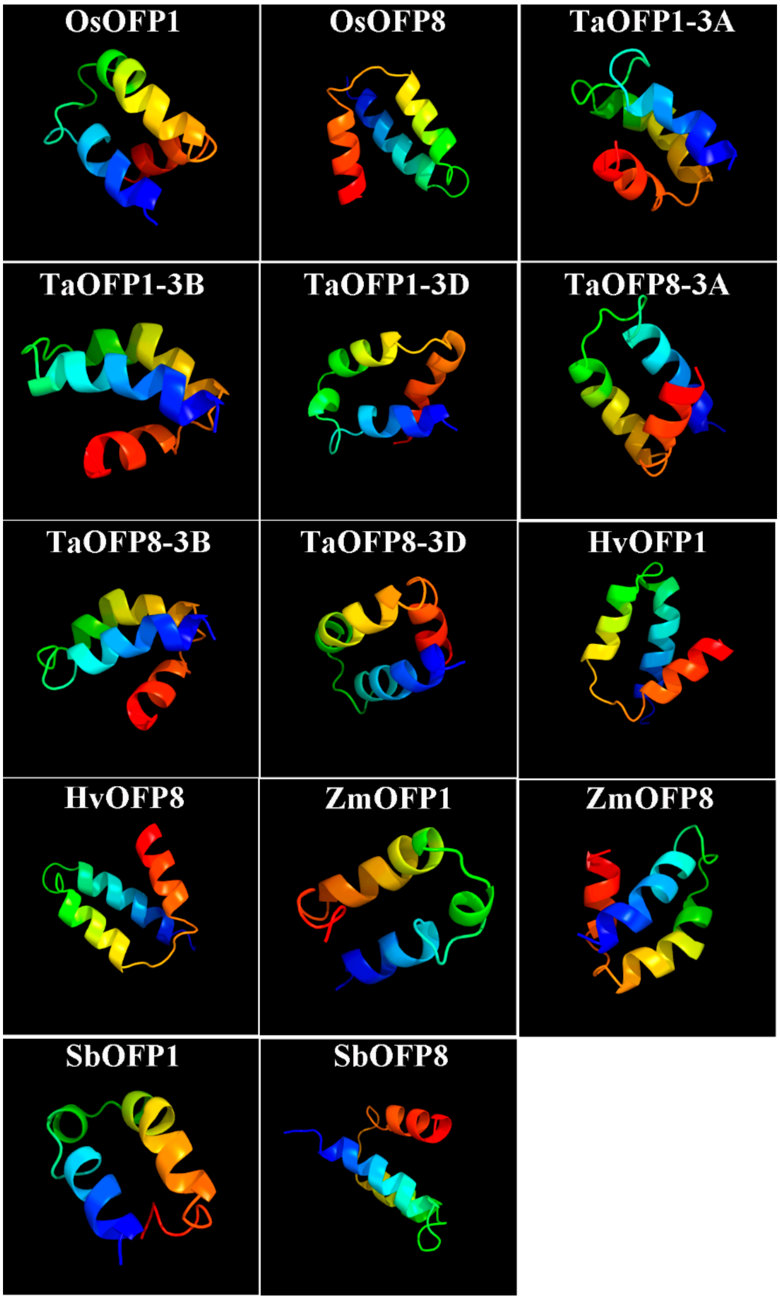

Supplemental Figure S3-14 Predicted secondary structures of LIC proteins in *T. aestivum*, *H. vulgare*, *Z. mays* and *S. bicolor*.

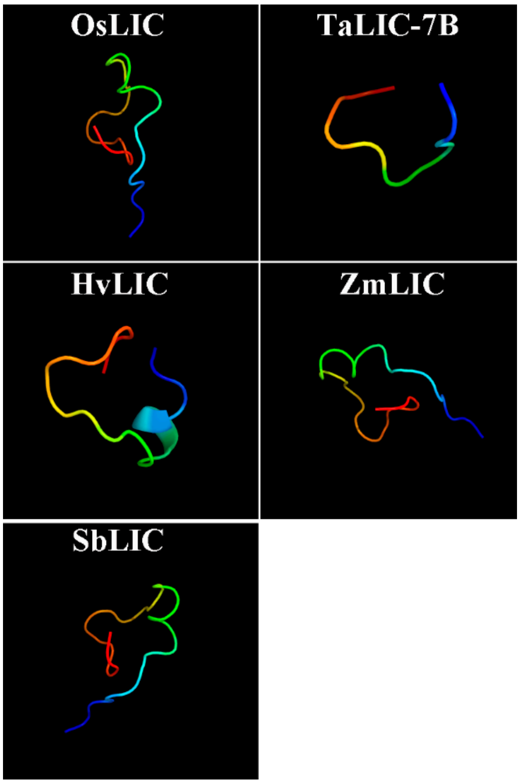

Supplemental Figure S3-15 Predicted secondary structures of CYC U4;1 proteins in *T. aestivum*, *H. vulgare*, *Z. mays* and *S. bicolor*.

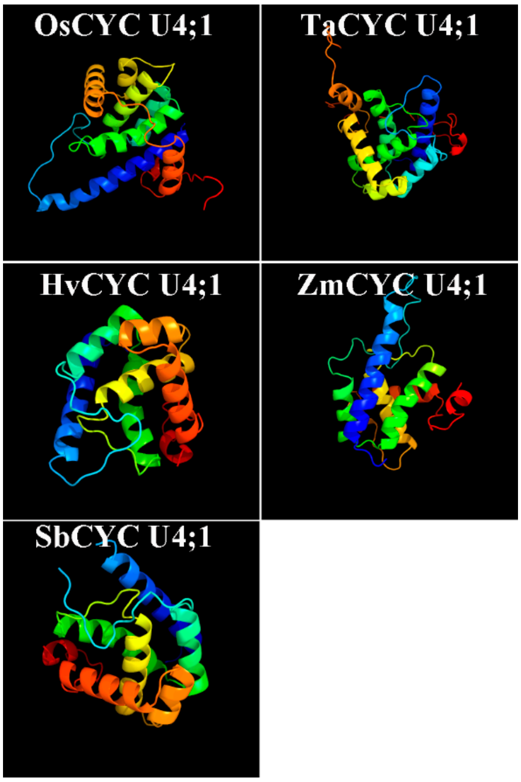

Supplemental Figure S3-16 Predicted secondary structures of TUD1 proteins in *T. aestivum*, *H. vulgare*, *Z. mays* and *S. bicolor*.

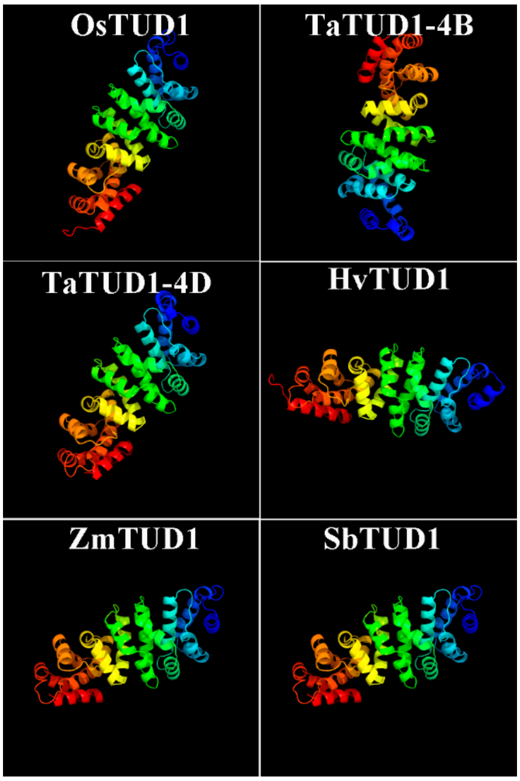

Supplemental Figure S3-17 Predicted secondary structures of D1 proteins in *T. aestivum*, *H. vulgare*, *Z. mays* and *S. bicolor*.

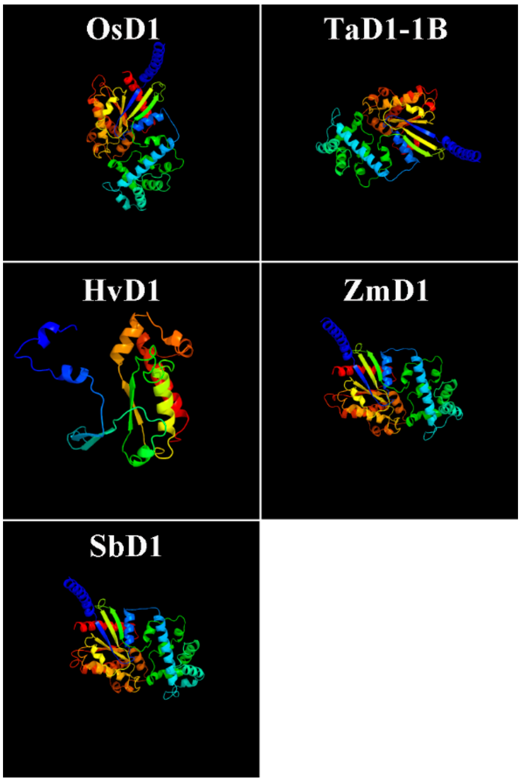

Supplemental Figure S3-18 Predicted secondary structures of BU1, BUL1 and ILI1 proteins in *T. aestivum*, *H. vulgare*, *Z. mays* and *S. bicolor*.

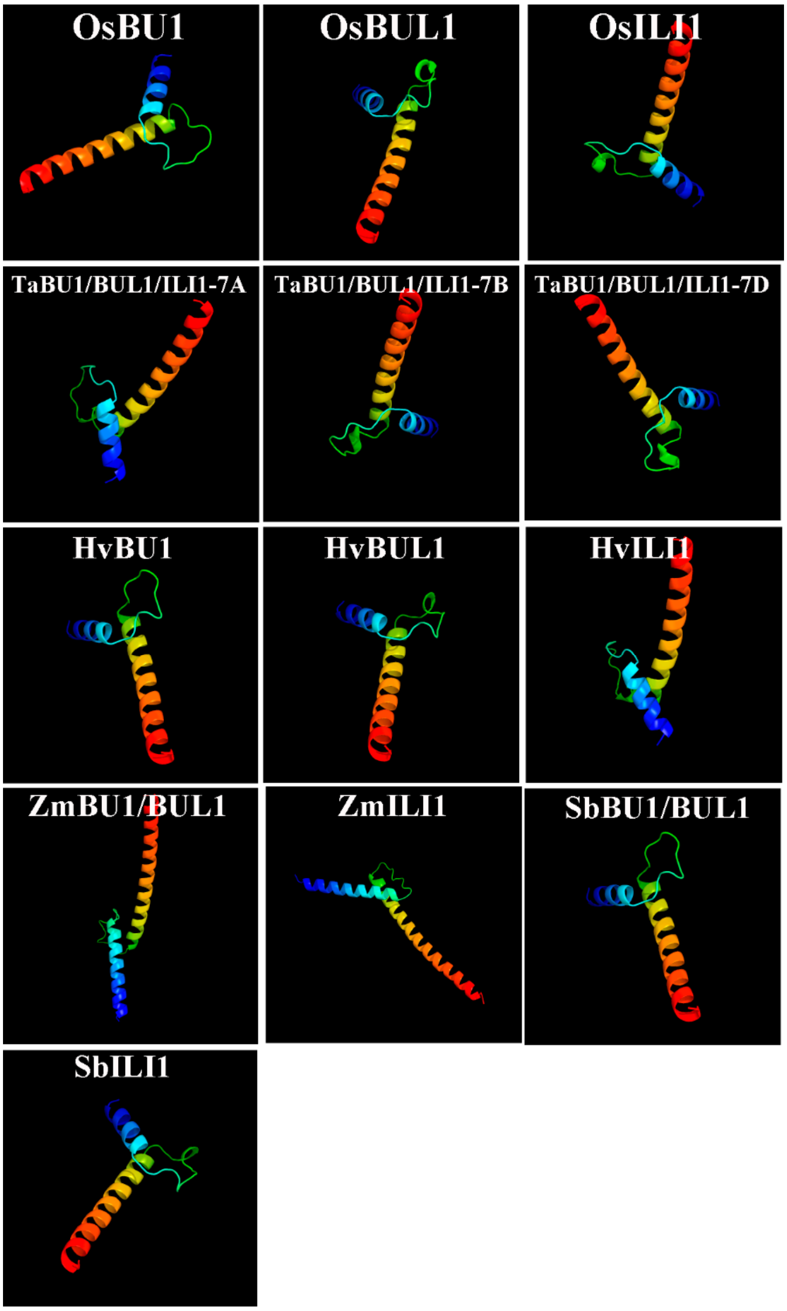

Supplemental Figure S3-19 Predicted secondary structures of IBH1 proteins in *T. aestivum*, *H. vulgare*, *Z. mays* and *S. bicolor*.

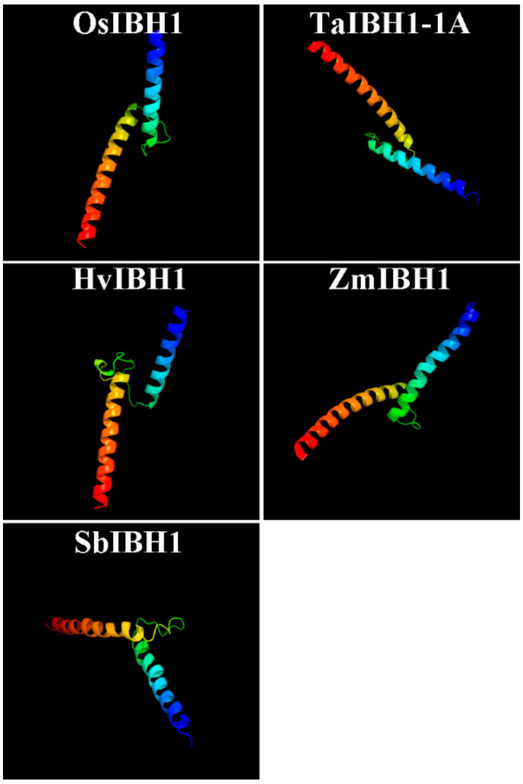

Supplemental Figure S3-20 Predicted secondary structures of GRAS19 proteins in *T. aestivum*, *H. vulgare*, *Z. mays* and *S. bicolor*.

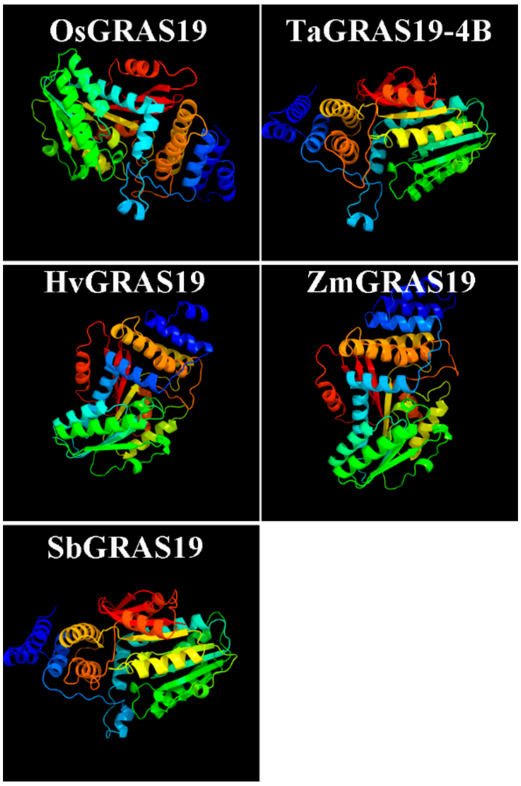

Supplemental Figure S3-21 Predicted secondary structures of MADS22, MADS47 and MADS55 proteins in *T. aestivum*, *H. vulgare*, *Z. mays* and *S. bicolor*.

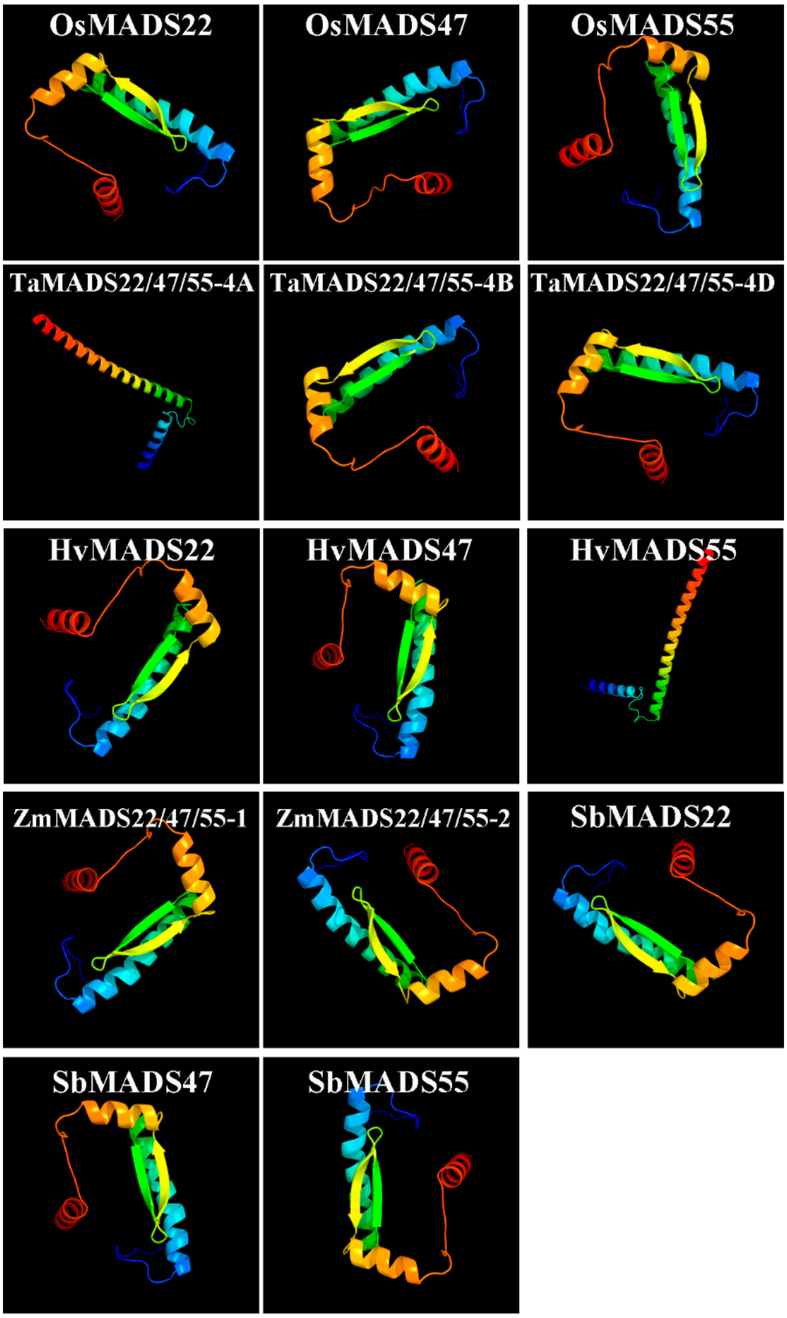

Supplemental Figure S3-22 Predicted secondary structures of XIAO proteins in *T. aestivum*, *H. vulgare*, *Z. mays* and *S. bicolor*.

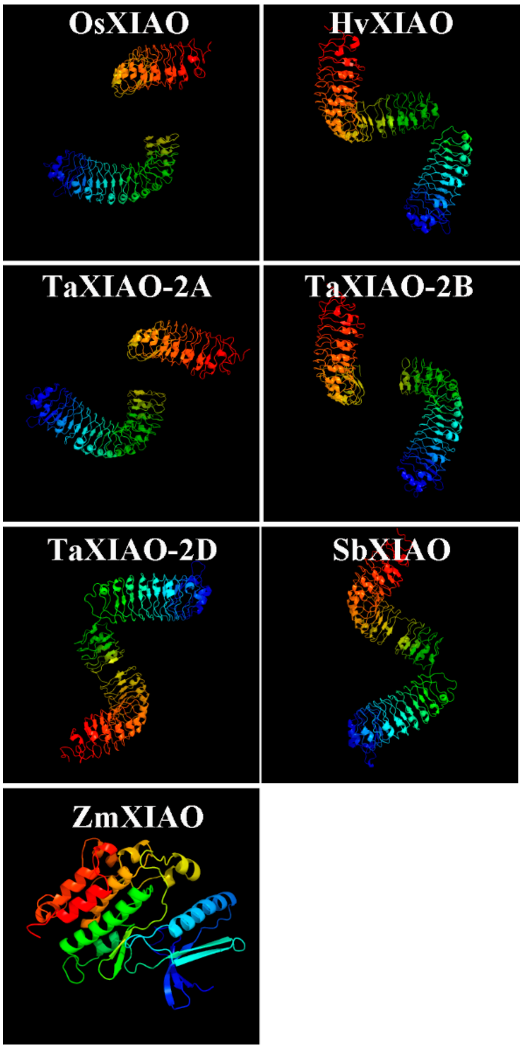

Supplemental Figure S3-23 Predicted secondary structures of LC2 proteins in *T. aestivum*, *H. vulgare*, *Z. mays* and *S. bicolor*.

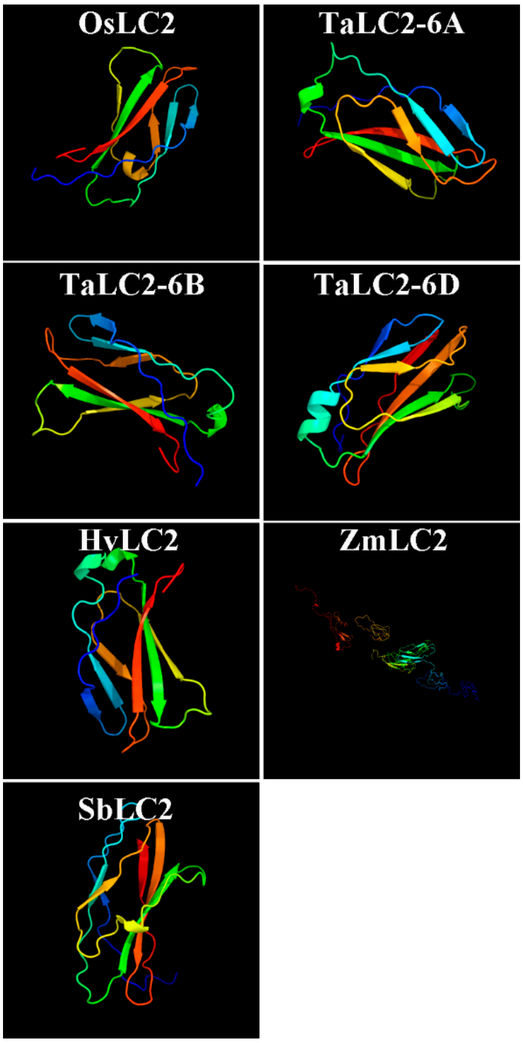

Supplemental Figure S3-24 Predicted secondary structures of ARF11 and ARF19 proteins in *T. aestivum*, *H. vulgare*, *Z. mays* and *S. bicolor*.

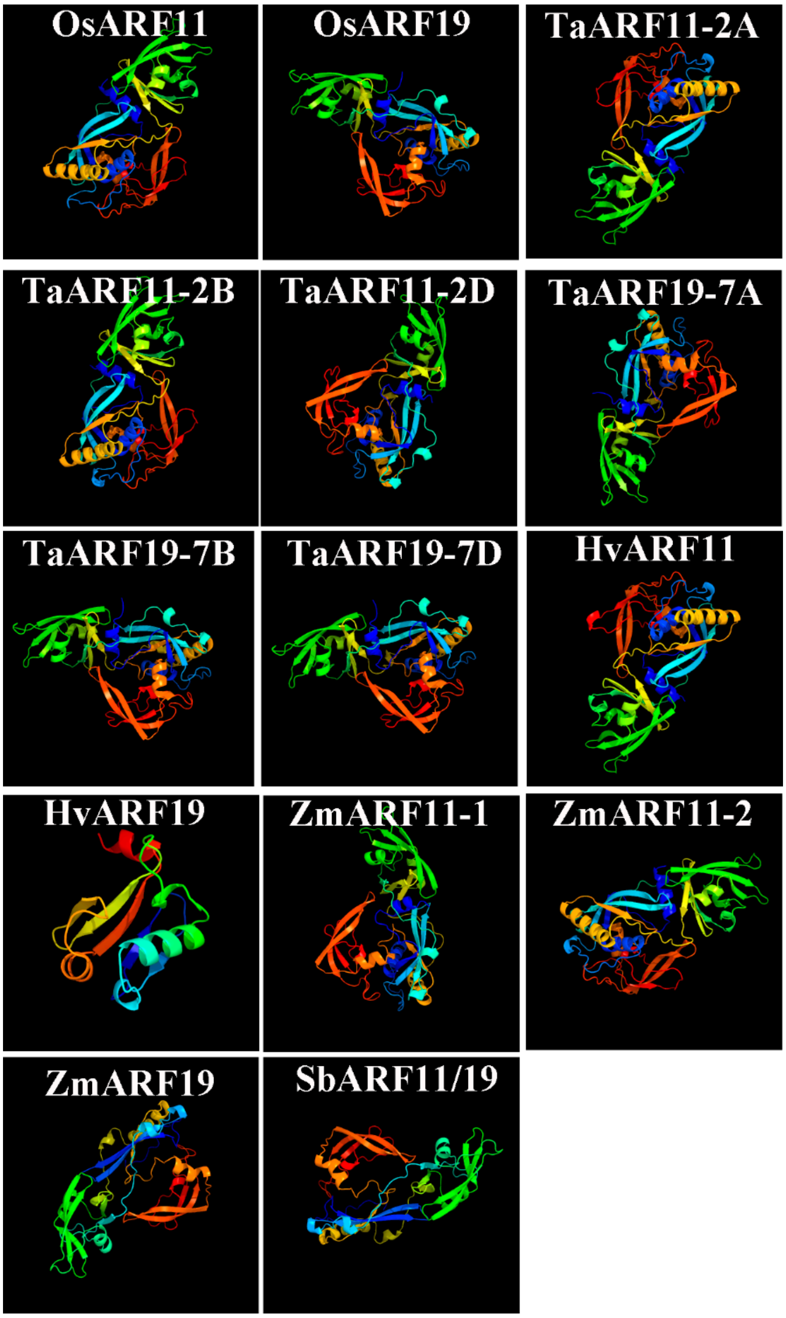

Supplement: Supplementary file 1 [file ijms-23-05551-s001.zip › Figure S3.pdf]
