# Supplementary material for: Genome-Wide Identification of Gramineae Brassinosteroid-Related Genes and Their Roles in Plant Architecture and Salt Stress Adaptation
Source: Int J Mol Sci. 2022 May 16;23(10):5551. doi: 10.3390/ijms23105551 (PMC9146025; doi:10.3390/ijms23105551)

Supplemental Figure S4 Transmembrane topology analysis of BR-related plant architecture proteins in *T. aestivum*, *H. vulgare*, *Z. mays* and *S. bicolor*.

Supplemental Figure S4-1 Transmembrane topology analysis of DWARF4 and D11 proteins in *T. aestivum*, *H. vulgare*, *Z. mays* and *S. bicolor*.

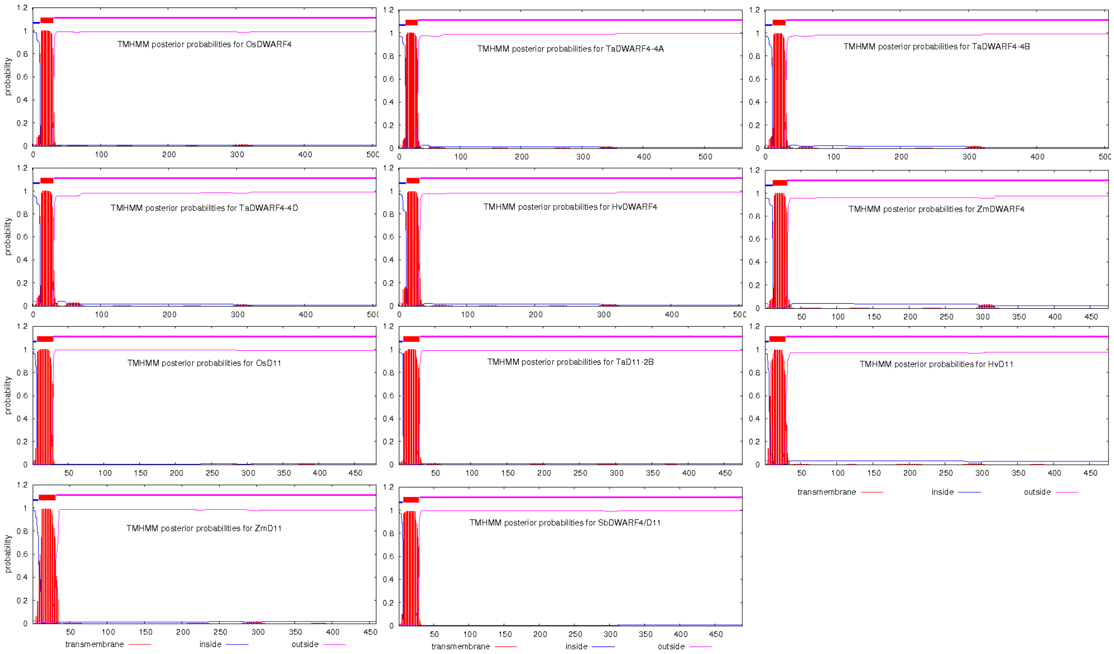

Supplemental Figure S4-2 Transmembrane topology analysis of D2 and D3 proteins in *T. aestivum*, *H. vulgare*, *Z. mays* and *S. bicolor*.

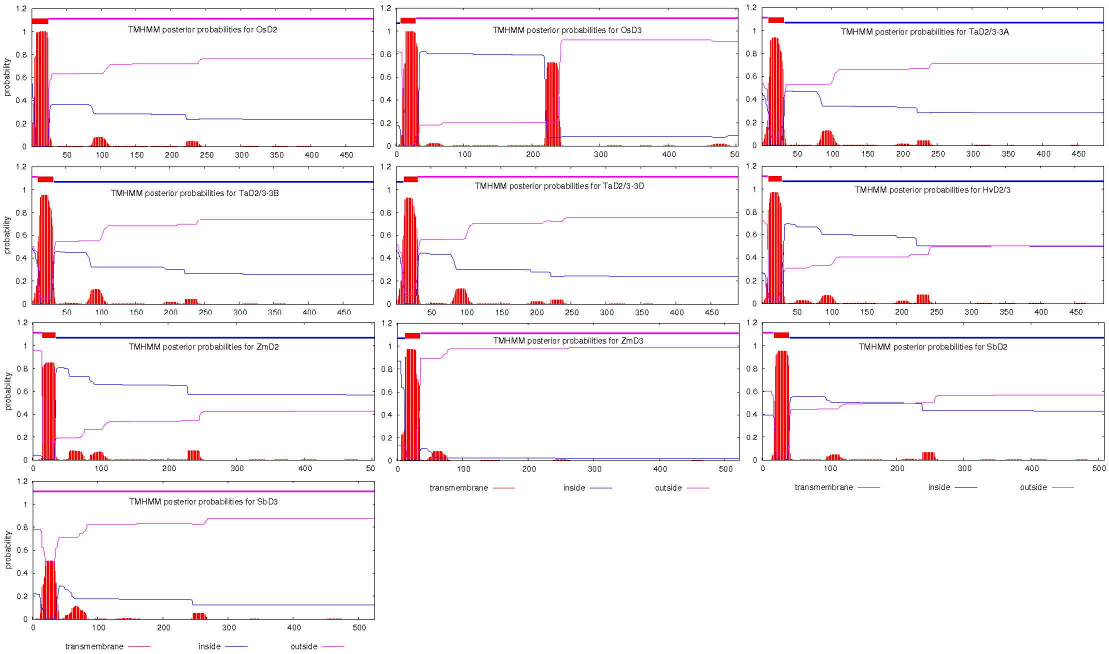

Supplemental Figure S4-3 Transmembrane topology analysis of BRD1 proteins in *T. aestivum*, *H. vulgare*, *Z. mays* and *S. bicolor*.

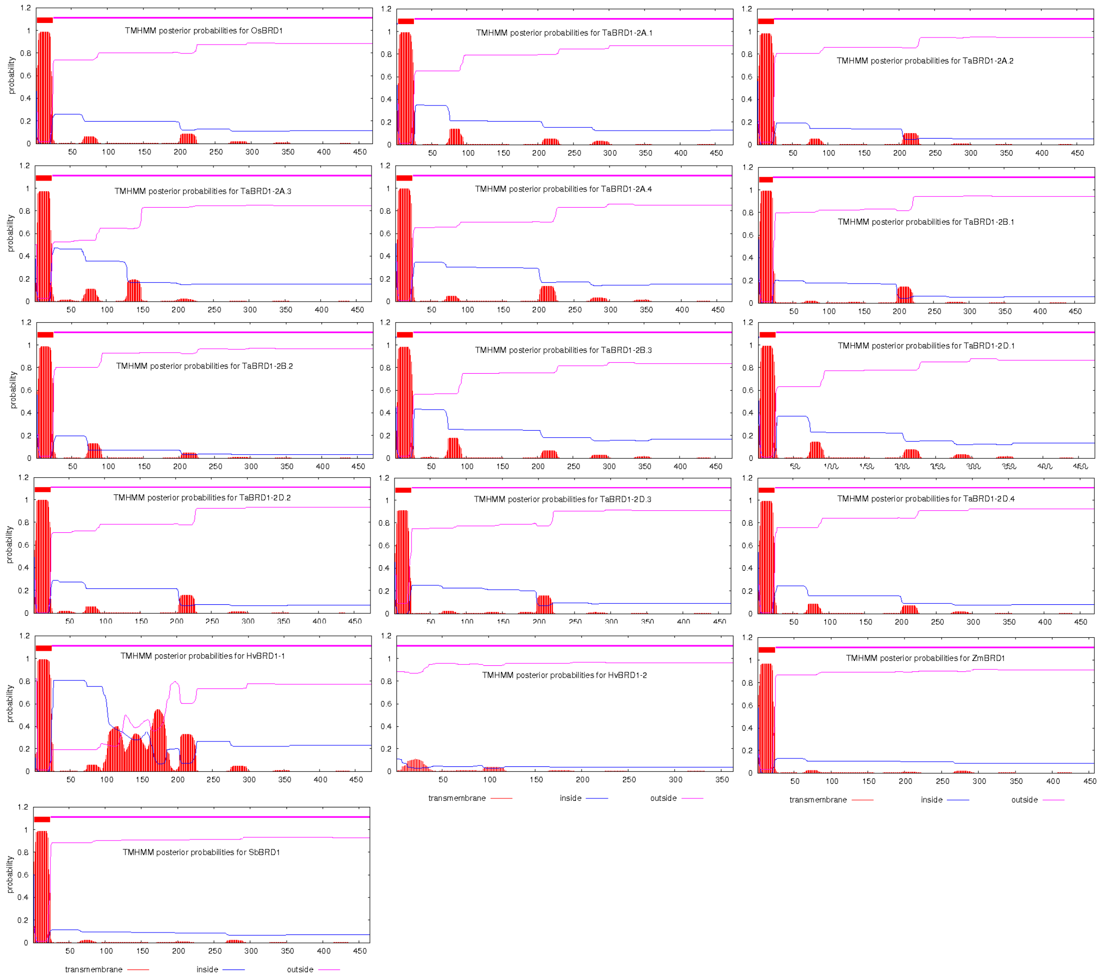

Supplemental Figure S4-4 Transmembrane topology analysis of BRI1 proteins in *T. aestivum*, *H. vulgare*, *Z. mays* and *S. bicolor*.

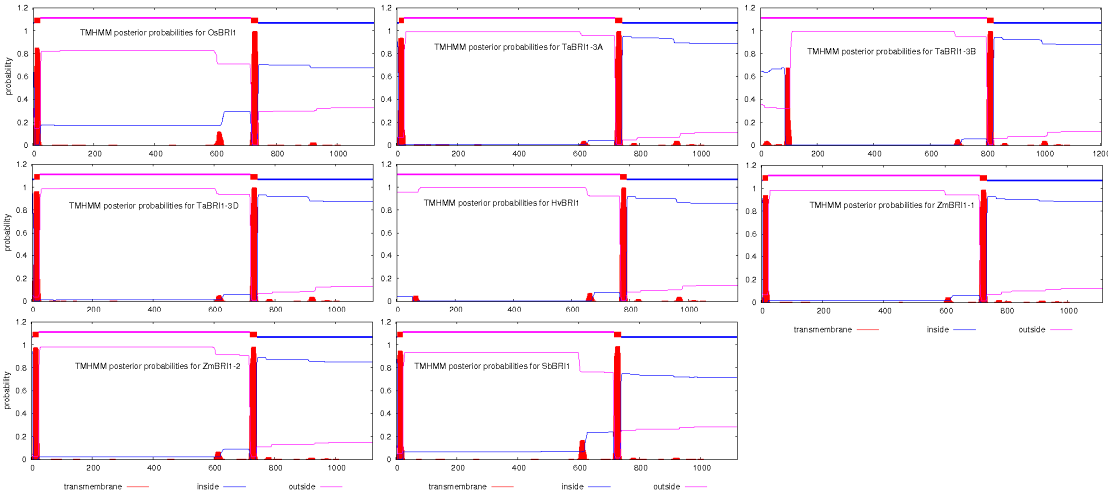

Supplemental Figure S4-5 Transmembrane topology analysis of AK1 proteins in *T. aestivum*, *H. vulgare*, *Z. mays* and *S. bicolor*.

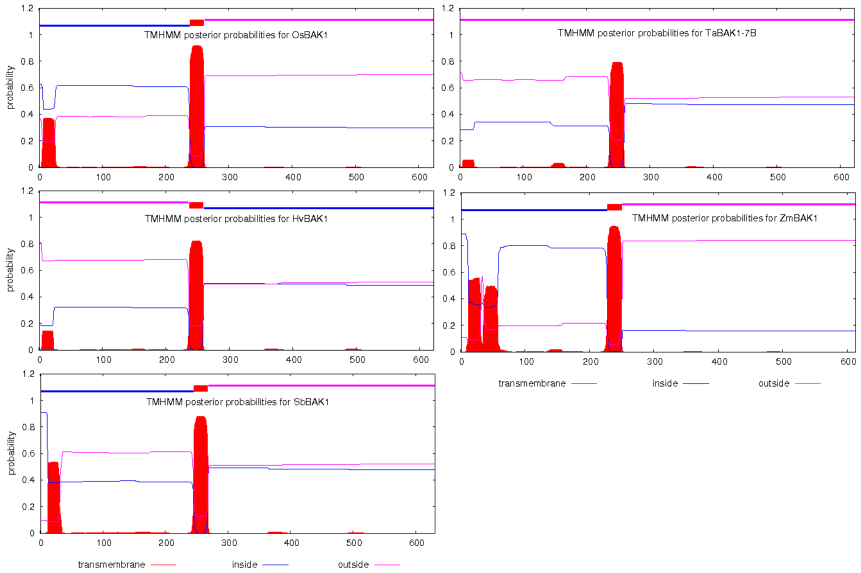

Supplemental Figure S4-6 Transmembrane topology analysis of GSK1, GSK2, GSK3 and GSK4 proteins in *T. aestivum*, *H. vulgare*, *Z. mays* and *S. bicolor*.

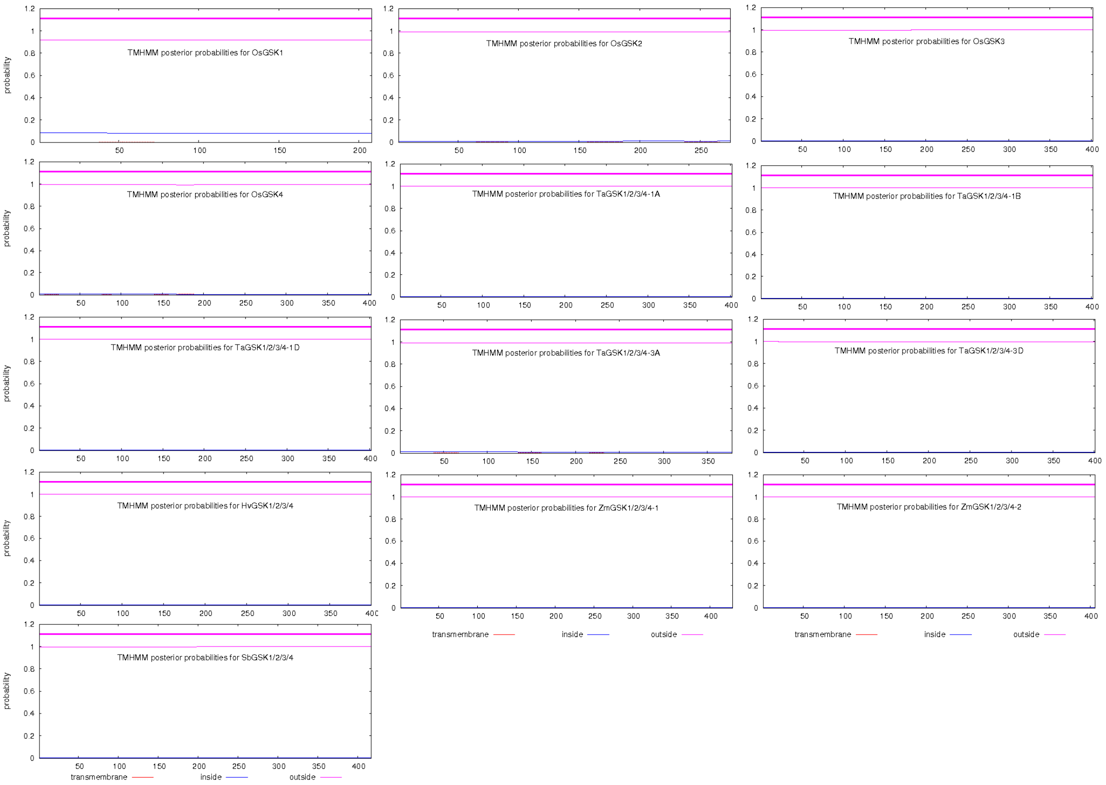

Supplemental Figure S4-7 Transmembrane topology analysis of BZR1 proteins in *T. aestivum*, *H. vulgare*, *Z. mays* and *S. bicolor*.

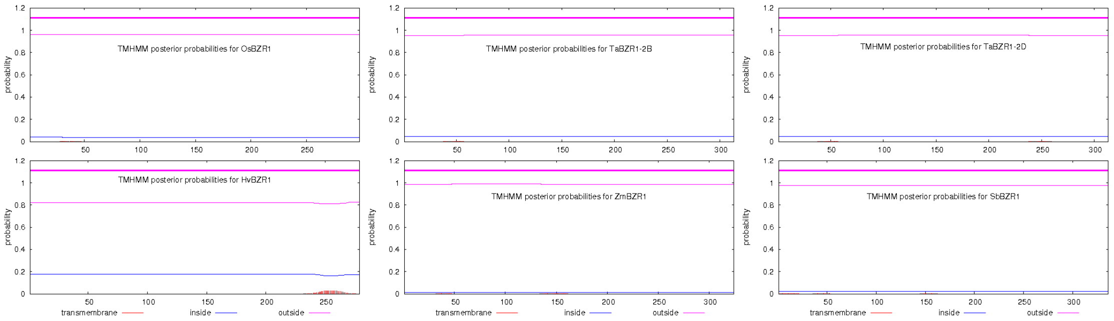

Supplemental Figure S4-8 Transmembrane topology analysis of SPY proteins in *T. aestivum*, *H. vulgare*, *Z. mays* and *S. bicolor*.

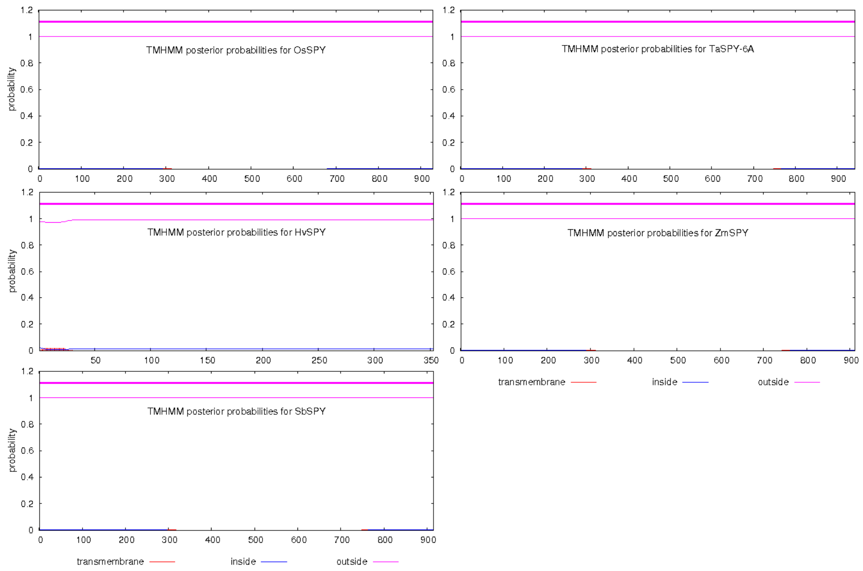

Supplemental Figure S4-9 Transmembrane topology analysis of GSR1 proteins in *T. aestivum*, *H. vulgare*, *Z. mays* and *S. bicolor*.

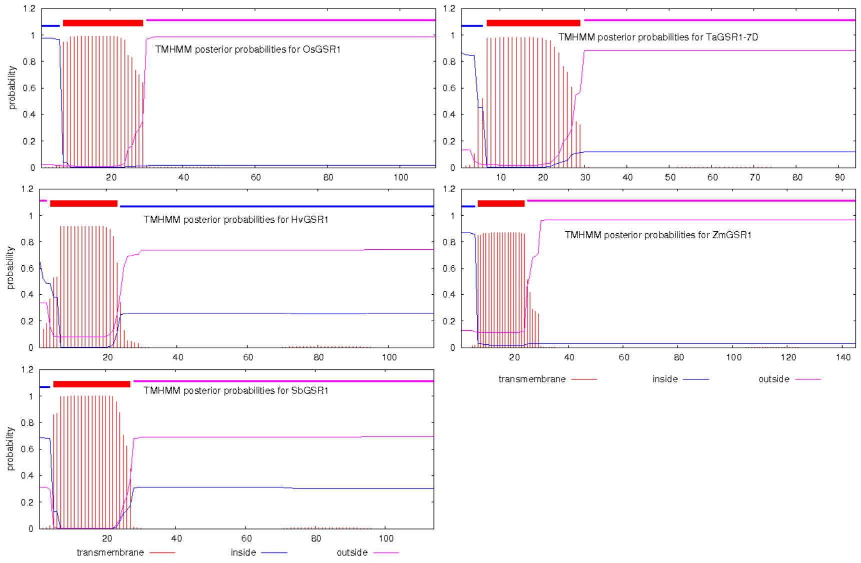

Supplemental Figure S4-10 Transmembrane topology analysis of ELT1 proteins in *T. aestivum*, *H. vulgare*, *Z. mays* and *S. bicolor*.

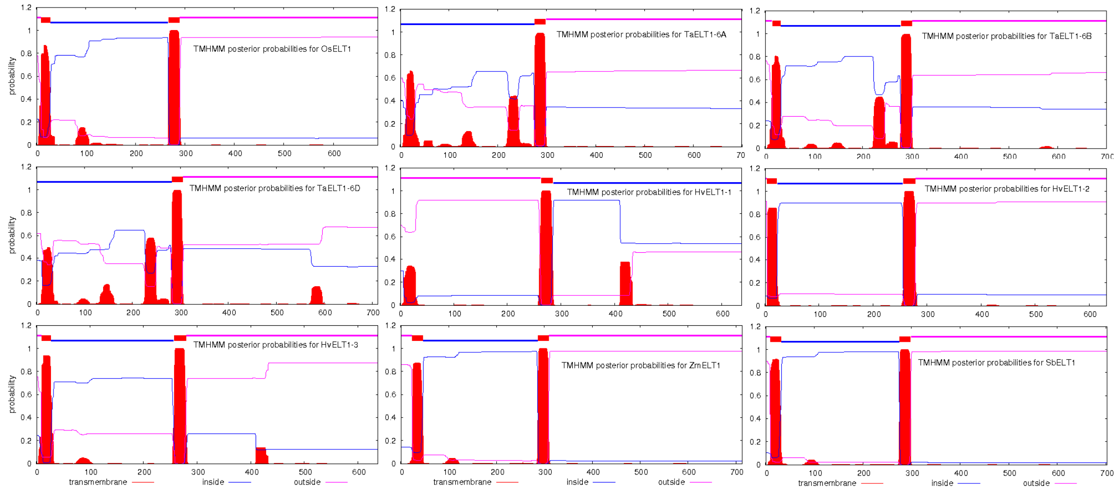

Supplemental Figure S4-11 Transmembrane topology analysis of SMOS1 proteins in *T. aestivum*, *H. vulgare*, *Z. mays* and *S. bicolor*.

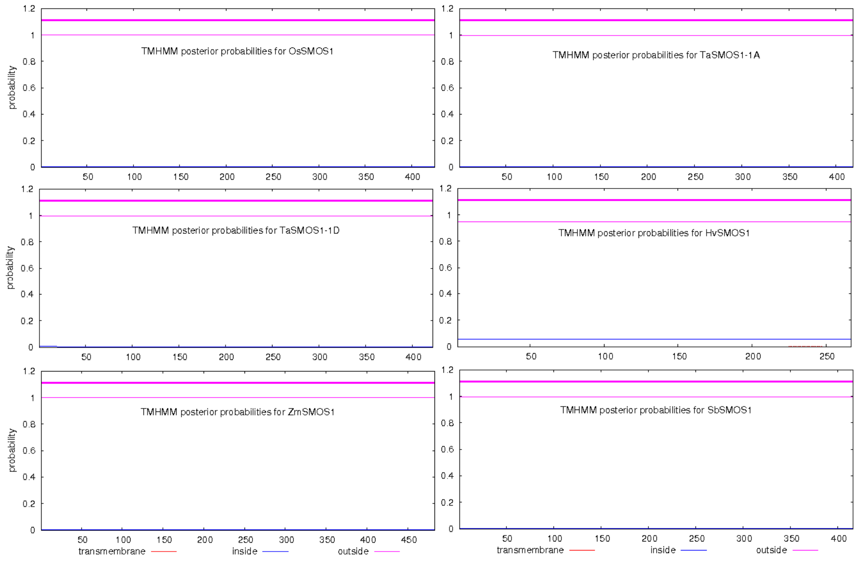

Supplemental Figure S4-12 Transmembrane topology analysis of DLT proteins in *T. aestivum*, *H. vulgare*, *Z. mays* and *S. bicolor*.

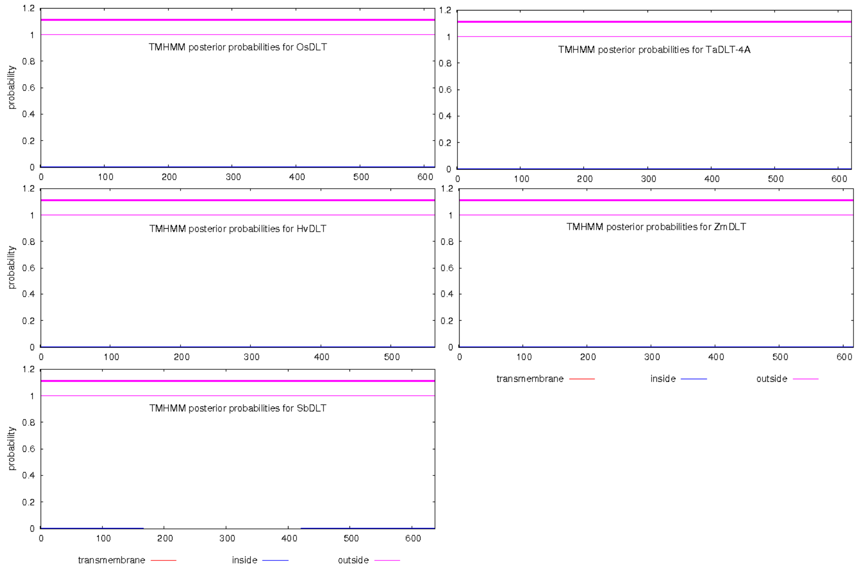

Supplemental Figure S4-13 Transmembrane topology analysis of OFP1 and OFP8 proteins in *T. aestivum*, *H. vulgare*, *Z. mays* and *S. bicolor*.

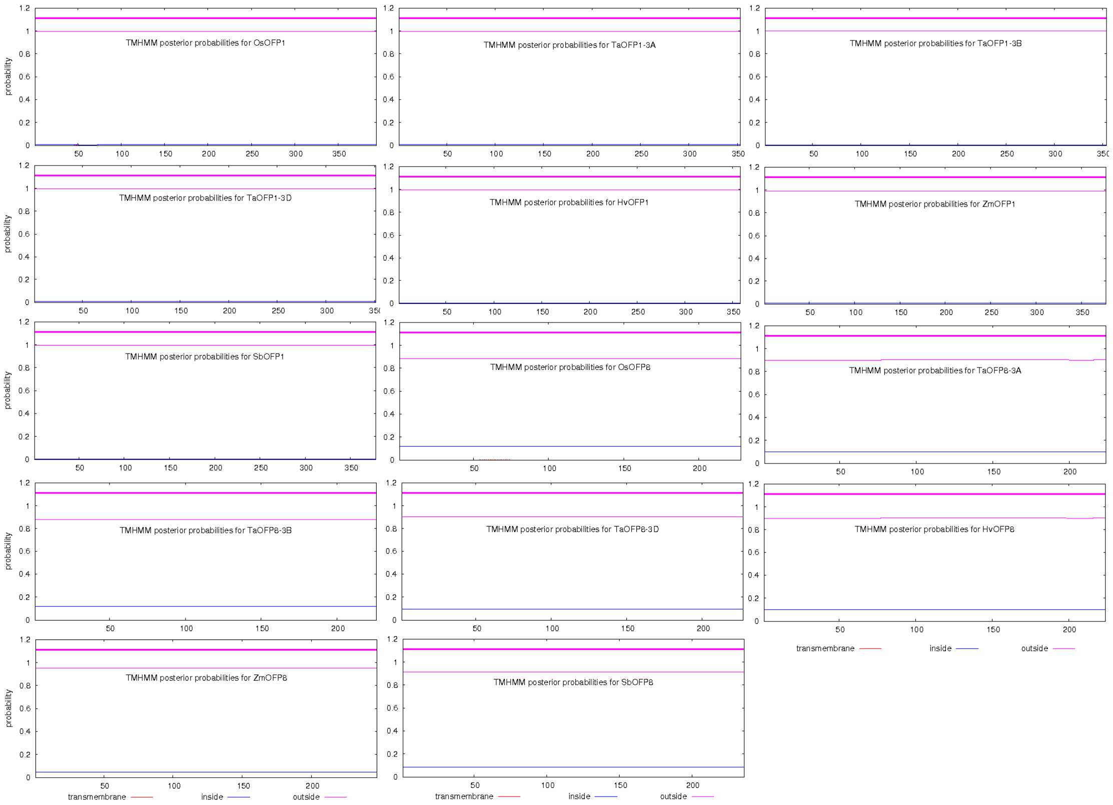

Supplemental Figure S4-14 Transmembrane topology analysis of LIC proteins in *T. aestivum*, *H. vulgare*, *Z. mays* and *S. bicolor*.

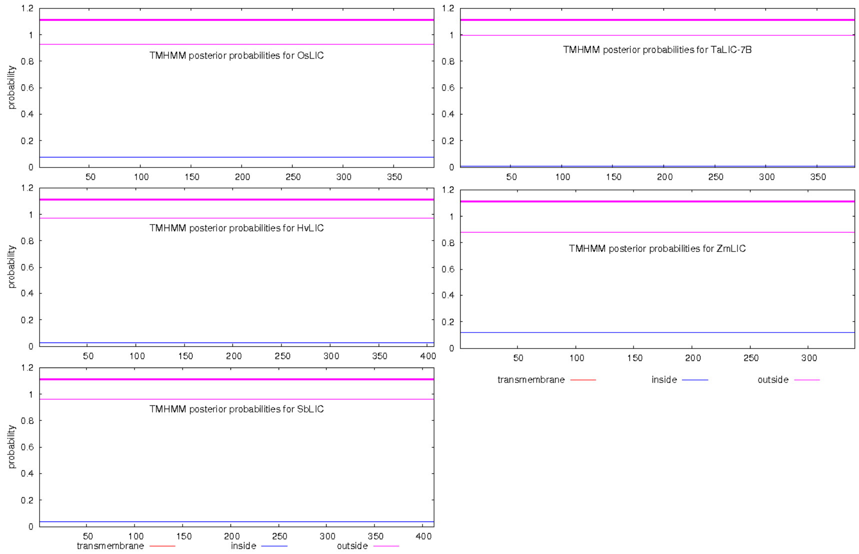

Supplemental Figure S4-15 Transmembrane topology analysis of CYC U4;1 proteins in *T. aestivum*, *H. vulgare*, *Z. mays* and *S. bicolor*.

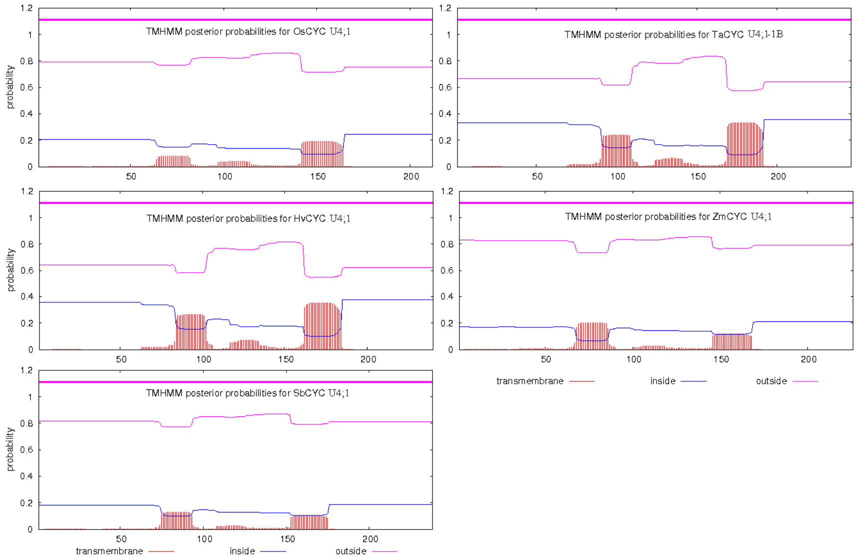

Supplemental Figure S4-16 Transmembrane topology analysis of TUD1 proteins in *T. aestivum*, *H. vulgare*, *Z. mays* and *S. bicolor*.

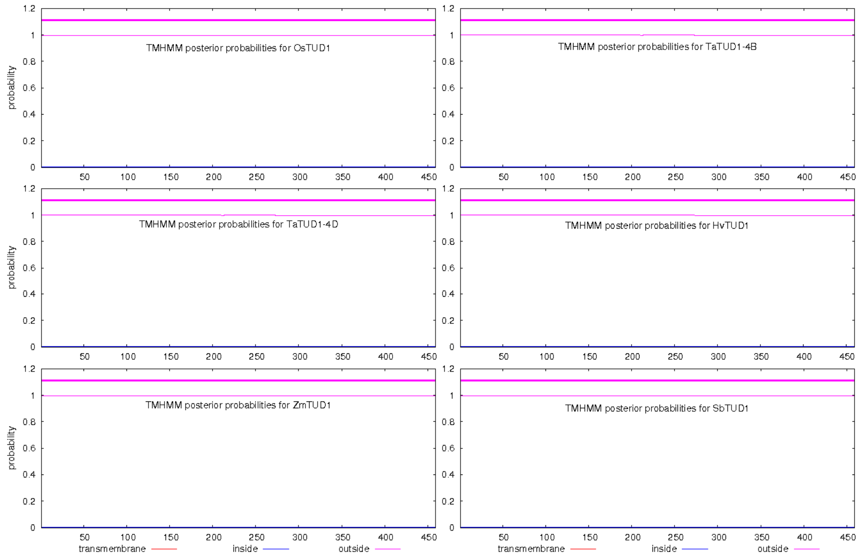

Supplemental Figure S4-17 Transmembrane topology analysis of D1 proteins in *T. aestivum*, *H. vulgare*, *Z. mays* and *S. bicolor*.

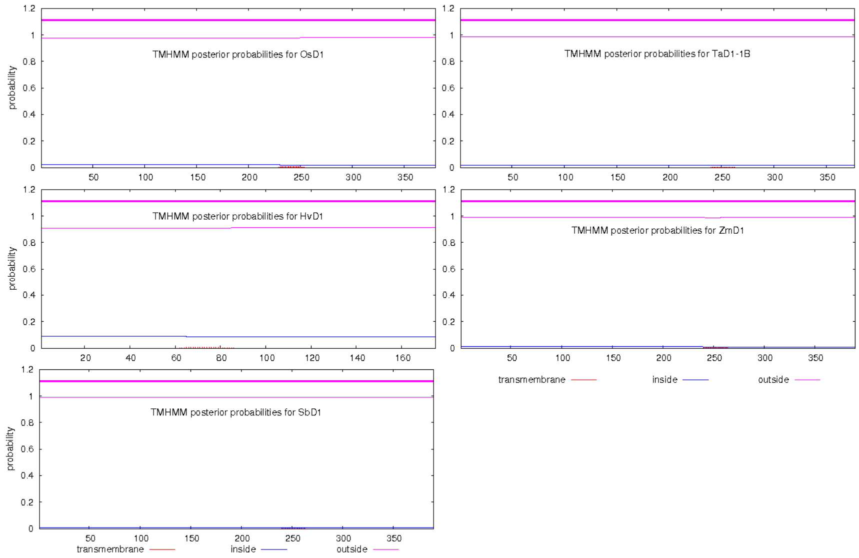

Supplemental Figure S4-18 Transmembrane topology analysis of BU1, ILI1 and BUL1 proteins in *T. aestivum*, *H. vulgare*, *Z. mays* and *S. bicolor*.

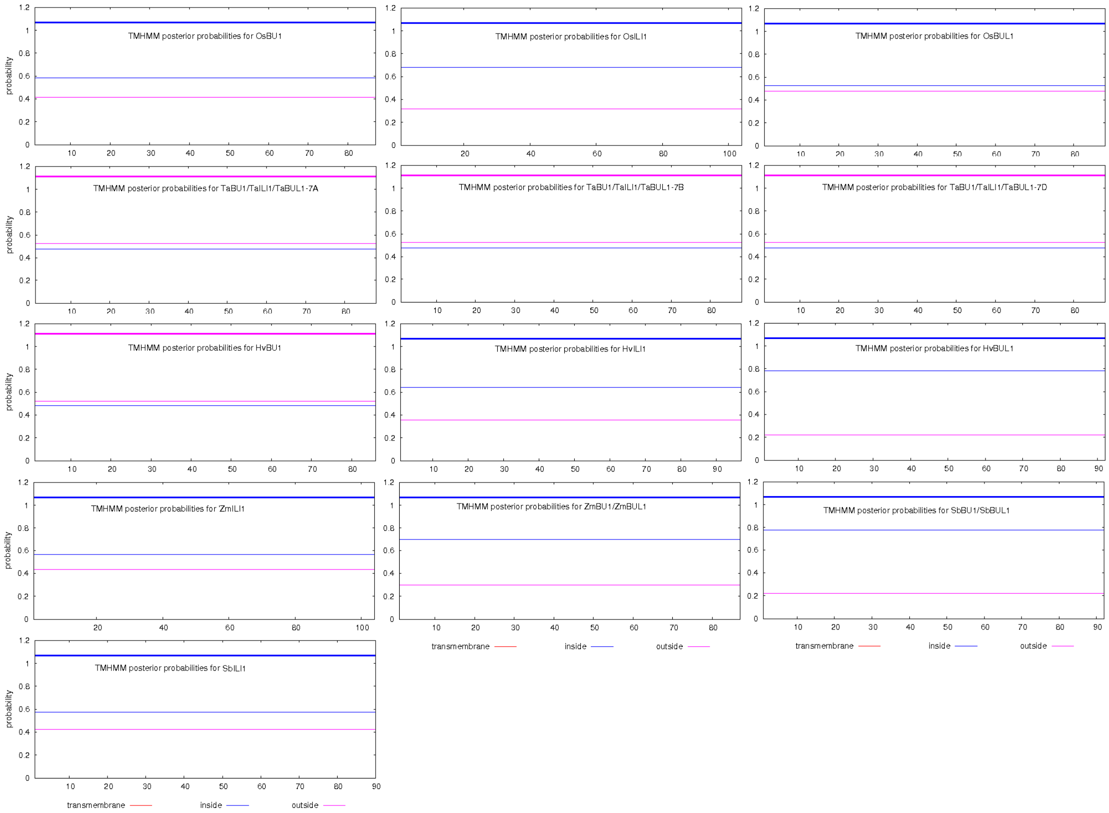

Supplemental Figure S4-19 Transmembrane topology analysis of IBH1 proteins in *T. aestivum*, *H. vulgare*, *Z. mays* and *S. bicolor*.

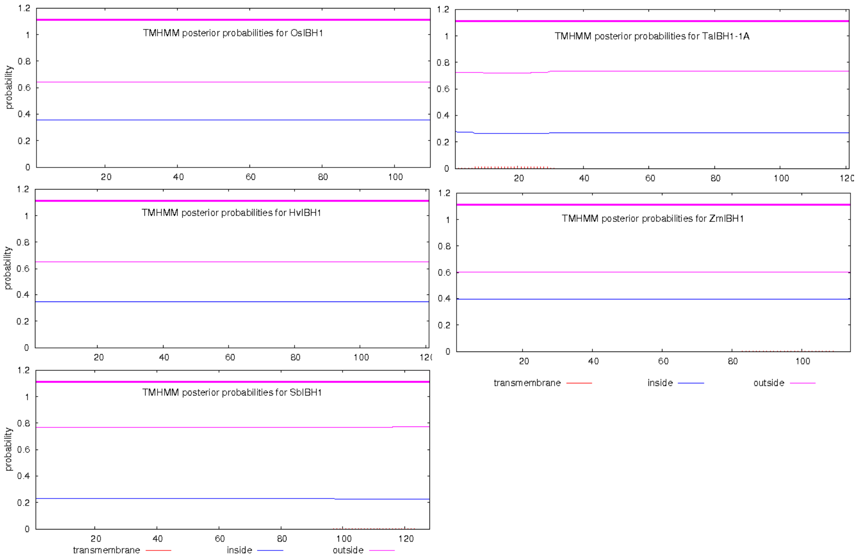

Supplemental Figure S4-20 Transmembrane topology analysis of GRAS19 proteins in *T. aestivum*, *H. vulgare*, *Z. mays* and *S. bicolor*.

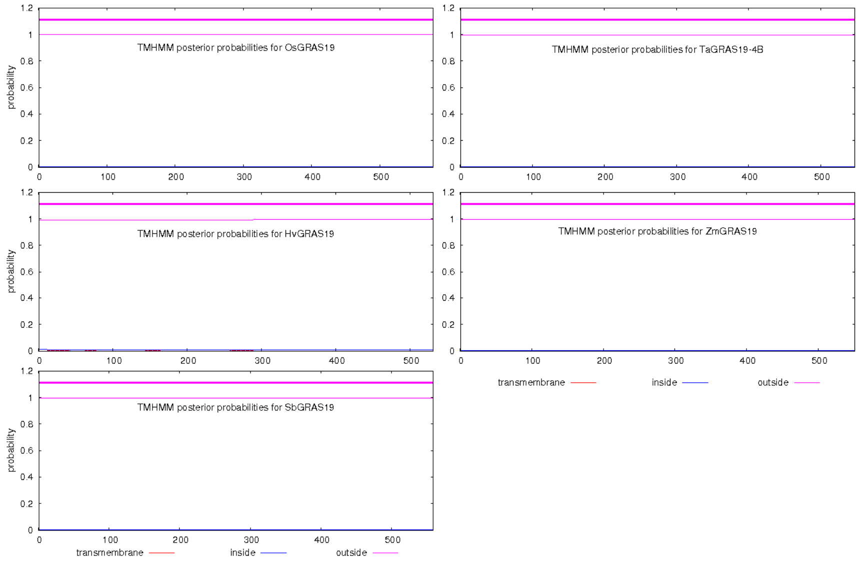

Supplemental Figure S4-21 Transmembrane topology analysis of MADS22, MADS47 and MADS55 proteins in *T. aestivum*, *H. vulgare*, *Z. mays* and *S. bicolor*.

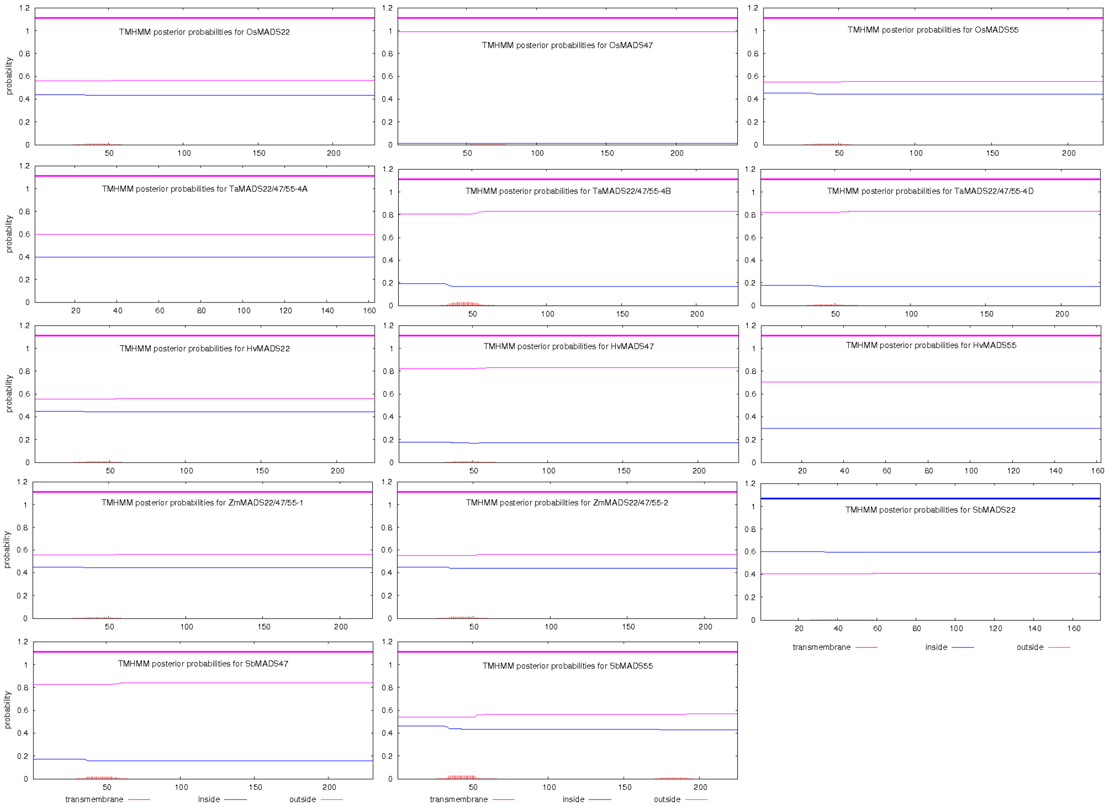

Supplemental Figure S4-22 Transmembrane topology analysis of XIAO proteins in *T. aestivum*, *H. vulgare*, *Z. mays* and *S. bicolor*.

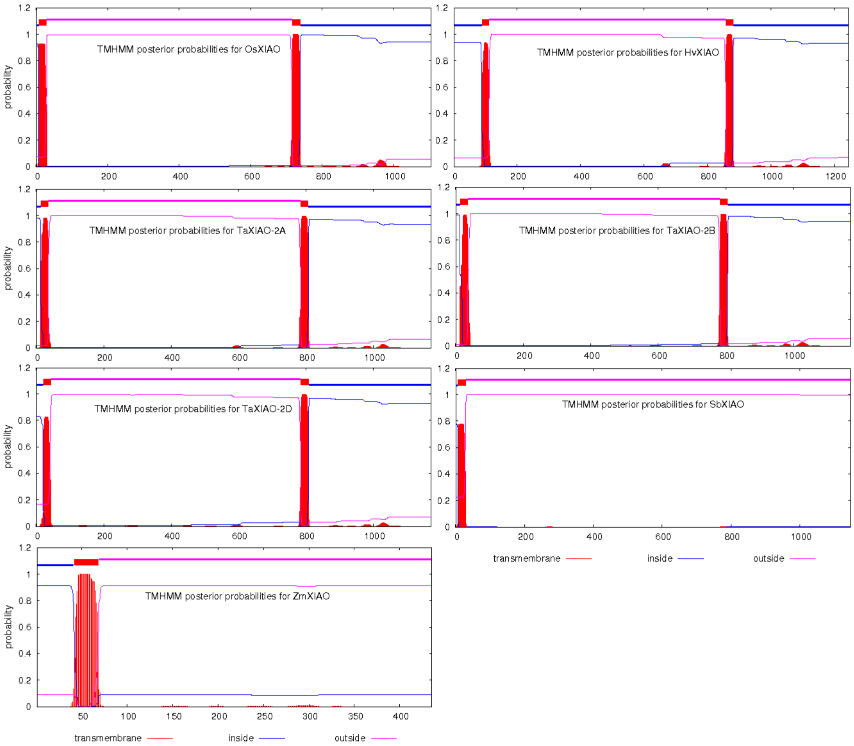

Supplemental Figure S4-23 Transmembrane topology analysis of LC2 proteins in *T. aestivum*, *H. vulgare*, *Z. mays* and *S. bicolor*.

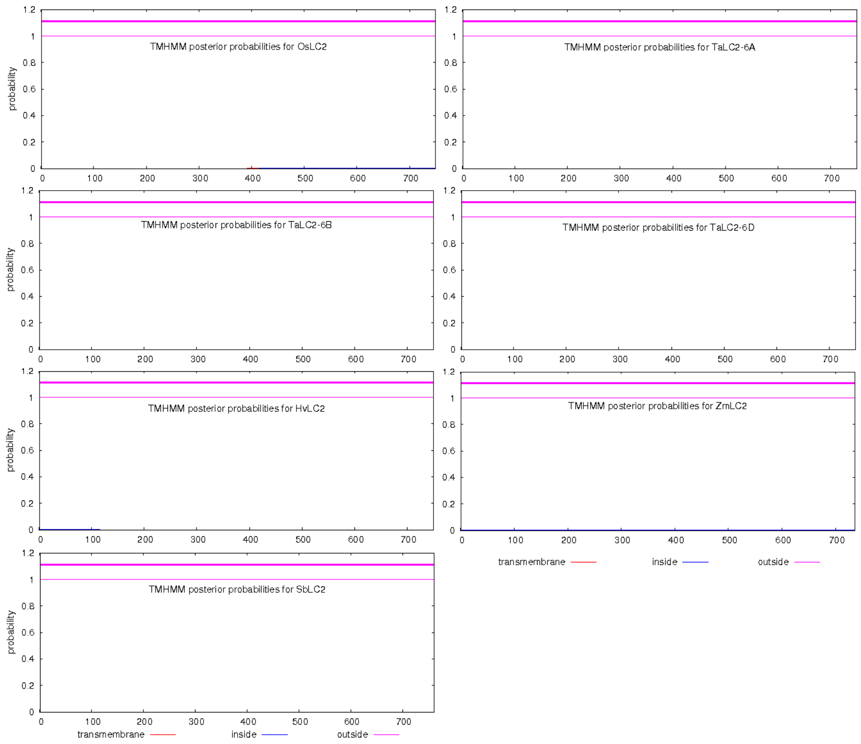

Supplemental Figure S4-24 Transmembrane topology analysis of ARF11 andARF19 proteins in *T. aestivum*, *H. vulgare*, *Z. mays* and *S. bicolor*.

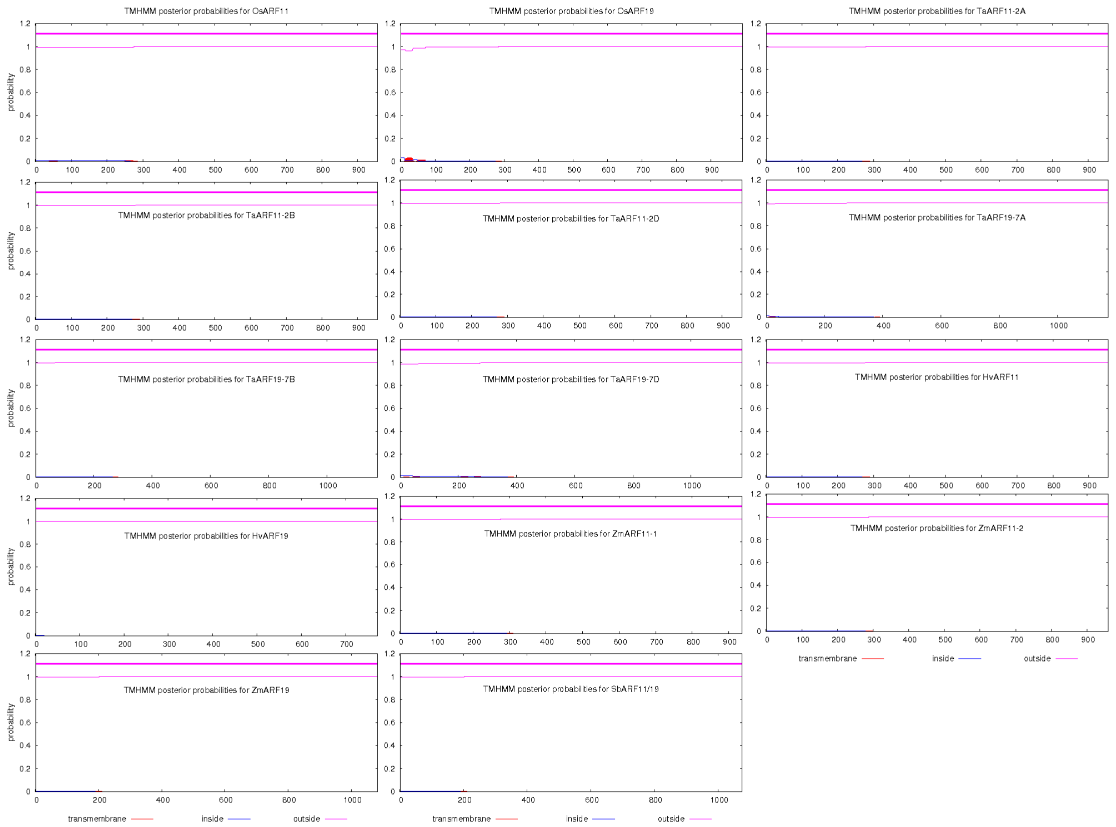

Supplement: Supplementary file 1 [file ijms-23-05551-s001.zip › Figure S4.pdf]
