# Supplementary material for: Genome-Wide Identification of Gramineae Brassinosteroid-Related Genes and Their Roles in Plant Architecture and Salt Stress Adaptation
Source: Int J Mol Sci. 2022 May 16;23(10):5551. doi: 10.3390/ijms23105551 (PMC9146025; doi:10.3390/ijms23105551)

Supplemental Figure S5 Synteny analysis of BR-related plant architecture genes in each gramineae species.

Supplemental Figure S5-1 Synteny analysis of BR-related plant architecture genes in wheat.

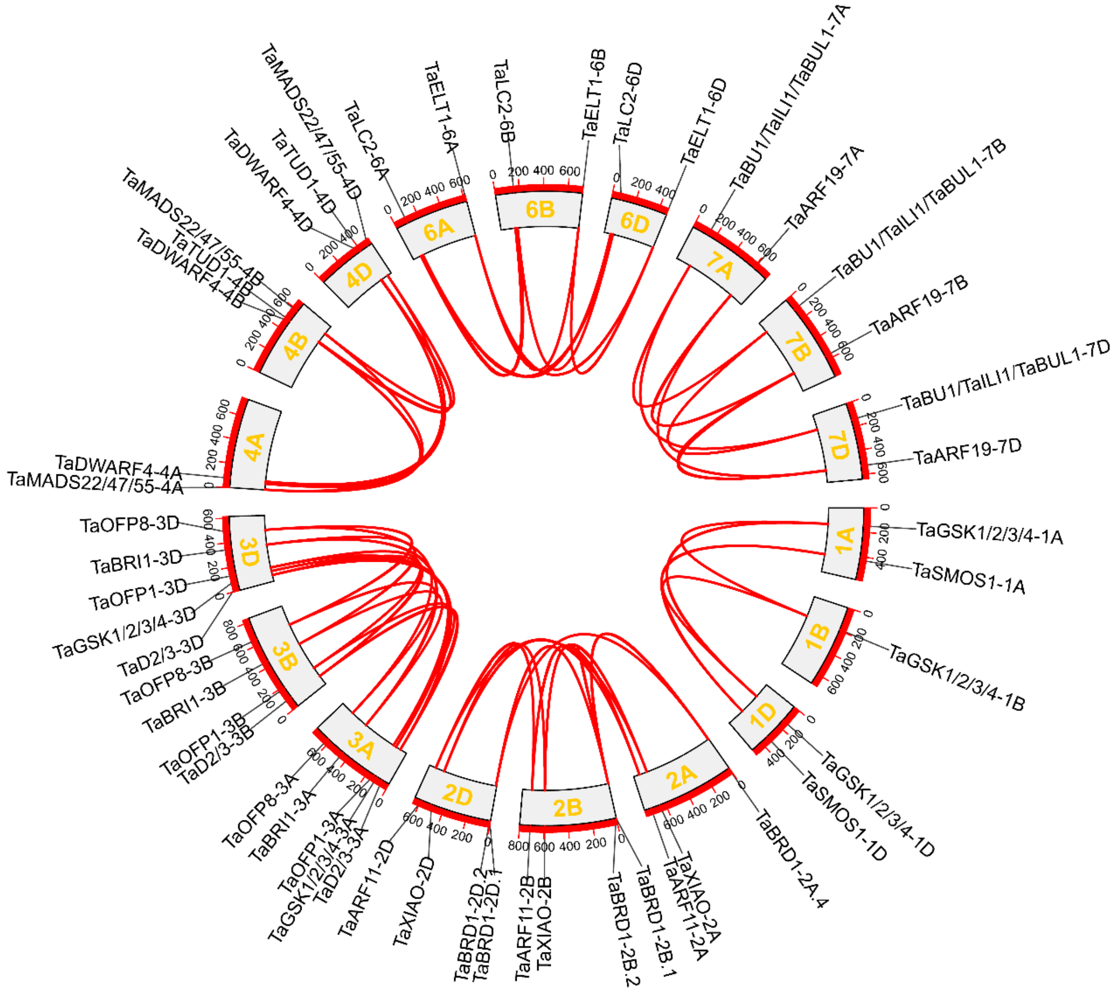

Supplemental Figure S5-2 Synteny analysis of BR-related plant architecture genes in maize.

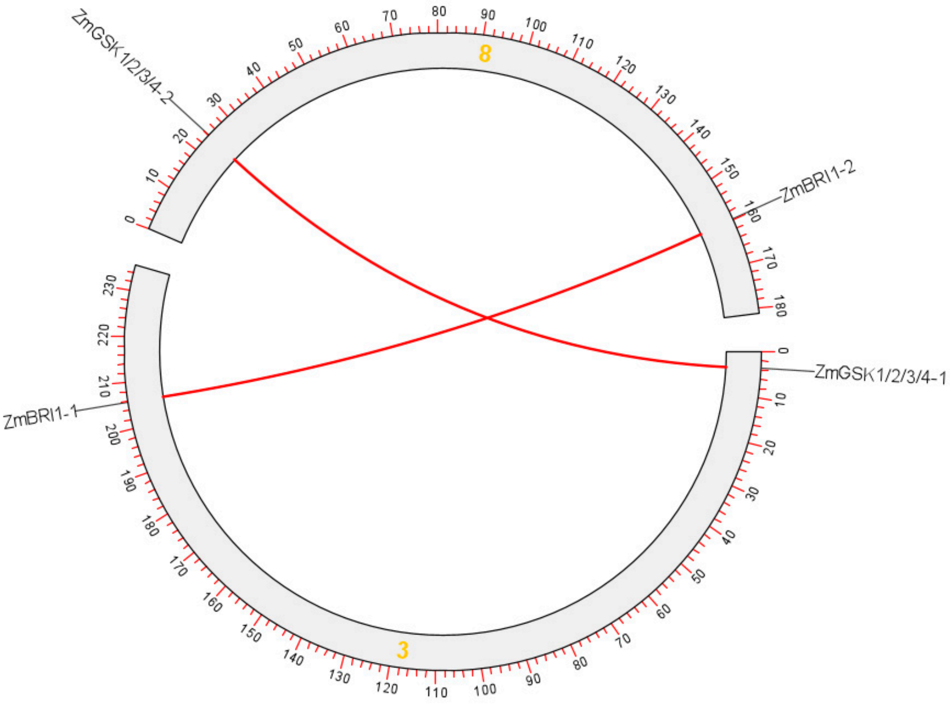

Supplemental Figure S5-3 Synteny analysis of BR-related plant architecture genes in *H. vulgare*.

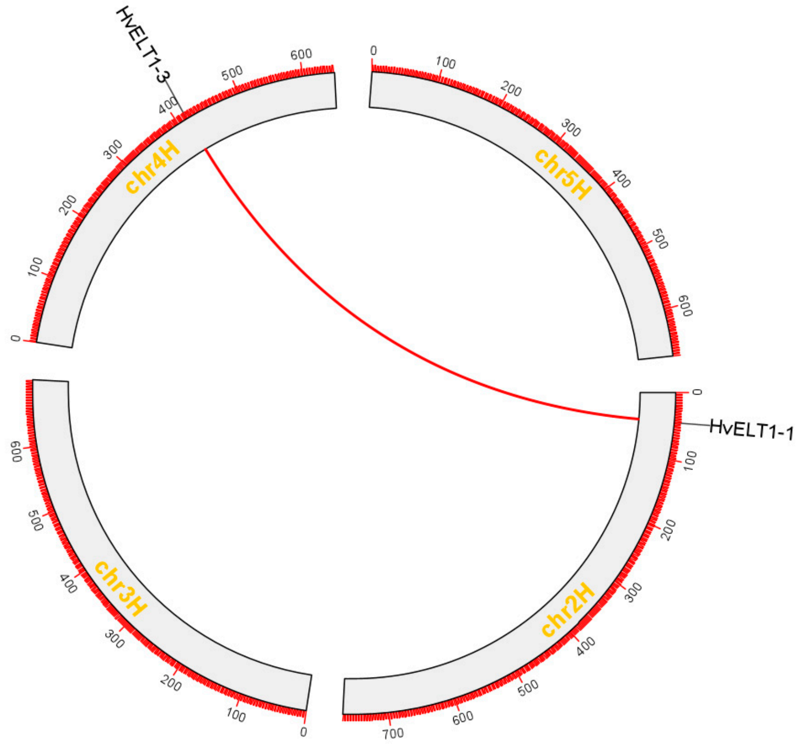

Supplemental Figure S5-4 Synteny analysis of BR-related plant architecture genes in *S. bicolor*.

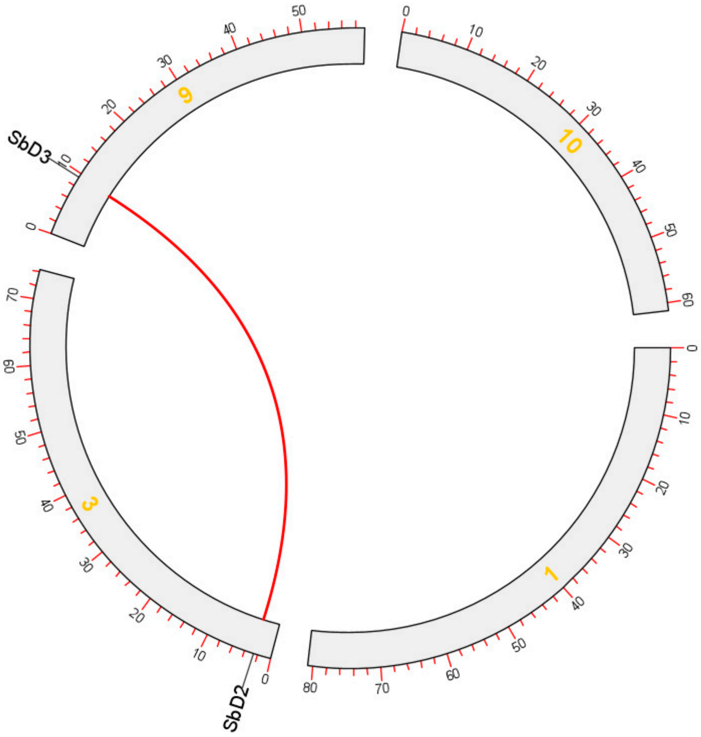

Supplement: Supplementary file 1 [file ijms-23-05551-s001.zip › Figure S5.pdf]
