# Supplementary material for: Genome-Wide Identification of Gramineae Brassinosteroid-Related Genes and Their Roles in Plant Architecture and Salt Stress Adaptation
Source: Int J Mol Sci. 2022 May 16;23(10):5551. doi: 10.3390/ijms23105551 (PMC9146025; doi:10.3390/ijms23105551)

Supplemental Figure S6 Analysis of the synteny among the BR-related plant architecture genes of rice and each gramineae specie.

Supplemental Figure S6-1 Analysis of the synteny among the BR-related plant architecture genes of rice and wheat.

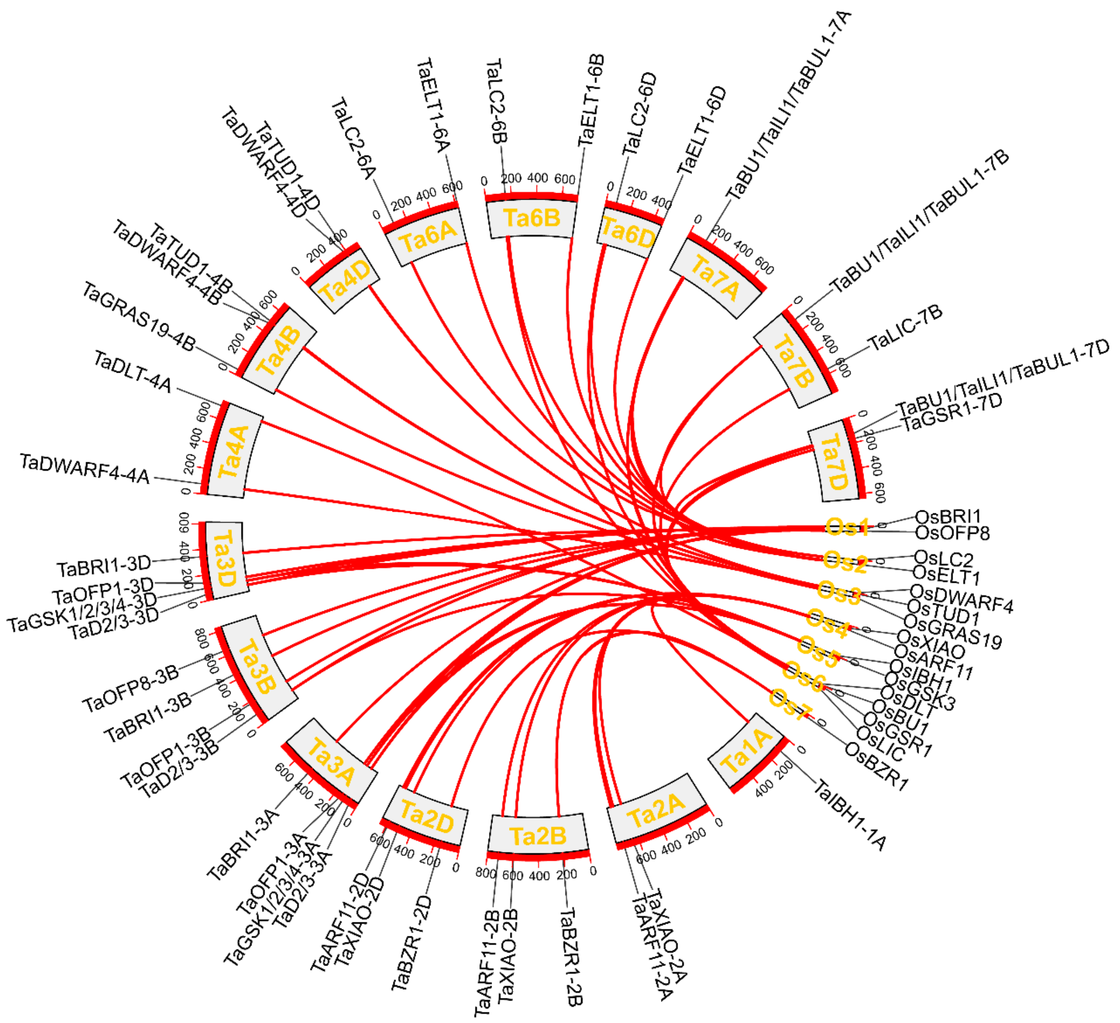

Supplemental Figure S6-2 Analysis of the synteny among the BR-related plant architecture genes of rice and maize.

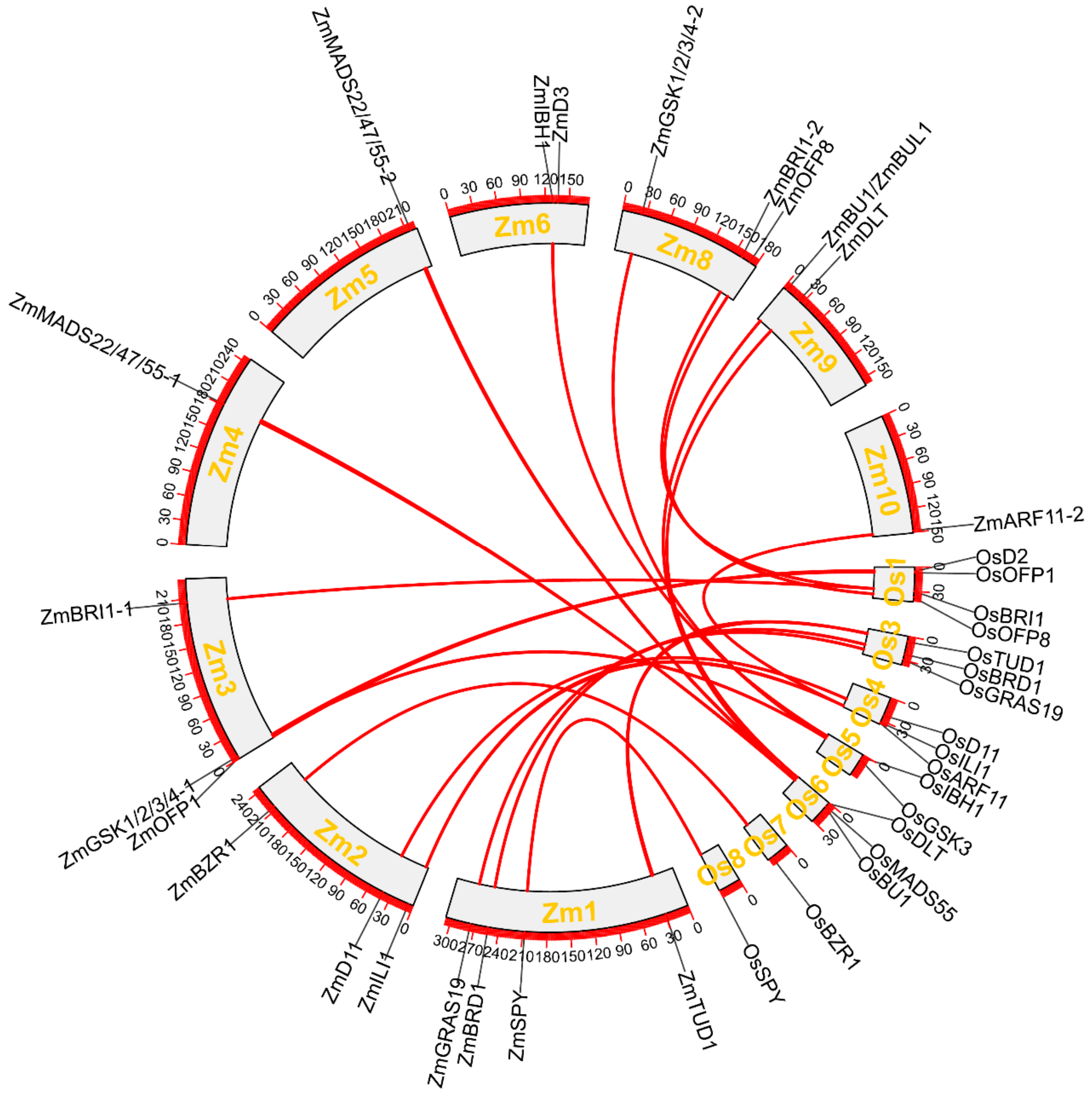

Supplemental Figure S6-3 Analysis of the synteny among the BR-related plant architecture genes of rice and *H. vulgare*.

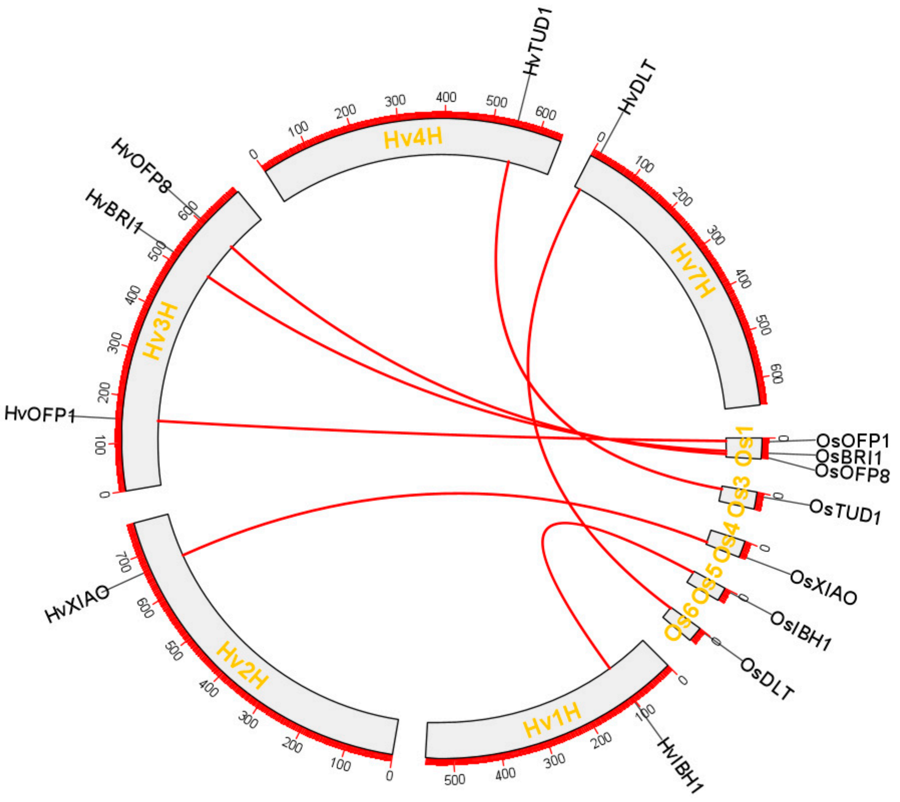

Supplemental Figure S6-4 Analysis of the synteny among the BR-related plant architecture genes of rice and *S. bicolor*.

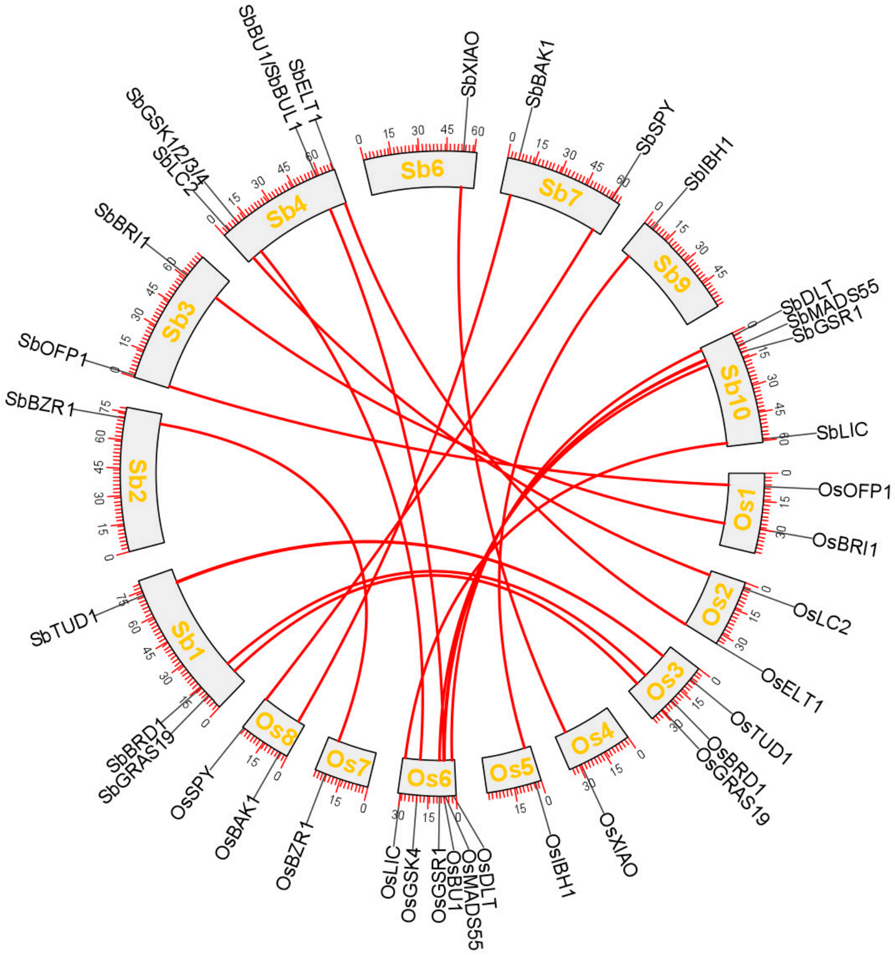

Supplement: Supplementary file 1 [file ijms-23-05551-s001.zip › Figure S6.pdf]
