# Supplementary figures and images for: Genome-Wide Identification of Gramineae Brassinosteroid-Related Genes and Their Roles in Plant Architecture and Salt Stress Adaptation
Source: Int J Mol Sci. 2022 May 16;23(10):5551. doi: 10.3390/ijms23105551 (PMC9146025; doi:10.3390/ijms23105551)

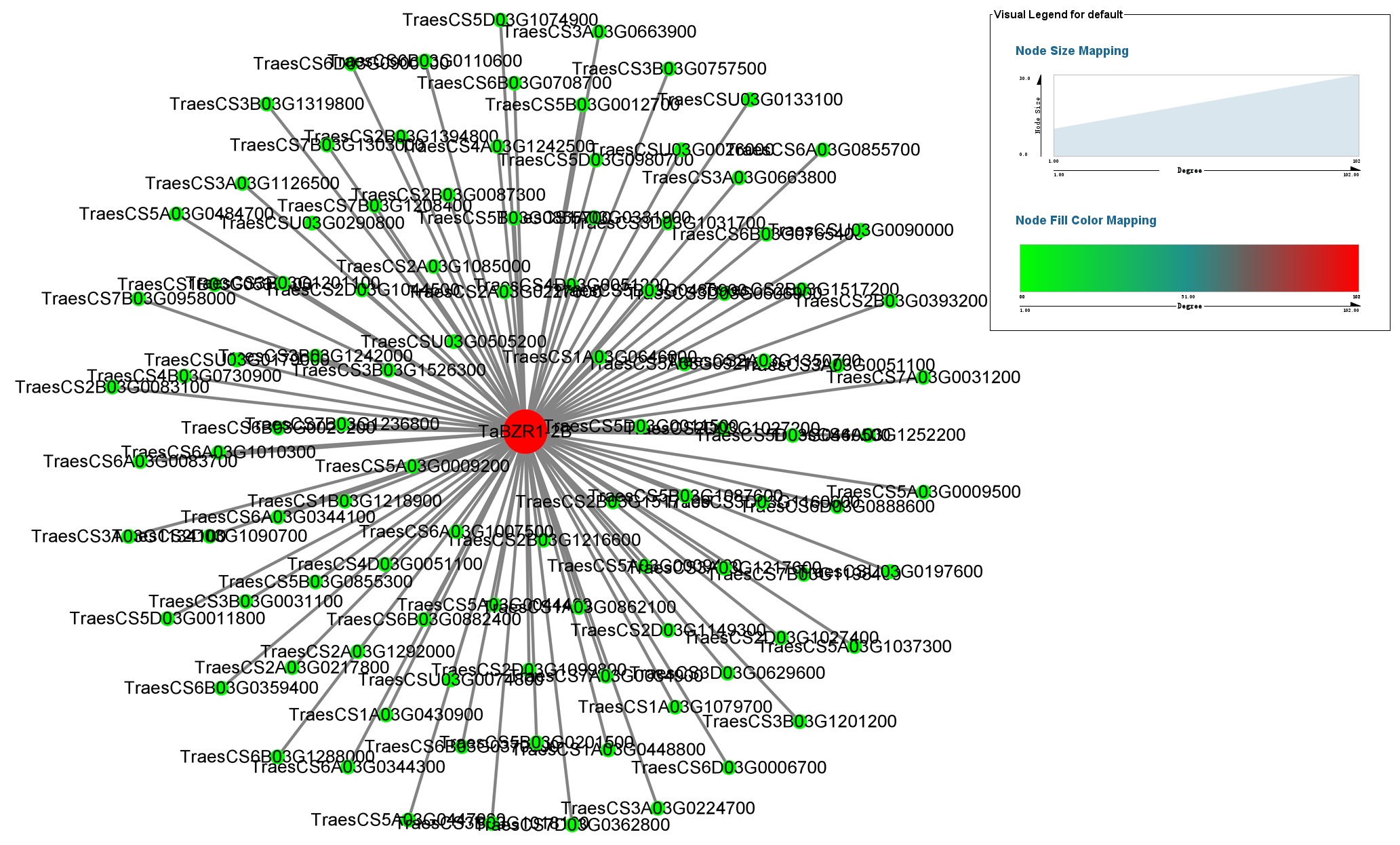

Supplement: Supplementary file 1 [file ijms-23-05551-s001.zip › Figure S10.jpg]

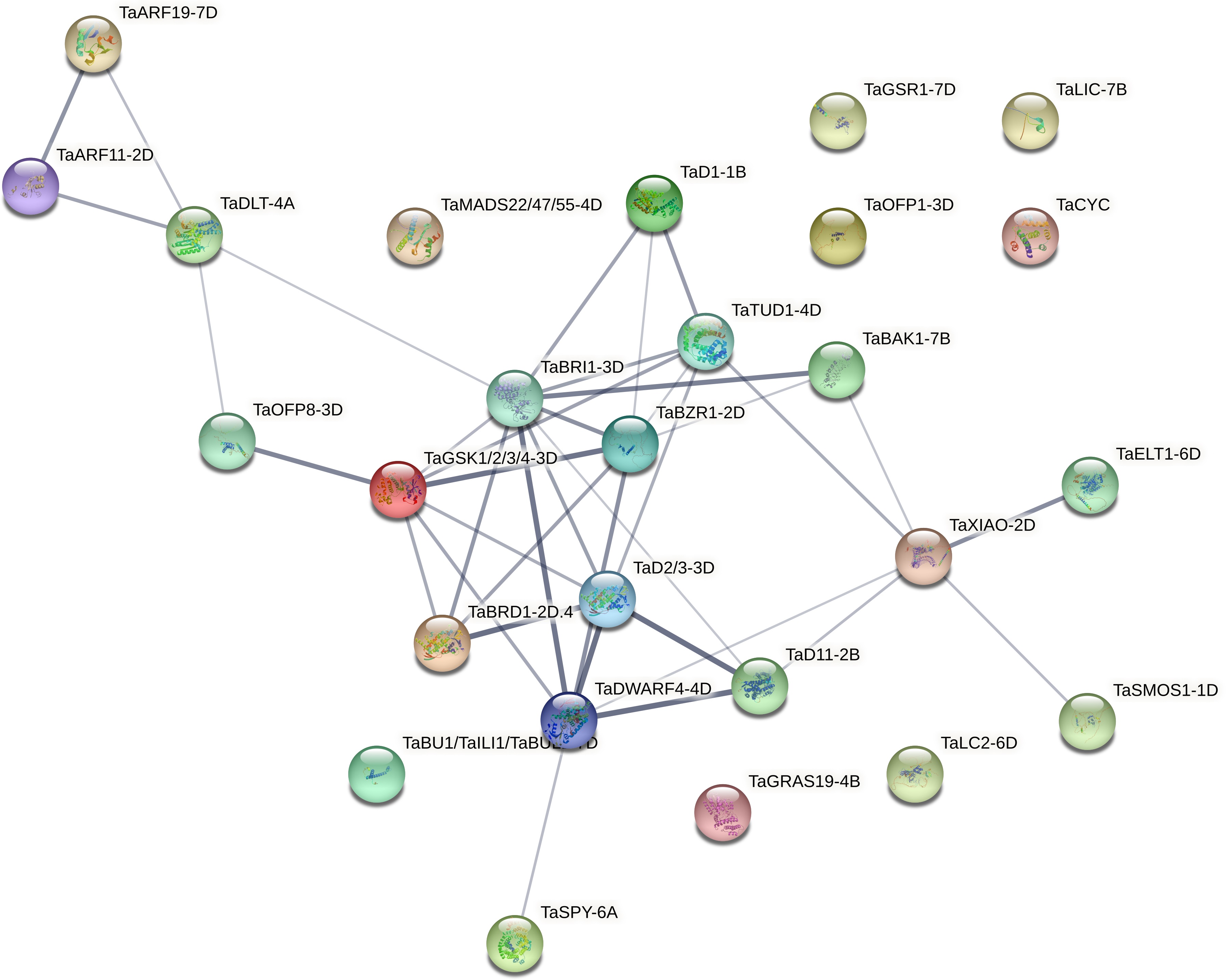

Supplement: Supplementary file 1 [file ijms-23-05551-s001.zip › Figure S8.jpg]

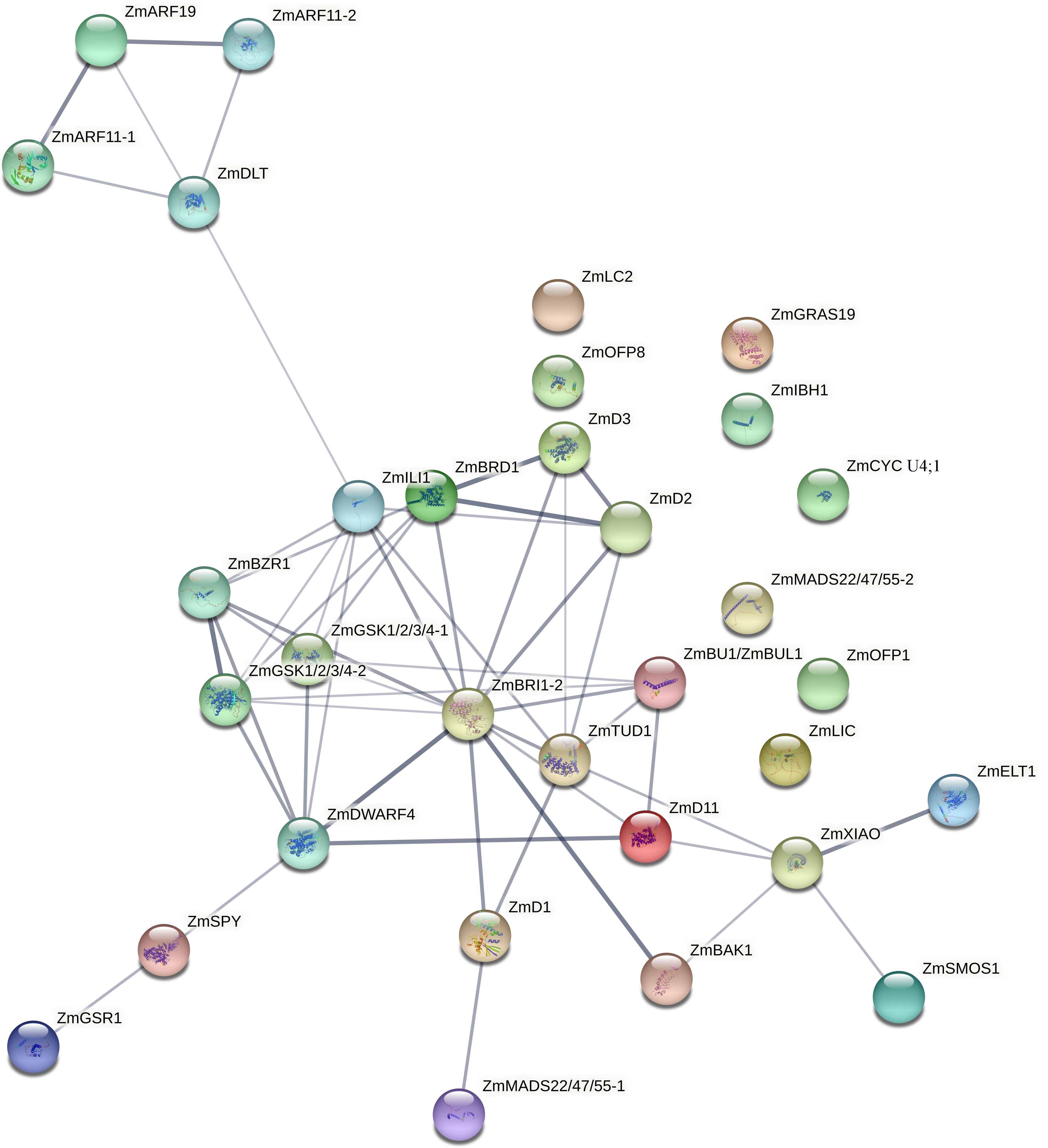

Supplement: Supplementary file 1 [file ijms-23-05551-s001.zip › Figure S9.jpg]
